# Validation of Bacterial Replication Termination Models Using Simulation of Genomic Mutations

**DOI:** 10.1371/journal.pone.0034526

**Published:** 2012-04-03

**Authors:** Nobuaki Kono, Kazuharu Arakawa, Masaru Tomita

**Affiliations:** Institute for Advanced Biosciences, Keio University, Fujisawa, Kanagawa, Japan; The University of Nottingham, United Kingdom

## Abstract

In bacterial circular chromosomes and most plasmids, the replication is known to be terminated when either of the following occurs: the forks progressing in opposite directions meet at the distal end of the chromosome or the replication forks become trapped by Tus proteins bound to Ter sites. Most bacterial genomes have various polarities in their genomic structures. The most notable feature is polar genomic compositional asymmetry of the bases G and C in the leading and lagging strands, called GC skew. This asymmetry is caused by replication-associated mutation bias, and this “footprint" of the replication machinery suggests that, in contrast to the two known mechanisms, replication termination occurs near the chromosome dimer resolution site *dif*. To understand this difference between the known replication machinery and genomic compositional bias, we undertook a simulation study of genomic mutations, and we report here how different replication termination models contribute to the generation of replication-related genomic compositional asymmetry. Contrary to naive expectations, our results show that a single finite termination site at *dif* or at the GC skew shift point is not sufficient to reconstruct the genomic compositional bias as observed in published sequences. The results also show that the known replication mechanisms are sufficient to explain the position of the GC skew shift point.

## Introduction

A circular bacterial chromosome has both a replication origin and a terminus, and replication of the chromosome proceeds bi-directionally from the origin to the terminus [1], [2], [3], [4]. Although the replication termination mechanism is not as well studied as replication initiation (see [5] for review), extensive studies have yielded insight into replication termination in organisms such as *Escherichia coli* and *Bacillus subtilis.* The collision of two opposing replication forks at a region approximately opposite the origin was initially suggested to be the predominant mechanism of termination in these organisms [6]; however, the finding that moving the replication origin does not change the replication terminus in *E. coli* [7], [8] led to the identification of a fork-trapping mechanism involving the 36 kDa Tus protein in *E. coli* [9], and the 14.5 kDa RTP protein in *B. subtilis*, bound to Ter elements [10], [11]. Tus or RTP protein binds to the Ter sites (in *E. coli*, at the sequence 5′-AGNATGTTGTAAYKAA-3′: [12]; in *B. subtilis*, at 5′-KMACTAANWNNWCTATGTACYAAATNTTC- 3′: [13]) and forms a barrier called a fork trap [14], [15]. This fork trap acts as an antihelicase and allows forks to enter but not exit the terminus region [16], [17]. As a result, this complex makes the replication fork stall at the Ter site [18], [19]. In *E. coli*, most Ter sites are located in the terminus half of the genome [9], [20].

The *B. subtilis* RTP protein differs from the *E. coli* Tus protein in both sequence and structure, and these systems are not broadly conserved except in species closely related to *E. coli* or *B. subtilis*. These observations suggest a relatively recent introduction of the fork-trap termination mechanism [21]. Wang and coauthors recently constructed a stain of *E. coli* harboring two origins such that one termination occurred at a Ter site, whereas another terminated speculatively through fork-collision [22]. Similarly, theta-replicating plasmids without fork-trap machinery may terminate by fork-collision; hence, the fork-collision model remains a plausible mechanism for replication termination, especially for species without Tus/RTP analogues.

The bi-directional replication machinery of circular bacterial chromosomes subdivides the genome into two replicating arms, or replichores, with the leading and lagging strands on opposite strands of the DNA duplex. These two replichores experience asymmetric replication-related mutation pressures due to continuous and discontinuous strand synthesis in the leading and lagging strands that results in an excess of G over C in the leading strand [23], [24]. Such strand compositional asymmetry is typically visualized using a GC skew plot with moving windows along the genomic sequence. GC skew is calculated as (C-G)/(C+G), and therefore, its polarity shifts near the replication origin and near the terminus, where the leading and lagging strands switch roles [23], [25], [26]. The cause for this mutational shift from C to G in the leading strand is likely to be multifactorial, and it is still debated [27] with several hypotheses having been proposed to date (see details: [24], [27], [28], [29], [30], [31]).

The most widely accepted hypothesis is that cytosine deamination occurs in the single stranded DNA (ssDNA), resulting in a decrease in C in ssDNA [29], [30] because the lagging strand template exists as ssDNA for a longer time during the replication of the Okazaki fragments in order to serve as the template [32]. Another mutation mechanism that has been proposed is asymmetric transcription-coupled repair [33], which is based on the strand-specific positioning of transcriptionally active genes [34] and their asymmetric distributions [35]. Nevertheless, strand compositional asymmetry, a type of “footprint" of replication-related mutations, is commonly utilized for *in silico* predictions of the replication origin and terminus [36], [37], [38]. Whereas the GC skew shift point accurately correlates with the origin of replication in most bacterial genomes [39], [40], the terminus shift points are often closer to the chromosome dimer resolution (CDR) site *dif* than to the Ter sites [41], [42]. The 28 bp *dif* sequences are widely conserved in bacteria [43], [44], [45] and play a central role in CDR as the binding sites of two tyrosine recombinases, XerC and XerD. In the circular bacterial chromosome, when a recombination event occurs an odd number of times in one DNA replication process, the replicated chromosome forms a concatenated dimer that cannot be segregated into two daughter chromosomes [46], [47]. Therefore, many bacteria have the CDR machinery to separate the dimer chromosome via homologous recombination by XerCD into two monomer daughter chromosomes. The *dif* sites are located near the terminus region [48], [49], but this greater correlation of the *dif* sites with the GC skew remains enigmatic. With their detailed computational study of the skewed oligonucleotides, Hendrickson and Lawrence further confirmed that the skew switch point is closer to the *dif* site than the Ter site. Based on these observations of their “bioinformatically optimized" skew shift point, they speculated that replication terminations are most likely to occur (or had occurred in the course of evolution prior to the introduction of the Tus/Ter system) near the *dif* sites in γ-proteobacteria, Firmicutes and Actinobacteria, to avoid failure of the CDR system [41]. In *E. coli*, previous studies clearly show that the replication forks travel through the *dif* site to reach Ter sites *in vivo* [21], [50], [51]; however, the existence of an unknown replication termination mechanism near the *dif* site remains a possibility in species where the fork-trap associated proteins (Tus or RTP) are not conserved.

We conducted a simulation study to elucidate the relationships between replication termination mechanisms and the genomic compositional bias formed by the replication process. By computationally modeling the above-mentioned replication termination models, namely the fork-trap, fork-collision, and *dif-*stop models, in 65 proteobacterial strains (which have circular genomes, Ter/Tus complexes, and *dif* sites) and in 30 Firmicutes strains (which do not have Ter/Tus complexes), we tested the ability of each model to reconstruct the GC skew graph of existing bacterial genomes. In this paper, we refer to the GC skew calculated from the published genome sequences as “natural GC skew" to distinguish them from artificially constructed GC skew.

## Results

### GC skew formation simulation

**Figure 1:**
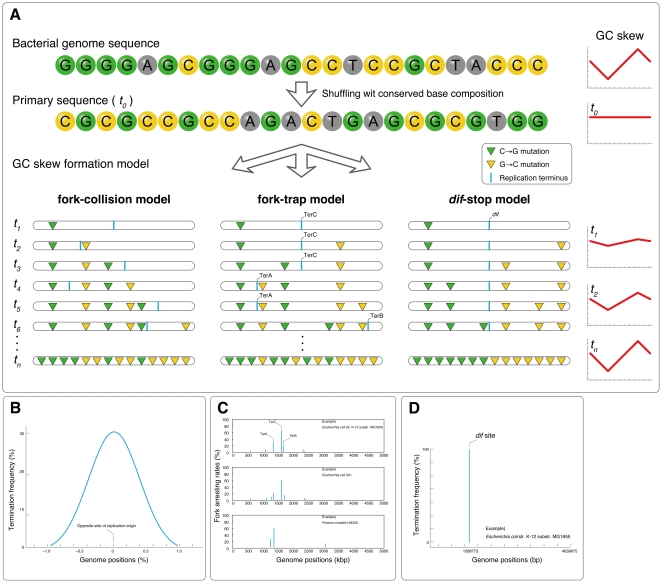
Scheme of GC skew reconstruction simulation. A: A schematic representation of the GC skew reconstruction simulation. The primary sequence was generated based on the shuffled bacterial genome sequence, which had the same base composition as the original sequence. The green and yellow triangles represent the locations of C→G mutations in the leading strand (or G→C in the lagging strand). Graphs on the right show the typical GC skew shape at each simulated time point (*t_i_*). The blue bars represent the replication termini. B: Frequency distribution of replication termini in the fork-collision model. Here, replication terminates near a locus directly opposite the origin, and the position probabilistically fluctuates according to a Gaussian distribution. The distribution was empirically derived from plasmid sequences that are likely to be terminated by fork-collision mechanisms. C: Frequency distribution of replication termini in the fork-trap model in *Escherichia coli* str. K-12 substr. MG1655, *Escherichia coli* IAI1, *Proteus mirabilis* HI4320. Here, replication termination occurs at Ter sites, but different Ter sites have different rates of fork arrest. D: Frequency distribution of replication terminus in the *dif*-stop model in *Escherichia coli* str. K-12 substr. MG1655. Here, all replication terminates at a single finite locus *dif*.

Because GC skew represents the evolutionary footprint of a replication-related mutational bias, we attempted to elucidate the contributions of different replication termination models by computationally reconstructing the GC skew pattern using simulations of strand-biased mutations. Although the specific substitution types and mechanisms are likely to be multifactorial, compositional replication strand bias, with only few exceptions, is strongest for G>C in the leading strand of prokaryotes [27], [52]. Hence, we took the simplest approach to simulating the evolutionary formation of GC skew. We started with shuffled sequence that had no replication strand bias, and we iteratively introduced C→G mutations in the leading strand until the GC compositional bias between the leading and lagging strands was equal to that of existing genomes (Figure 1A). The relative amounts of complementary bases should theoretically reach equilibrium when there is no strand bias [53], [54]; therefore, replication strand bias should be reconstructed using only the replication-related mutation bias. Our simulation involves three principal sets of variables: 1) the initial sequence with no strand bias, 2) the number of simulated mutations (simulation cycles), and 3) the locations of the replication origins and termini. Although many prokaryotic genomes exhibit significant replication strand bias, the relative amounts of complementary bases are close to equilibrium across the entire genomic sequence. We generated an artificial genomic sequence with no replication strand bias by shuffling the observed sequence while maintaining its overall composition. The number of simulated mutations, or the number of simulation cycles, was determined as the absolute difference between the number of G and C bases in the leading/lagging strands across the whole genome. For example, given an imaginary genome sequence of 1 Mbp with equal amounts of all four bases, the genomic G or C content would be 250,000 bp each. Because the leading strand of this genome would be biased toward G, the quantities of G and C bases would be 260,000 bp and 240,000 bp, respectively. Here, the absolute difference in G or C content, 10,000, is the number of C→G mutations required to reconstruct the GC skew, and this number also represents the number of simulation cycles. The last of the three sets of variables, the location of the replication terminus, is the most central part of our simulation study. The replication strand bias predominantly causes enrichment of G in the leading strand, but the definitions of the leading and lagging strands change under different replication termination models. This is because the locations of replication termination vary according to the models. For example, the fork-collision model results in probabilistic termination within the region approximately 180 degrees opposite the origin, whereas the fork-trap model involving the Ter/Tus system terminates at multiple but defined finite locations. Likewise, if replication termination occurs near the *dif* site or near the GC skew shift point, the replication terminus becomes a single finite location. In this simulation study, we assess the reproducibility of the GC skew graph using varying replication termini inferred by different replication termination models.

We first tested the applicability of such simulations using the *E. coli* K-12 genome. In *E. coli*, the numbers of G and C bases in the whole genome were 1,176,923 bp and 1,179,554 bp, respectively, and the numbers of G and C in the leading strand were 1,216,043 bp and 1,140,434 bp, respectively. Therefore, the number of simulation cycles was determined to be 39,120 based on the difference between the two compositions. Shuffled initial sequence with no replication strand bias was generated while maintaining the overall genomic base composition (A: 24.62%, T: 24.59%, G: 25.37% and C: 25.42%). In this first validation, the replication terminus was defined at a finite location at the GC skew shift point (1,550,412 bp). This was performed to observe whether this simplistic simulation could reconstruct the GC skew graph. The similarities between the artificial GC skew and the natural GC skew graphs were evaluated by root mean square error (RMSE) as well as by the GC skew index (GCSI), which quantifies the degree of GC skew. GC skew is generally visible when GCSI>0.05 [55]. Although the GC skew shape in the initial sequence (*t* = 0) was almost completely flat and had a high RMSE value (GCSI = 0.007 and RMSE = 6.982), the GC skew-like shape was gradually formed as the simulation cycles progressed. When the simulation reached 39,120 cycles (the maximum number of iterations), the artificial GC skew shape showed least difference from the natural GC skew as calculated by RMSE (artificial and natural GCSIs were 0.098 and 0.097, respectively, and the RMSE between them was 0.025). The GC skew shapes found after different numbers of simulated cycles (*t* = 0, 10,000, 15,000, 20,000, 25,000 and 39,120) are described in Figure S1. Probabilistic errors (or standard deviations) associated with the Monte Carlo simulation procedure used for sequence shuffling and simulating mutations were negligible because the standard deviation was less than 0.0256 (Figure S2).

### Construction of three replication termination models

Our simulation study involves three replication termination models: fork-collision, fork-trap, and *dif*-stop. As described above, these models define the positions of the leading and lagging strands, and they are mathematically modeled based on the existing knowledge of replication termination, with parameters empirically derived from genomic data.

In the fork-collision model, replication terminates when the two opposite replication forks meet by chance at the far end of the circular chromosome. Because the collision occurs randomly, the termination positions should follow a probabilistic distribution. We derived the distribution by observing the positions of the GC skew shift points in replicons that are highly likely to be terminating solely by fork-collision: namely, plasmids that have been replicated bi-directionally with theta replication machinery and lack the Ter/Tus complex (for fork-trap model) and the XerCD/*dif* system (for *dif*-stop model). Using 98 plasmids, the distribution was fit to a Gaussian distribution (*p*<0.295 by Kolmogorov-Smirnov test) centered close to part of the genome opposite from the origin (Figure 1B). The distribution was thus derived and normalized to the genome size (see Materials and Methods for detailed parameters), which was used to define the termination position in each simulated cycle of the fork-collision model.

In the fork-trap model, replication terminates specifically at Ter sites (the sites where Tus proteins bind), but each Ter site individually allows a certain fraction of the incoming replication forks to pass with different rates. We therefore needed to obtain the probabilistic ratio of fork trapping at each Ter site. Based on the time and probability of accidental stalling of replication forks at sites other than Ter, on the positional relationship among different Ter sites, and on the leakiness of each Ter site (see Materials and Methods), we could calculate the frequency distribution and computationally determined the fork-trap rates at each Ter site (Figure 1C).

Unlike the two models described above, the *dif*-stop model involves predictable termination at a single finite position without any probabilistic fluctuations. We sought to determine the exact positions of the *dif* sites in bacterial genomes using computational predictions (Figure 1D). We have previously reported an accurate and comprehensive prediction of *dif* sites in 641 bacterial genomes using a recursive hidden Markov model method [45], and all positions of *dif* sites used in this work were obtained from the database accompanying that previous study (http://www.g-language.org/data/repter/). Similarly, as a control, we implemented a model that terminates at the GC skew shift point instead of at the *dif* site.

### Evaluation of the replication termination models

**Figure 2:**
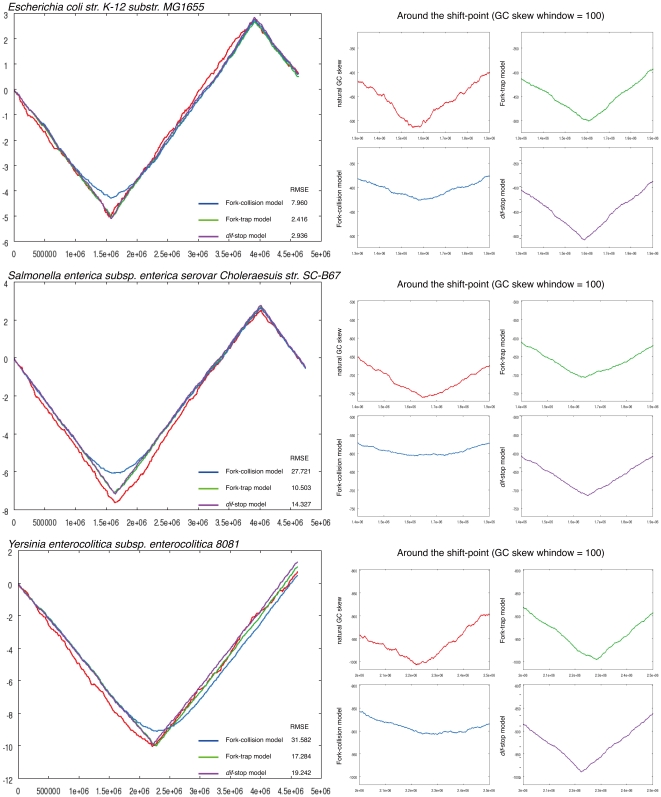
Examples of simulated GC skew. Examples of the overall shapes around the GC skew shift points (see Figure S3 for comprehensive results from all organisms used in this work). The left figures show the overall GC skew graph, and close-ups of the regions around the shift point are shown to the right. In the right set of graphs, red, green, blue and purple lines show the natural GC skew, fork-trap model, fork-collision model and *dif*-stop model, respectively.

**Figure 3:**
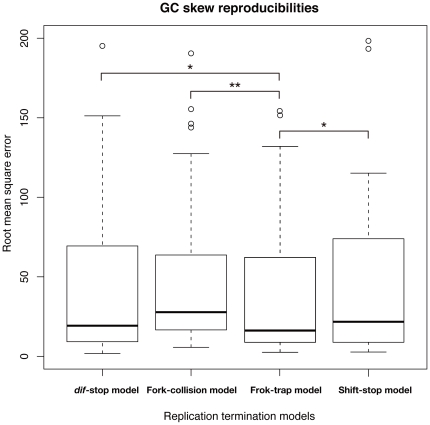
Comparison of RMSE scores in four models. Boxplot of the RMSE scores for four models, representing the similarities between simulated and natural GC skews in the four models (in 65 bacteria). The *p* values were calculated by a Wilcoxon test, * *p*<0.05, ** *p*<0.01.

We tested the validity of the aforementioned models with 65 Proteobacterial genomes, including those of *E. coli* strains and others that have circular chromosomes, Ter/Tus systems, *dif* sites and XerCD homologues as well as a compositional bias of GCSIs≥0.1. Typical examples of the simulated GC skew graphs are provided in Figure 2 (all simulation results in target organisms are shown in Figure S3). Whereas there was no significant difference between the *dif*-stop and fork-collision models (*p*<0.069, Wilcoxon test), the fork-trap model showed significant differences from other models (*dif*-stop model and fork-collision model, *p* = 0.011 and *p* = 0.007, respectively, Wilcoxon test; Figure 3). Interestingly, even the control model scored significantly lower than the fork-trap model (*p*<0.021, Wilcoxon test; Figure 3), and the control model, by naïve expectation, should best reproduce the GC skew graph because it terminates replication at the GC skew shift point. Of the three models tested, the fork-trap model seems to best explain the existing GC skew shapes.

**Figure 4:**
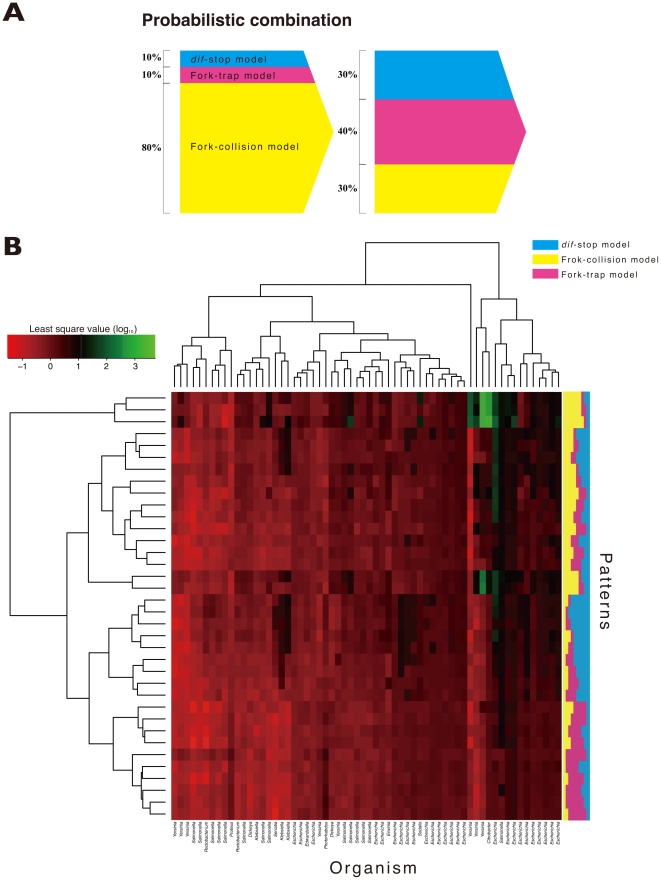
Heat map of RMSE scores for probabilistic combination model. The conceptual scheme (A) and heat map of RMSE scores (B) for probabilistic combination of replication termination models. The x-axis represents the 65 organisms, and the y-axis represents the combination patterns. Each color represents one of the three models (blue = *dif*-stop model, yellow = fork-collision model and red = fork-trap model), and the width of colored regions represents their probability (B). The scales are logarithmic.

According to the above result, the fork-trap model is shown to be the most appropriate model to explain the existing GC skew. However, replication termination *in vivo* is certainly not as simple as this simulation that utilize only a single termination machinery. Although one type of termination machinery may be dominant in the existing genomes, other machineries could co-exist at a much lower prevalence. Previous studies have suggested or identified numerous fork arresting mechanisms besides the Ter/Tus system, such as those by transcription-replication collisions and inactivation proteins [56], [57], [58], [59], and by proteins bound to the *dif* site [41]. Our simplistic models described thus far can only account for idealistic situations where replication terminates by only one mechanism, and a more realistic simulation requires the probabilistic combinations of these situations and models. In order to examine the contribution ratio of each model to construct the GC skew, we conducted further evaluations of the replication termination models in a hypothetical probabilistic combination, where the termination models is assumed to coexist under certain probabilistic preferences (Figure 4A).

To determine the probabilistic ratio of each type of machinery, we tested all possible combinations using the three models. For computational efficiency, ratios were incremented by units of 10% of the total number of simulated cycles, and consequently, 36 patterns were assessed. In this case, none of the different combinations significantly affected the reproducibility of the GC skew (Figure 4B). Nevertheless, combination model often resulted in lower RMSE compared to simulations using only one of the three termination models independently.

The best probabilistic combination differed among bacterial species. We extracted patterns that performed well across all of the 65 genomes used in this work, among the 36 probabilistic combinations tested. The best pattern of the probabilistic combinations was 10%-70%-20%, in the order of fork-collision, fork-trap and *dif*-stop models. The probabilistic combination model showed less RMSE values than *dif*-stop and fork-trap models (*p*<0.001, Wilcoxon test; Figure S4 and S5).

### Simulations in species lacking fork-trap machinery

Lastly, we conducted the same analysis for species in other phyla to confirm the observed model preferences. For these analyses, we used 30 Firmicutes species that lack Tus and RTP homologues (and therefore are presumed to lack fork-trap machinery). As a result, significant differences were found between the *dif*-stop and probabilistic combination models (*p*<0.001, Wilcoxon test; Figure S6). Due to the lack of defined replication termini of the Tus/Ter system, the change of the skew is presumably more U-shaped than V-shaped in these species. This is partly suggested by the significantly higher RMSE (*p*<0.01, Wilcoxon test) of the *dif*-stop models in Firmicutes (Figure S6) in comparison to those in Proteobacteria (Figure 3).

## Discussion

In circular bacterial chromosomes, *in vivo* studies clearly show that replication is terminated by fork-trap mechanisms involving the Ter/Tus system, which impedes fork progression at specific sites. However, the genomic compositional bias shaped by replication-related mutation bias, which is an evolutionary footprint of the replication machinery, has a shift point of compositional polarity at a site closer to *dif* than Ter. In this study, we took a theoretical approach to elucidate this paradoxical relationship between the replication-related genomic compositional bias and the replication termination mechanism in bacteria. To that end, we conducted a simulation study employing multiple replication termination models. Three main models, namely fork-collision, fork-trap, and *dif-*stop, as well as one control model that assumes replication termination at the GC skew shift point were tested by computationally reconstructing the GC skew shape in 65 bacteria. Different combinations of these models were also analyzed. Based on the results, the reproducibility of simulated GC skew was highest in the fork-trap and fork-collision models (in comparison to that of original genome sequence). Surprisingly, it was much lower for the *dif*-stop model and the control model. Our result therefore supports previous *in vivo* studies [51] that favor the fork-trap model as the working replication termination model. Although not intuitively obvious at first sight, the probabilistic usage distributions of the Ter sites better explains the current GC skew shape than the location of the *dif* site.

The simulation method for GC skew reconstruction used in this work was based on the most simplistic approach. The procedure mutates a C to a G in the leading strand for each simulation cycle. We have two justifications for this approach. First, although the specific types and causes of mutations introduced by the replication process are likely to be multifactorial and complex, the resultant compositional bias is predominantly in the direction of C→G in most bacteria [27], as observed in existing genomes. Second, previous discussions regarding the positioning of Ter, *dif*, and the GC skew shift point were based on the GC skew graph, which does not contain any information about AT composition. Therefore, we have limited our discussions to the reconstruction of the GC skew graph, which only requires the consideration of C→G mutations. However, one other factor that should be considered is the positions of the coding regions. Coding strand bias is as high as approximately 78% in the leading strand in Firmicutes or *Mycoplasma* [60], [61], [62], and the GC skew is mostly pronounced only in the third codon positions [35]. On the other hand, the 65 Proteobacteria used in this work have relatively little coding strand bias (averaging 58% in the leading strand), and mutations do not avoid the coding region; they occur all over the genome in these species [52].

In this work, we have simulated the GC skew formation using the whole genome sequence, without excluding any sequences. This is because, in theory, strand bias effects of mutations induced by other mechanisms than replication should cancel out, unless the mechanism itself is related to replication [54]. In *E. coli* and γ-Proteobacteria utilized in this work, gene orientation bias is almost even (54.43% in the leading strand in *E. coli* K12), and therefore transcription/translation-related mutation bias should have minimal effect on the GC skew in these species. On the other hand, local regions of genomes and especially the coding sequences are nonetheless subject to other types of mutations than replication, and therefore we have conducted additional validations to confirm such effects. For this purpose, we have repeated all three simulations (*dif*-stop, fork-collision, and fork-trap model) using only the third positions of the codons and intergenic regions (hereafter referred to as GC skew (GC3/non-coding)), in addition to the GC skew using whole genome sequences: GC skew (all). As expected, in both simulations, whether using the whole genome or only GC3/non-coding regions, the overall results did not change. The RMSE values showed similar tendencies, where the RMSE medians were 34.980, 37.516, and 1.493, for *dif*-stop, fork-collision, and fork-trap model, respectively in GC skew (GC3/non-coding), whereas those of GC skew (all) were 19.243, 27.772, and 16.439, respectively. Figure S7 shows the GC3/non-coding version of Figure 3. Overall, both simulations show that the fork-trap model can better explain the existing GC skew shape, rather than the *dif*-stop model.

For the fork-collision model, we determined the positions where the forks collide by observing the fluctuations of the GC skew shift point in plasmids. Plasmids were used rather than chromosomes for several reasons. First, the chromosomal sequences are not suitable for determining these parameters because replication termination in these replicons involves mechanisms other than fork-collision. Moreover, long chromosomal sequences also undergo large-scale restructuring, typically by horizontal gene transfer or inversion [31]. Inversions disrupt gene order and the orientations of oligonucleotides [63], [64], and the genomic islands acquired through horizontal gene transfer likewise change the genomic structure; they can be as large as 10,000 bp upto 1 Mbp [65], [66]. We selected bacterial plasmids that depend on the host replication machinery based on the absence of the *repC* gene, which is required for rolling circle replication [67] and based on the lack of Ter or *dif* sites. In these plasmids, the putative locations of frequent fork collisions obey a clear Gaussian distribution centered at a position directly opposite that of the putative origin determined by the GC skew shift point, as described in Figure 1B, suggesting that replication termination occurs probabilistically through collision and not by the action of specific terminating proteins. The speed of fork progression in both replichores seems to be similar, and the replichores show almost identical base compositions (*R* = 0.994).

The probabilistic distributions of the rates of fork trapping at each Ter site in each bacterium were calculated from three biochemical evidences: the time and probability of accidental stalling of replication forks at sites other than Ter, the positional relationship among different Ter sites, and the leakiness of each Ter site. Based on these evidences, we could calculate the pausing ratio at each Ter site. Furthermore, in order to validate such pausing rates, we compared these biochemical parameters with a computationally determined pausing ratio by means of parameter search that best reconstructs the natural GC skew using all possible patterns of fork pausing at various Ter sites (see Materials and Methods). As a result, the calculated pausing rates based on experimental data were very similar with the optimized pausing rates (*R* = 0.725, Spearman rank-correlation coefficient, Figure S8). Fork-trap model scored best among other replication termination models using either of these parameters.

The locations of *dif* sites strongly correlate with those of the GC skew shift points (*ρ* = 0.736) [45], and these distances are closer than the nearest Ter sites and the loci directly opposite the replication origin (the average distance from the GC skew shift point to a *dif* site is 0.39%, to the nearest Ter site = 0.68%, to the side opposite the origin = 2.61% in 65 targeted bacteria). Therefore, by naive expectation, replication should terminate near the *dif* sites to produce the GC skew graph seen in existing genomes. However, our simulation study shows that replication termination at a single finite locus cannot accurately reconstruct the GC skew shape. In fact, a single finite termination model results in a highly acute shift point, but the actual shift point is less acute and more rounded. Such a shape can only be reproduced with probabilistic models (the fork-trap and fork-collision models) (Figure 2). Therefore, the probabilistic balance of replication termination results in the current shift point position, and the *dif* sites seem to be co-evolving and taking advantage of the genomic compositional bias to be near this probabilistic center of replication termination loci (which allows for efficient CDR). In fact, FtsK translocase locates the *dif* site and recruits XerCD recombinase to the site through the guidance of a highly skewed G-rich oligomer, known as the KOPS [68], [69], [70], taking advantage of the genomic compositional skews and the distribution of the skewed oligomers [71], [72]. Therefore, our simulation study suggests that *dif* sites are not shaping the GC skew by terminating replication at this specific locus, but rather, the GC skew shift-point shaped by the replication termination machinery is affecting the location of *dif* sites. This is in agreement with *in vivo* studies [21], [51] and with our previous *in silico* study, showing that the distance between the *dif* site and GC skew shift point is not correlated with GC skew strength [45].

Finally, we confirmed the contribution ratio of each model to construct the GC skew using probabilistic combination model. The most optimal combination validated by RMSE was the 10-70-20% (fork-collision, fork-trap, and *dif*-stop model, respectively) in probabilistic combination. In previous studies, it has been indicated that the replication fork arrest occurs in 18 to 50% of replication cycles with several factors, including transcription-replication collisions, fork trap with Ter/Tus complex, or by inactivation proteins [56], [57], [58], [59]. In addition to these studies, Maisnier-Patin et al., reported an estimate of at least 20% of all replication forks are stalled and require replisome reassembly during the replication process [73]. Furthermore, Hendrickson and Lawrence speculate that the cleavage of *dif* might occasionally block the progression of forks [41]. Therefore, our probabilistic combination simulation yielding 10-70-20% ratios for fork-collision, fork-trap, and *dif*-stop model seems to fit reasonably well to explain the contributions of different fork-termination mechanisms.

## Materials and Methods

### Software and sequences

All analyses in this study were conducted using programs written in Perl with the G-language Genome Analysis Environment, version 1.8.13 [74], [75], [76]. Statistical analysis and visualizations were performed using the R statistics package, version 2.10.0 (www.R-project.org). This study targeted 65 Proteobacteria strains that have circular genomes, Ter sequences, Tus proteins and *dif* sites, as well as 30 Firmicutes strains that have no Ter/RTP homologues. The existence of Ter sequence was confirmed with the “oligomer_search" function of the G-language GAE, and RTP homologues were determined using the KEGG (Kyoto Encyclopedia of Genes and Genomes) Orthology database (KO; [77]). The genomic and plasmid sequences were obtained from the NCBI FTP Repository (ftp://www.ncbi.nlm.nih.gov/Ftp).

### Selection of bacteria and plasmids

For the purposes of comparing the three models, target organisms were selected under the appropriate conditions for circular chromosomes, *dif* sites, Ter/Tus complexes and genomic compositional asymmetry of the GC skew index (GCSIs)≥0.1 (except for several *E.coli* strains that scored slightly below 0.1). The GCSI quantifies the degree of GC skew from the compositional distance between the leading and lagging strands and the extent to which the GC skew graph shape conforms to a discrete sine curve obtained using the Fast Fourier Transform. A threshold of 0.1 is relatively strict for ascertaining the existence of compositional bias [55], [78], [79].

The Ter and *dif* sites were identified by a homology search using a Ter consensus sequence and by a recursive hidden Markov modeling method, respectively [45]. In bacteria harboring a Tus protein and a replication terminus protein (RTP), 5′-AGNATGTTGTAAYKAA-3′ (allows mutations at 1, 4 and 16 bases; [12]) and 5′-KMACTAANWNNWCTATGTACYAAATNTTC- 3′ [13] were used as the Ter consensus sequence. For the set of plasmids used to derive distribution parameters for the fork-collision model, plasmids must have been replicated bi-directionally. Therefore the plasmid with theta replication machinery were selected according to the following criteria: 1) they must be larger than 10 Kbp with sufficient GCSI (window size: 64, spectral amplitude ≥1000; [79]), 2) they must contain neither Ter nor *dif* sites, 3) they must lack the *repC* gene, which is essential for rolling circle replication [80], and 4) no iteron sequences [81] are located near 5% region from putative replication origin predicted by GC skew shift point.

### Simulation of GC skew formation

The simulation of GC skew formation involves the following steps: 1) shuffling the genome sequence to create an unbiased initial sequence for simulation, while maintaining the same nucleotide composition, 2-a) definition of the leading and lagging strands based on a replication termination model and the position of the replication origin, 2-b) mutation of one random C to a G in the leading strand, 2-c) repeating from 2-a until the maximum simulation cycle is reached, and 3) validation of the simulated GC skew by comparison with the original genome sequence. The shuffled initial sequence was generated with the “shuffleseq" function of the G-language GAE, which is based on the Fisher-Yates algorithm [82]. All simulations used the same randomized sequences in each organism to avoid errors associated with shuffling. The maximum simulation cycle number was determined by the absolute difference in GC content between the whole genome and the leading strand. The replication origin was defined using the “find_ori_ter" function of the G-language GAE, which is based on the cumulative GC skew [26] at 1-bp resolution. The similarities between the simulated and natural GC skews were calculated using the root mean square error (RMSE).

### Replication termination models

Four replication termination models were constructed: fork-collision, fork-trap, *dif*-stop, and a control model terminating at the GC skew shift point, as described in Eqs. 1–4. In these equations, *X_i_* represents the replication terminus in bacteria *i*. In the fork-collision model, the positions of fork collision were empirically determined to follow a Gaussian distribution based on observations of the GC skew shift points in plasmids that lack fork-trap machinery and *dif* sites. The mean of this distribution (*μ*) was a locus directly opposite the replication origin, and the variance was *σ^2^*. Both of these values were normalized by the genome size (Eq. 1). The termini in the fork-trap model were defined by the locations of Ter sites in each bacterium, {*t_1_, t_2_, t_3_, … t_n_*}, each weighted with certain probabilities (Eq. 2). The termini in the *dif*-stop and control models were represented by the constant positions of *dif* sites (*C_d_*) or GC skew shift points (*C_s_*) (Eq. 3,4).
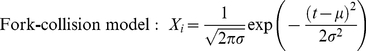
(1)

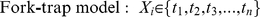
(2)

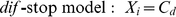
(3)

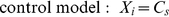
(4)


Assuming a simple model where all replication terminates with the fork-trap mechanism and where all replication forks progress continuously without stalling, replication should always terminate at a furthermost Ter site from the origin. In *E. coli*, this is TerC located at 1,607,184 bp, where position directly opposite from origin is at 1,603,784 bp and the *dif* site is at 1,588,773 bp. Second farthest Ter in the other replichore, namely TerA in the right replichore of *E. coli*, is only encountered if replication fork stalls a sufficient time (hereafter referred to as *δ*) in the right replichore for the replisome in the left replichore to over-travel to reach TerA. Since TerA is located 264,013 bp apart from a site directly opposite from the origin, and since the average speed of replisome is around 1,000 bp/s [2], *δ* in *E. coli* is calculated to be around 5 min. This is in accord with *in vivo* and *in vitro* findings, that stalling by supercoiling tension, protein blocking, and replisome assembly requires around 4–6 min to restart [83], [84], [85]. Such long stalling is known to occur *in vivo* in around 20% of replication events [73]. Stalling event should randomly and thus evenly occur in each replichore, and therefore, in *E. coli*, furthermost TerC is first encountered in 80% (without long replisome stalling)+10% (long replisome stalling in the same replichore), and TerA is first encountered in the remaining 10% of replication events. Furthermore, we considered the “leakiness" rate of each Ter site, which is approximately 80% as observed *in vivo* [86]. As a result, in *E. coli*, given the farthest inverted Ter sites from replication origin are TerC and TerA, followed by TerB, TerD, TerE and etc, pausing rate at each Ter site is TerC = 72%, TerA = 10.5%, TerB = 16%, TerD = 1.152%, TerE = 0.230%. The probability of having long enough stalling time *δ* so that the second furthest Ter site is utilized (20% in *E. coli*), is different in other species, due to the different distances of second farthest Ter sites from the region directly opposite of the origin. Assuming normal distribution of fork stall durations, this probability is calculated using *δ* of each species.

To validate these pausing rates, we further determined the optimized pausing ratio that best reconstructs the natural GC skew, by means of parameter tuning. For this parameter tuning, the patterns of the fork arrest ratios in each bacterium were tested in 5% increments, but since the comprehensive parameter searching in a bacterium harboring 10 Ter sites requires the calculation of 10,015,005 patterns and is not computationally realistic, the calculated combinations were limited to those having a sum total of fork arrest rates over 80%, with four Ter sites located farthest from the origin, based on *in vivo* observations [51]. As a result, the pausing rates calculated based on the stalling rates and Ter leakiness were very similar with the optimized pausing rates (*R* = 0.725, Spearman rank-correlation coefficient; Figure S8).

## Supporting Information

Figure S1 **Example of GC skew reconstruction simulation.** These figures are simulated GC skews when the simulated cycles (*t*) were 0, 10000, 15000, 20000, 25000 and 39120 (the maximum simulated cycle in *E. coli*). The GCSIs and RMSEs were described in the upper left of each graph. When the simulated cycle reaches 39120 (the bottom-right corner), red line (simulated GC skew) and green line (natural *E. coli* GC skew) almost completely overlap.
(PDF)

Figure S2 **Probabilistic error rates.** These figures show the probabilistic simulation error rates in 1000 iterations. Each error bar represents the standard deviation, with negligible average ≤0.0256.
(PDF)

Figure S3 **Simulation results in all target organisms.** The GC skew simulation results of the overall shapes and close-up around the shift-points in all target organisms are shown. The left figures show the overall view of GC skew, and the regions surrounded by dashed lines around the shift-point are extended as the right figures. In the right figures, red, green, blue and purple lines represent the natural GC skew, fork-trap model, fork- collision model and *dif-stop* model, respectively.
(PDF)

Figure S4 **Simulation results with three termination models and combination models in all target organisms.** Simulated GC skew graphs are shown, for the bacterial natural GC skew (red), fork-collision model (blue), fork-trap model (green), *dif-stop* model (purple), and probabilistic combination (light blue).
(PDF)

Figure S5 **Boxplot of RMSE of all simulated models.** The x-axis represents the models (*dif*-stop, fork-collision, fork-trap, shift-stop (control) models as well as probabilistic combination) and the y-axis represents the RMSE values. ** *p*<0.001, Wilcoxon test.
(PDF)

Figure S6 **Boxplot of RMSE of simulated models in Firmicutes.** The conceptual schemes and heat maps of RMSE scores for probabilistic combination (A) of replication termination models. (B) The x-axis represents the models (*dif*-stop, fork-collision, and probabilistic combinations) and the y-axis represents the RMSE values. ** *p*<0.001.
(PDF)

Figure S7 **Validation of simulations using only the third codon positions and non-coding sequences.** This figure shows the boxplot of the RMSE scores for the three replication termination models, representing the similarities between simulated and natural GC skews (in 65 bacteria). In comparison to Figure 3, here the GC skews were calculated and simulated only in the third codon positions and non-coding regions. The overall tendencies are identical to Figure 3. * *p*<0.05, ** *p*<0.01, Wilcoxon test.
(PDF)

Figure S8 **The replication fork pausing rates.** The x-axes represent the genome positions and the y-axes represents the percentages of pausing rates. In each bacterium, these pausing rates, which were calculated based on the experimental evidences, are very similar with the optimized pausing rates (*R* = 0.725, Spearman rank-correlation coefficient).
(PDF)
