# Supplementary figures and images for: Validation of Bacterial Replication Termination Models Using Simulation of Genomic Mutations
Source: PLoS One. 2012 Apr 3;7(4):e34526. doi: 10.1371/journal.pone.0034526 (PMC3317982; doi:10.1371/journal.pone.0034526)

GC skew

$t = 0$   
GCSI = 0.007, RMSE = 6.982

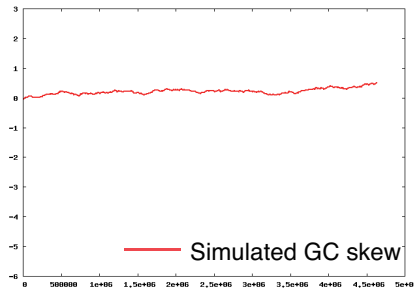

$t = 10,000$   
GCSI = 0.029, RMSE = 3.878

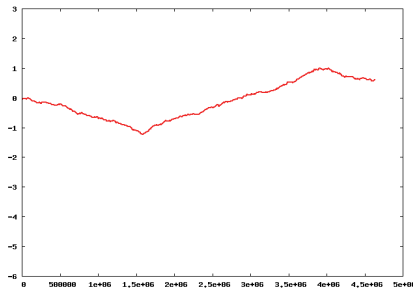

$t = 15,000$   
GCSI = 0.041, RMSE = 2.693

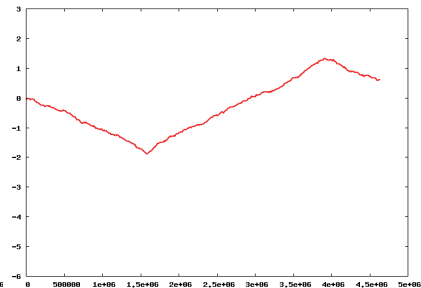

$t = 20,000$   
GCSI = 0.053, RMSE = 1.692

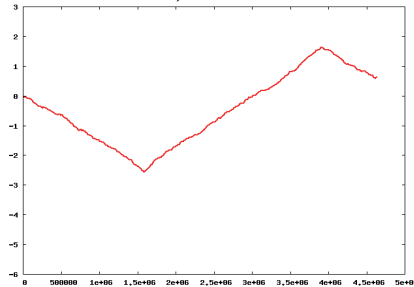

$t = 25,000$   
GCSI = 0.065, RMSE = 0.943

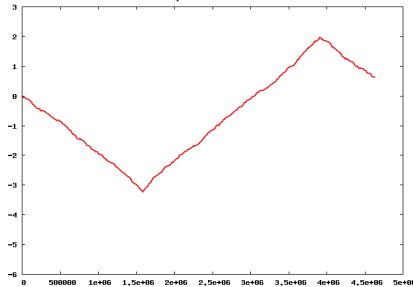

$t = 39,120$   
GCSI = 0.098, RMSE = 0.025

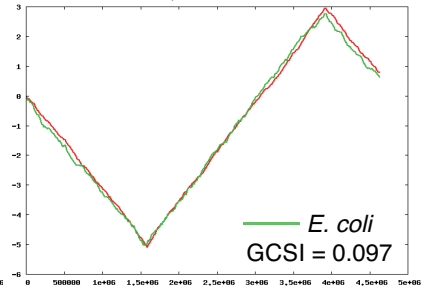

Genome position (bp)

Supplement: Figure S1 — Example of GC skew reconstruction simulation. These figures are simulated GC skews when the simulated cycles (t) were 0, 10000, 15000, 20000, 25000 and 39120 (the maximum simulated cycle in E. coli). The GCSIs and RMSEs were described in the upper left of each graph. When the simulated cycle reaches 39120 (the bottom-right corner), red line (simulated GC skew) and green line (natural E. coli GC skew) almost completely overlap. (PDF) [file pone.0034526.s001.pdf]

GC skew

Fork-collision model

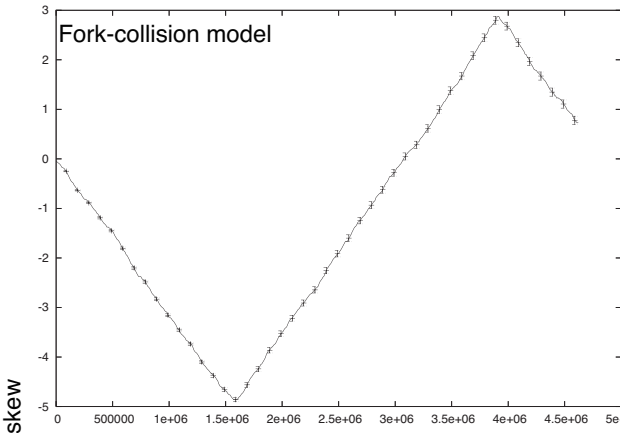

Fork-trap model

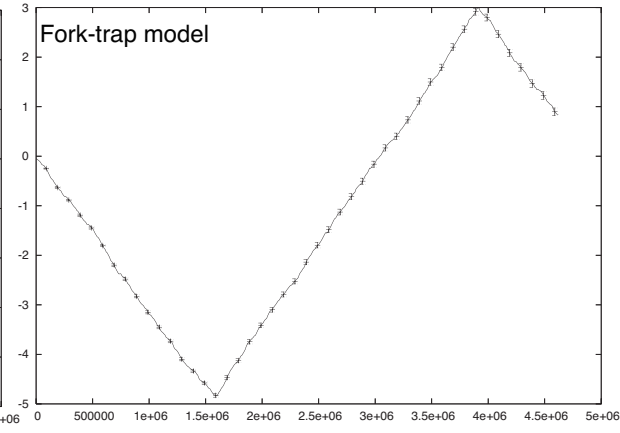*dif*-stop model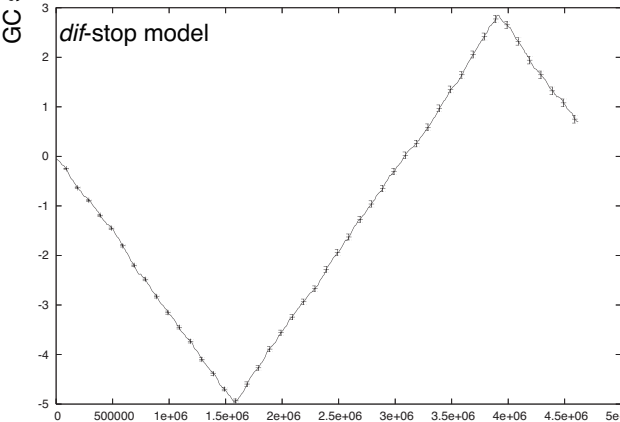

shift-stop model

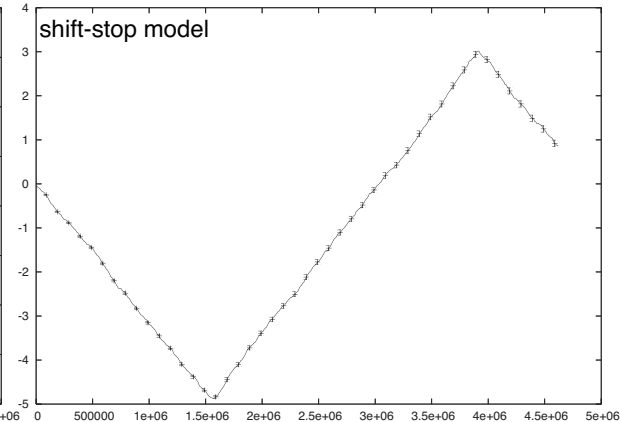

Genome position (bp)

Supplement: Figure S2 — Probabilistic error rates. These figures show the probabilistic simulation error rates in 1000 iterations. Each error bar represents the standard deviation, with negligible average ≤0.0256. (PDF) [file pone.0034526.s002.pdf]

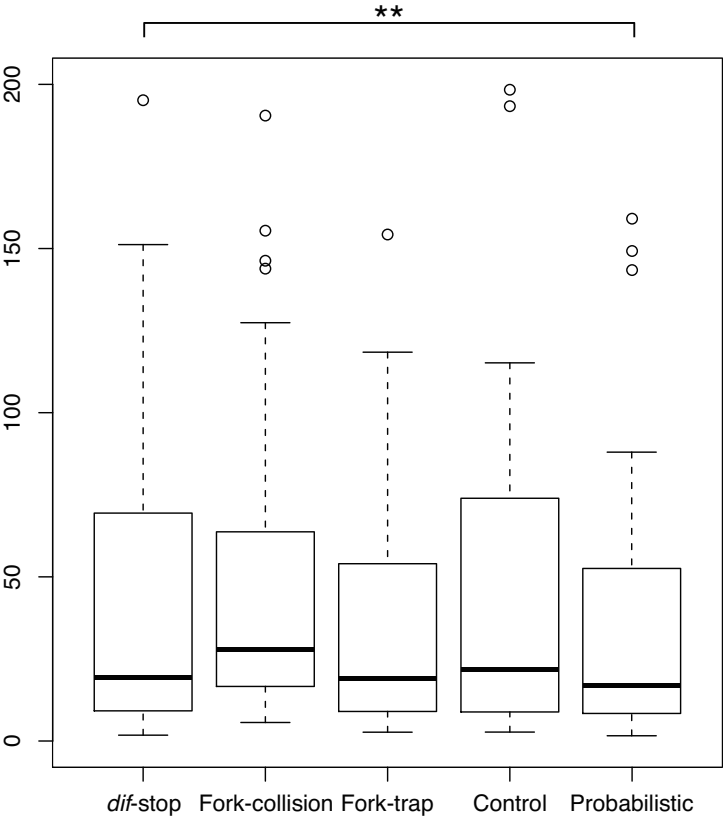

Supplement: Figure S5 — Boxplot of RMSE of all simulated models. The x-axis represents the models (dif-stop, fork-collision, fork-trap, shift-stop (control) models as well as probabilistic combination) and the y-axis represents the RMSE values. ** p<0.001, Wilcoxon test. (PDF) [file pone.0034526.s005.pdf]

# A

## Probabilistic combination

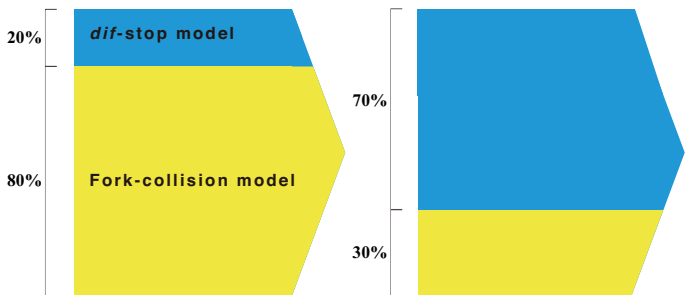

# B

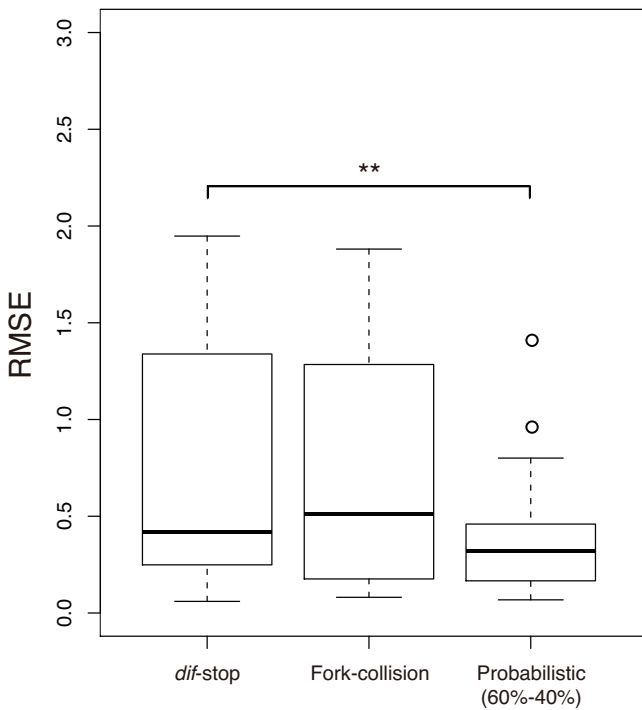

Supplement: Figure S6 — Boxplot of RMSE of simulated models in Firmicutes. The conceptual schemes and heat maps of RMSE scores for probabilistic combination (A) of replication termination models. (B) The x-axis represents the models (dif-stop, fork-collision, and probabilistic combinations) and the y-axis represents the RMSE values. ** p<0.001. (PDF) [file pone.0034526.s006.pdf]

# GC skew reproducibilities

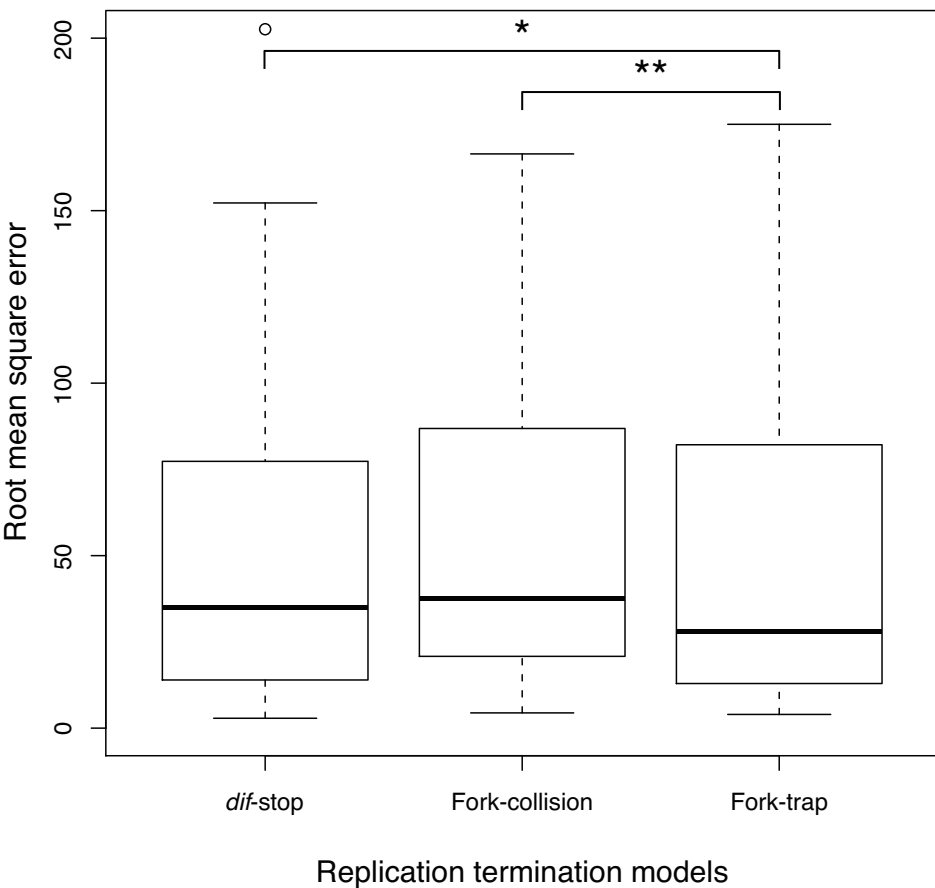

Supplement: Figure S7 — Validation of simulations using only the third codon positions and non-coding sequences. This figure shows the boxplot of the RMSE scores for the three replication termination models, representing the similarities between simulated and natural GC skews (in 65 bacteria). In comparison to Figure 3, here the GC skews were calculated and simulated only in the third codon positions and non-coding regions. The overall tendencies are identical to Figure 3. * p<0.05, ** p<0.01, Wilcoxon test. (PDF) [file pone.0034526.s007.pdf]
