# Supplementary material for: Validation of Bacterial Replication Termination Models Using Simulation of Genomic Mutations
Source: PLoS One. 2012 Apr 3;7(4):e34526. doi: 10.1371/journal.pone.0034526 (PMC3317982; doi:10.1371/journal.pone.0034526)

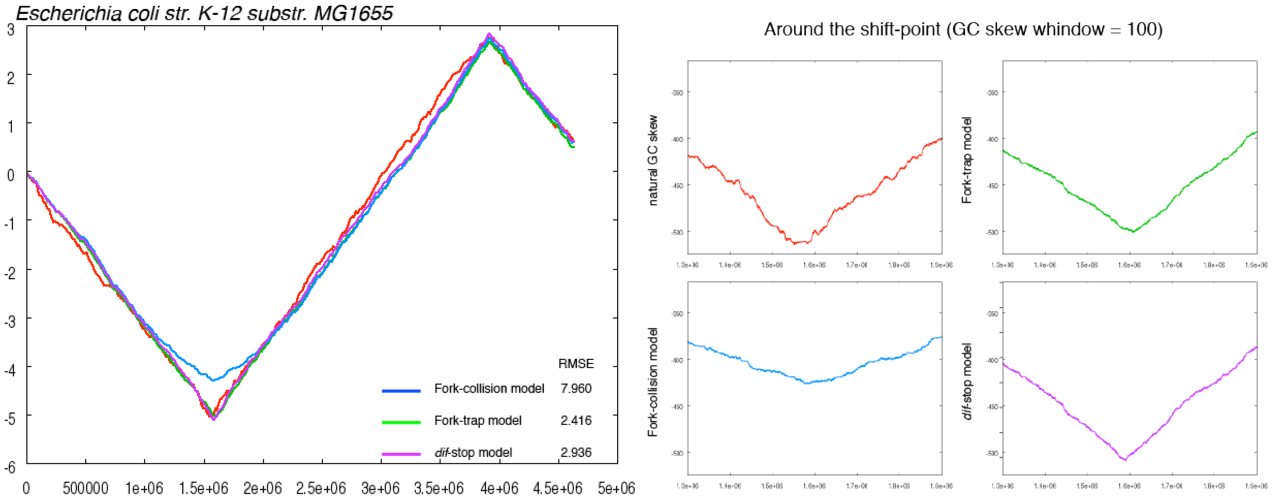
Escherichia coli str. K-12 substr. MG1655
RMSE
Fork-collision model 7.960
Fork-trap model 2.416
dif-stop model 2.936
Around the shift-point (GC skew whindow = 100)
natural GC skew
Fork-trap model
Fork-collision model
dif-stop model

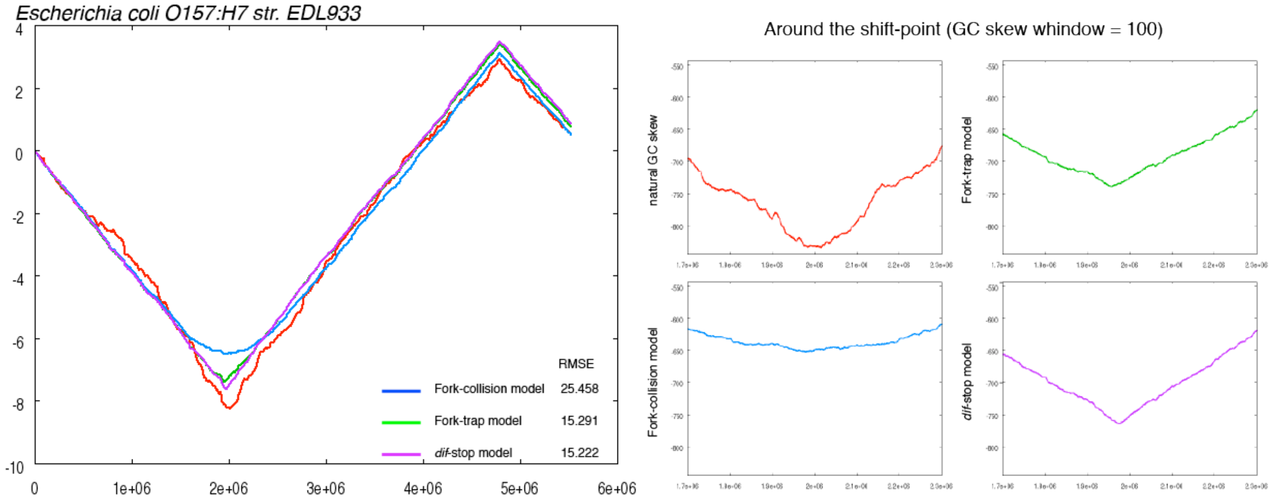
Escherichia coli O157:H7 str. EDL933
RMSE
Fork-collision model 25.458
Fork-trap model 15.291
dif-stop model 15.222
Around the shift-point (GC skew whindow = 100)
natural GC skew
Fork-trap model
Fork-collision model
dif-stop model

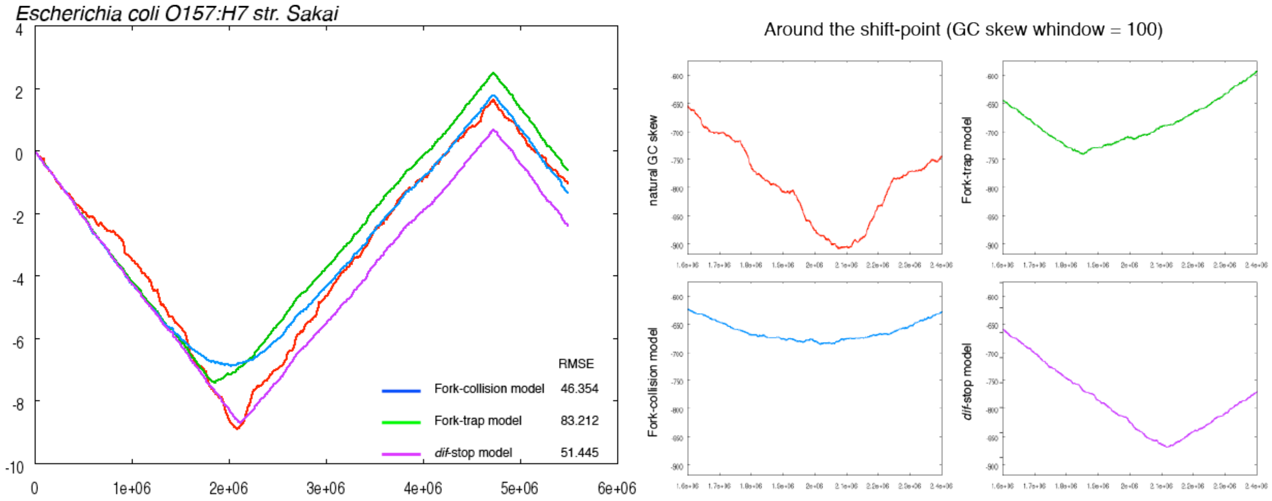
Escherichia coli O157:H7 str. Sakai
RMSE
Fork-collision model 46.354
Fork-trap model 83.212
dif-stop model 51.445
Around the shift-point (GC skew whindow = 100)
natural GC skew
Fork-trap model
Fork-collision model
dif-stop model

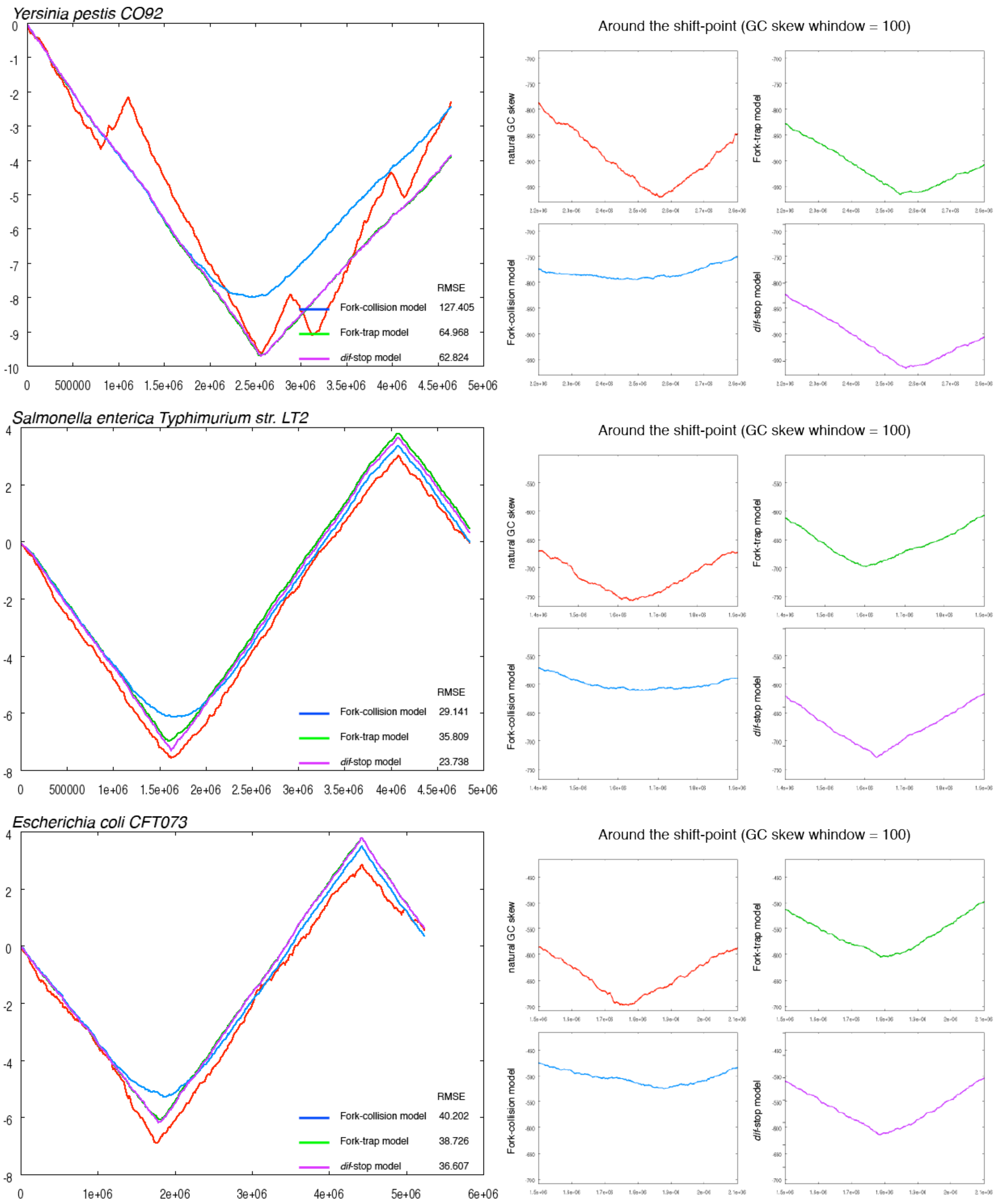

Yersinia pestis CO92
0
-1
-2
-3
-4
-5
-6
-7
-8
-9
-10
0
500000
1e+06
1.5e+06
2e+06
2.5e+06
3e+06
3.5e+06
4e+06
4.5e+06
5e+06
RMSE
Fork-collision model 127.405
Fork-trap model 64.968
dif-stop model 62.824
Around the shift-point (GC skew whindow = 100)
natural GC skew
Fork-trap model
Fork-collision model
dif-stop model
Salmonella enterica Typhimurium str. LT2
4
2
0
-2
-4
-6
-8
0
500000
1e+06
1.5e+06
2e+06
2.5e+06
3e+06
3.5e+06
4e+06
4.5e+06
5e+06
RMSE
Fork-collision model 29.141
Fork-trap model 35.809
dif-stop model 23.738
Around the shift-point (GC skew whindow = 100)
natural GC skew
Fork-trap model
Fork-collision model
dif-stop model
Escherichia coli CFT073
4
2
0
-2
-4
-6
-8
0
1e+06
2e+06
3e+06
4e+06
5e+06
6e+06
RMSE
Fork-collision model 40.202
Fork-trap model 38.726
dif-stop model 36.607
Around the shift-point (GC skew whindow = 100)
natural GC skew
Fork-trap model
Fork-collision model
dif-stop model

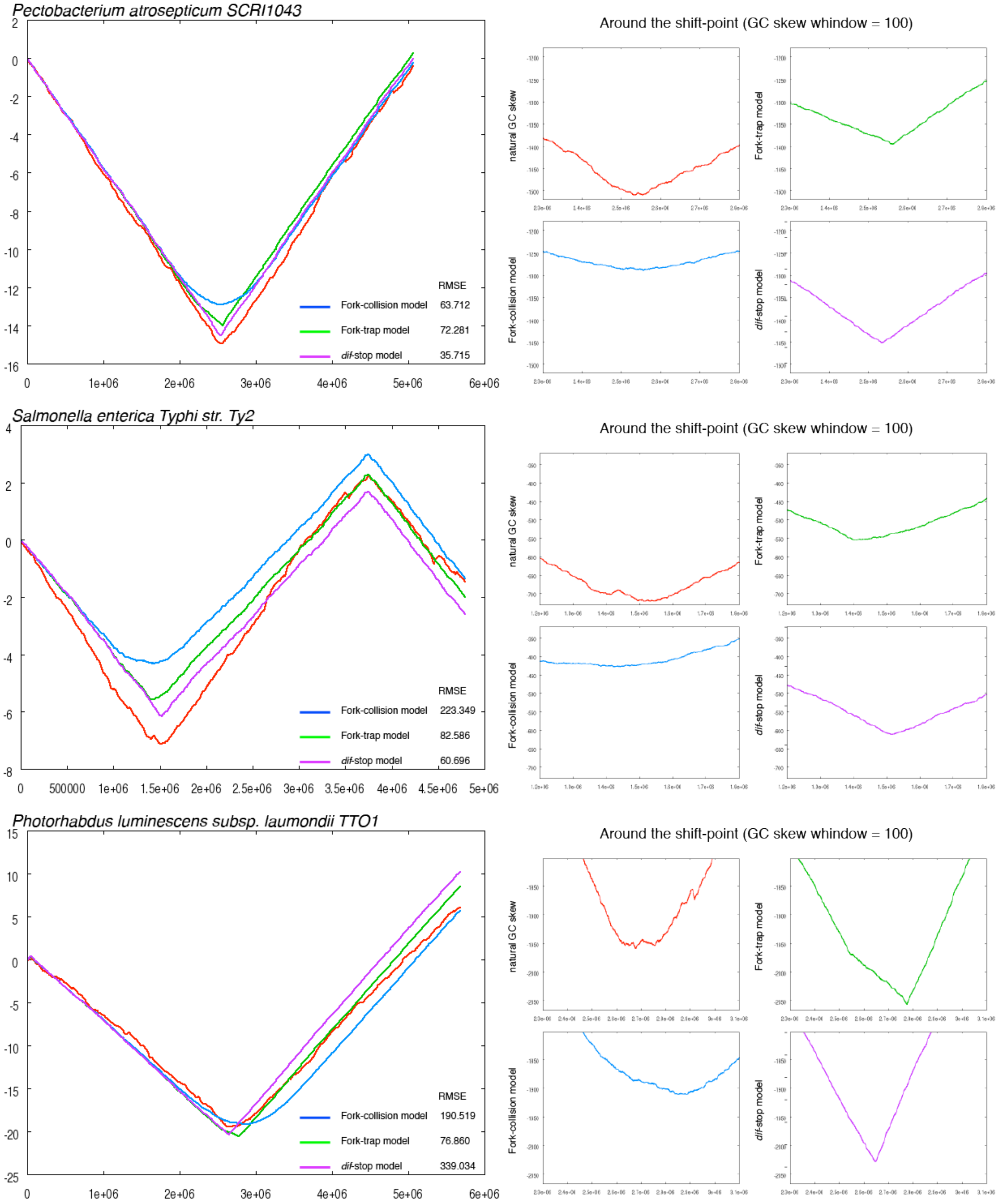

Pectobacterium atrosepticum SCRI1043
Around the shift-point (GC skew whindow = 100)
RMSE
Fork-collision model 63.712
Fork-trap model 72.281
dif-stop model 35.715
natural GC skew
Fork-trap model
Fork-collision model
dif-stop model
Salmonella enterica Typhi str. Ty2
Around the shift-point (GC skew whindow = 100)
RMSE
Fork-collision model 223.349
Fork-trap model 82.586
dif-stop model 60.696
natural GC skew
Fork-trap model
Fork-collision model
dif-stop model
Photorhabdus luminescens subsp. laumondii TTO1
Around the shift-point (GC skew whindow = 100)
RMSE
Fork-collision model 190.519
Fork-trap model 76.860
dif-stop model 339.034
natural GC skew
Fork-trap model
Fork-collision model
dif-stop model

Yersinia pseudotuberculosis IP 32953

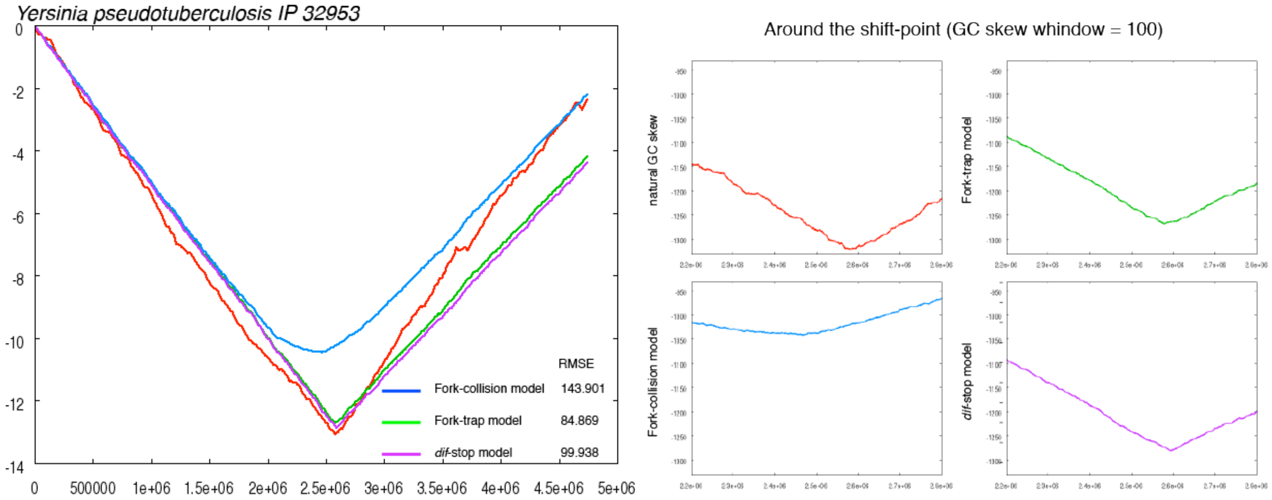

Salmonella enterica Paratyphi A str. ATCC 9150

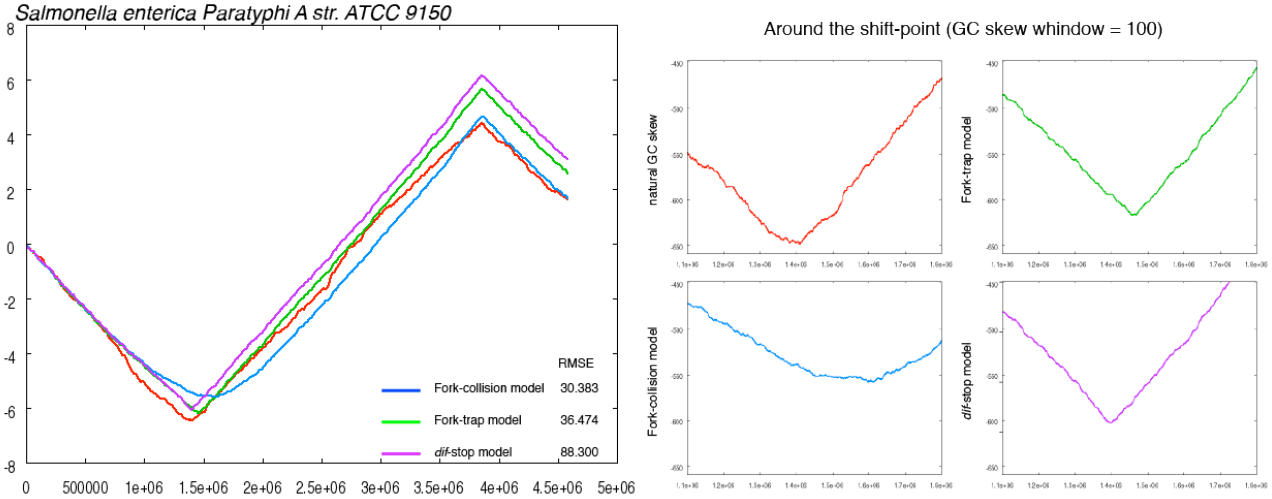

Salmonella enterica Choleraesuis str. SC-B67

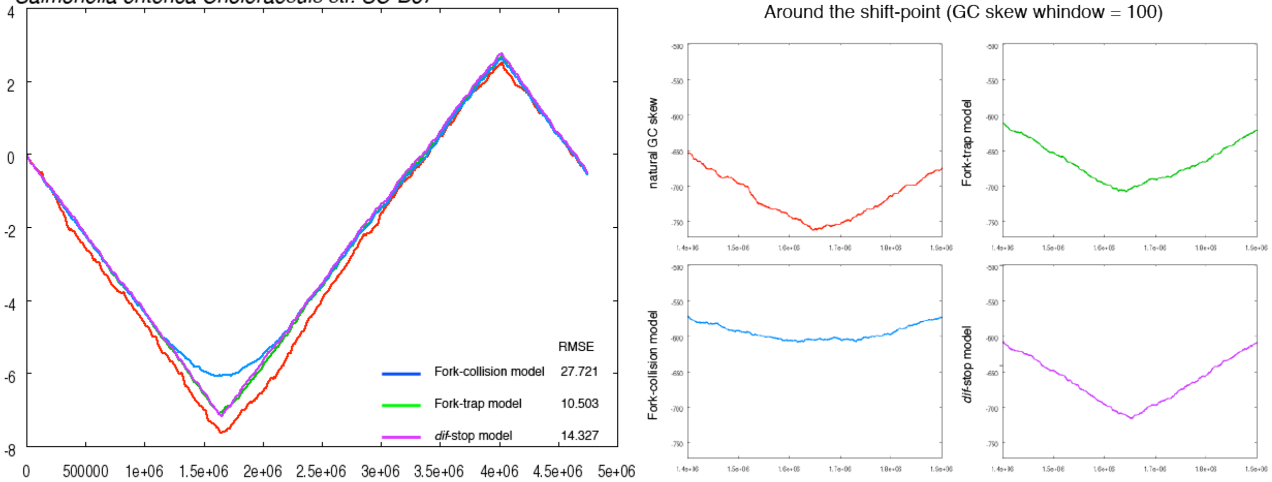

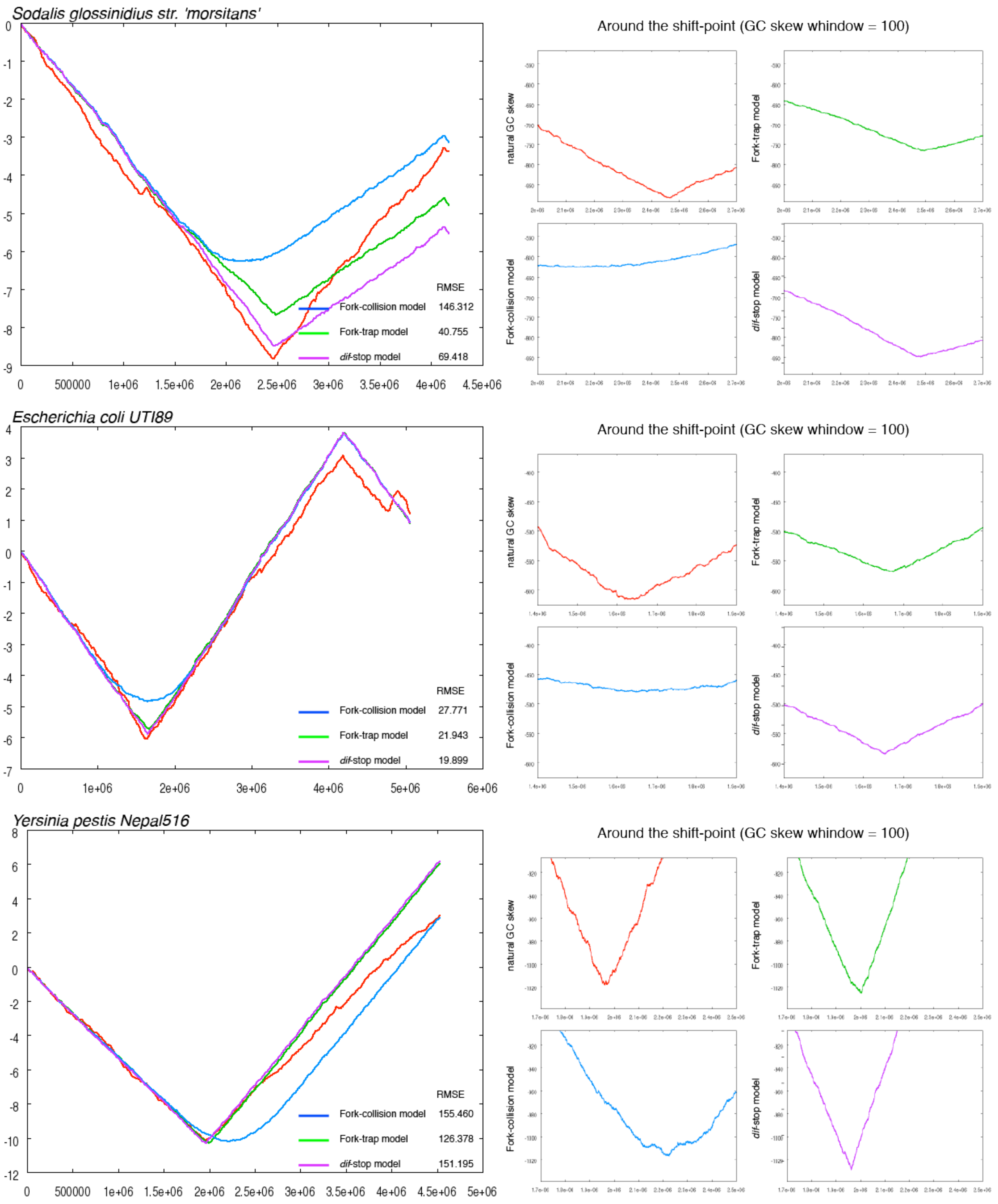

Sodalis glossinidius str. 'morsitans'
RMSE
Fork-collision model 146.312
Fork-trap model 40.755
dif-stop model 69.418
0 500000 1e+06 1.5e+06 2e+06 2.5e+06 3e+06 3.5e+06 4e+06 4.5e+06
Around the shift-point (GC skew whindow = 100)
natural GC skew
Fork-trap model
Fork-collision model
dif-stop model
Escherichia coli UTI89
RMSE
Fork-collision model 27.771
Fork-trap model 21.943
dif-stop model 19.899
0 1e+06 2e+06 3e+06 4e+06 5e+06 6e+06
Around the shift-point (GC skew whindow = 100)
natural GC skew
Fork-trap model
Fork-collision model
dif-stop model
Yersinia pestis Nepal516
RMSE
Fork-collision model 155.460
Fork-trap model 126.378
dif-stop model 151.195
0 500000 1e+06 1.5e+06 2e+06 2.5e+06 3e+06 3.5e+06 4e+06 4.5e+06 5e+06
Around the shift-point (GC skew whindow = 100)
natural GC skew
Fork-trap model
Fork-collision model
dif-stop model

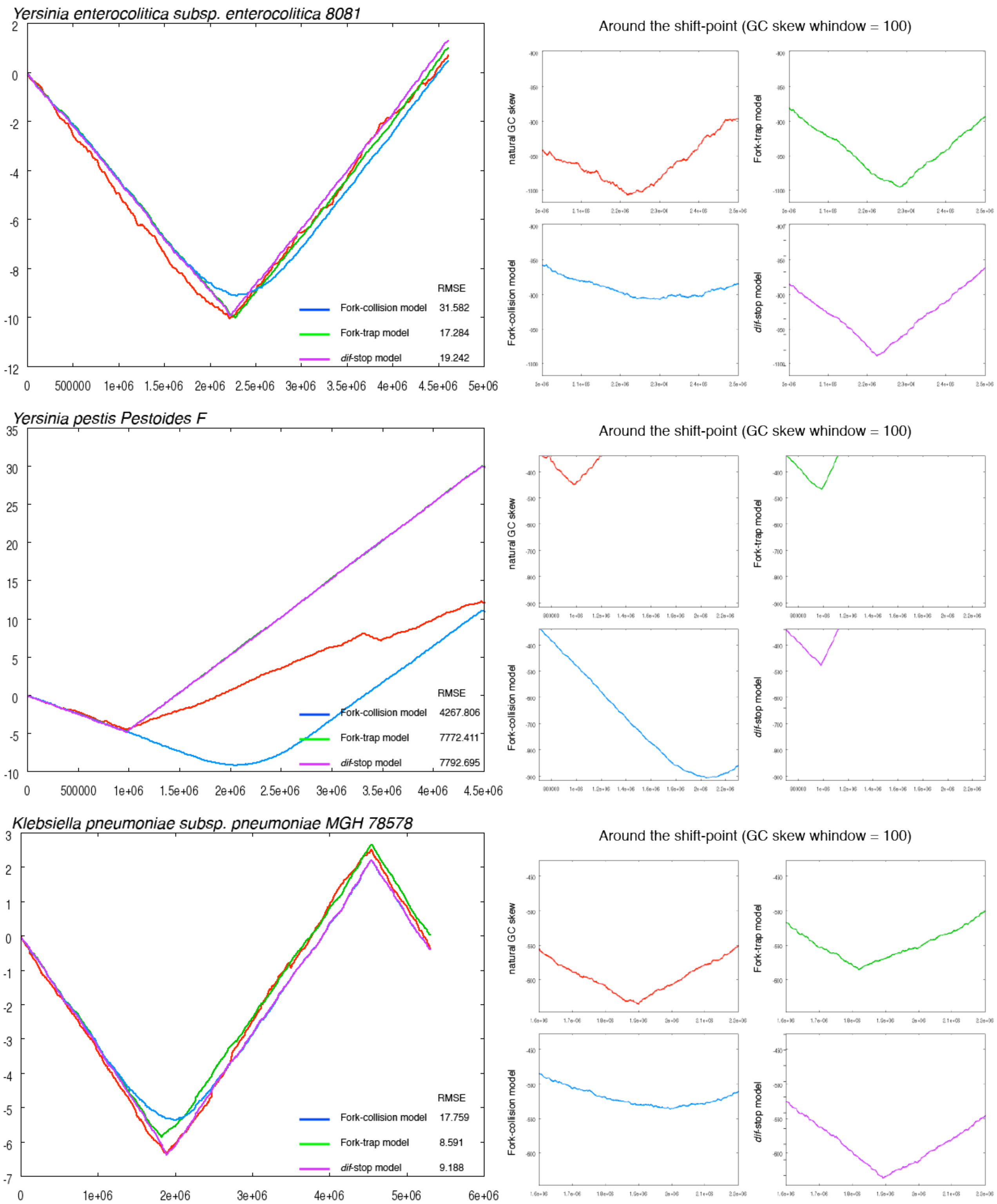

Yersinia enterocolitica subsp. enterocolitica 8081
RMSE
Fork-collision model 31.582
Fork-trap model 17.284
dif-stop model 19.242
Around the shift-point (GC skew whindow = 100)
natural GC skew
Fork-trap model
Fork-collision model
dif-stop model
Yersinia pestis Pestoides F
RMSE
Fork-collision model 4267.806
Fork-trap model 7772.411
dif-stop model 7792.695
Around the shift-point (GC skew whindow = 100)
natural GC skew
Fork-trap model
Fork-collision model
dif-stop model
Klebsiella pneumoniae subsp. pneumoniae MGH 78578
RMSE
Fork-collision model 17.759
Fork-trap model 8.591
dif-stop model 9.188
Around the shift-point (GC skew whindow = 100)
natural GC skew
Fork-trap model
Fork-collision model
dif-stop model

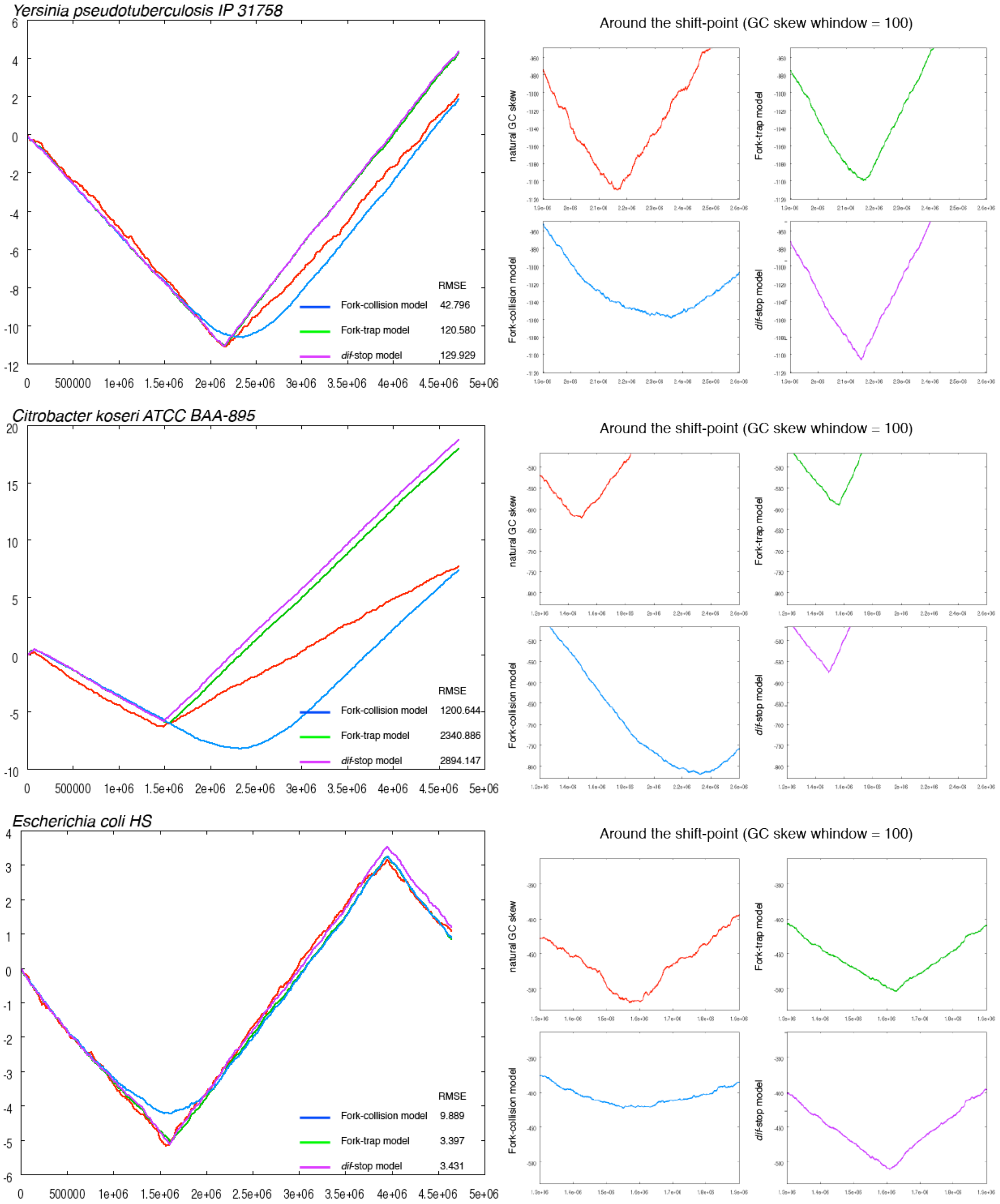

Yersinia pseudotuberculosis IP 31758
Around the shift-point (GC skew whindow = 100)
natural GC skew
Fork-trap model
Fork-collision model
dif-stop model
RMSE
Fork-collision model 42.796
Fork-trap model 120.580
dif-stop model 129.929
Citrobacter koseri ATCC BAA-895
Around the shift-point (GC skew whindow = 100)
natural GC skew
Fork-trap model
Fork-collision model
dif-stop model
RMSE
Fork-collision model 1200.644
Fork-trap model 2340.886
dif-stop model 2894.147
Escherichia coli HS
Around the shift-point (GC skew whindow = 100)
natural GC skew
Fork-trap model
Fork-collision model
dif-stop model
RMSE
Fork-collision model 9.889
Fork-trap model 3.397
dif-stop model 3.431

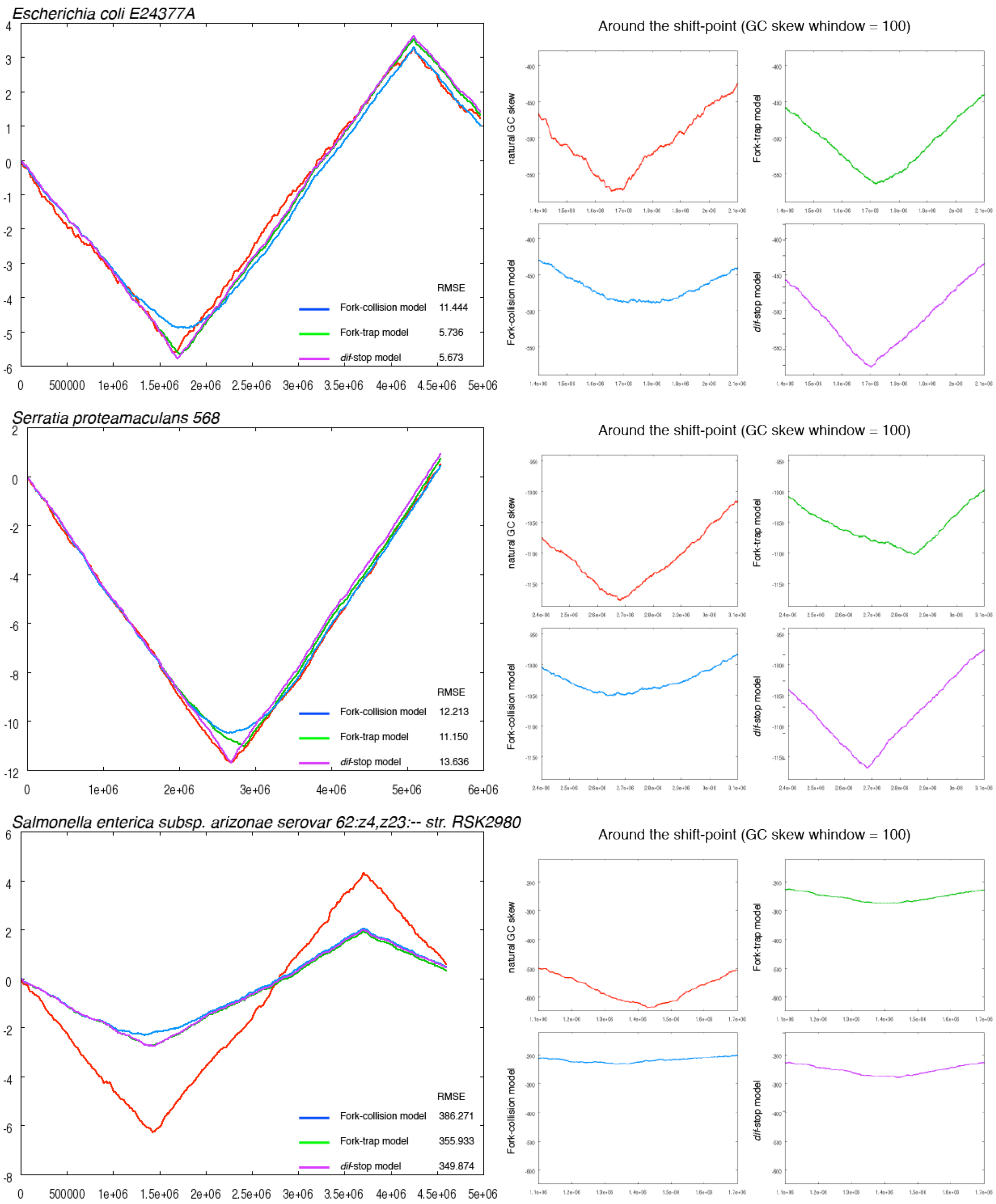

Escherichia coli E24377A
RMSE
Fork-collision model 11.444
Fork-trap model 5.736
dif-stop model 5.673
Around the shift-point (GC skew whindow = 100)
natural GC skew
Fork-trap model
Fork-collision model
dif-stop model
Serratia proteamaculans 568
RMSE
Fork-collision model 12.213
Fork-trap model 11.150
dif-stop model 13.636
Around the shift-point (GC skew whindow = 100)
natural GC skew
Fork-trap model
Fork-collision model
dif-stop model
Salmonella enterica subsp. arizonae serovar 62:z4,z23:-- str. RSK2980
RMSE
Fork-collision model 386.271
Fork-trap model 355.933
dif-stop model 349.874
Around the shift-point (GC skew whindow = 100)
natural GC skew
Fork-trap model
Fork-collision model
dif-stop model

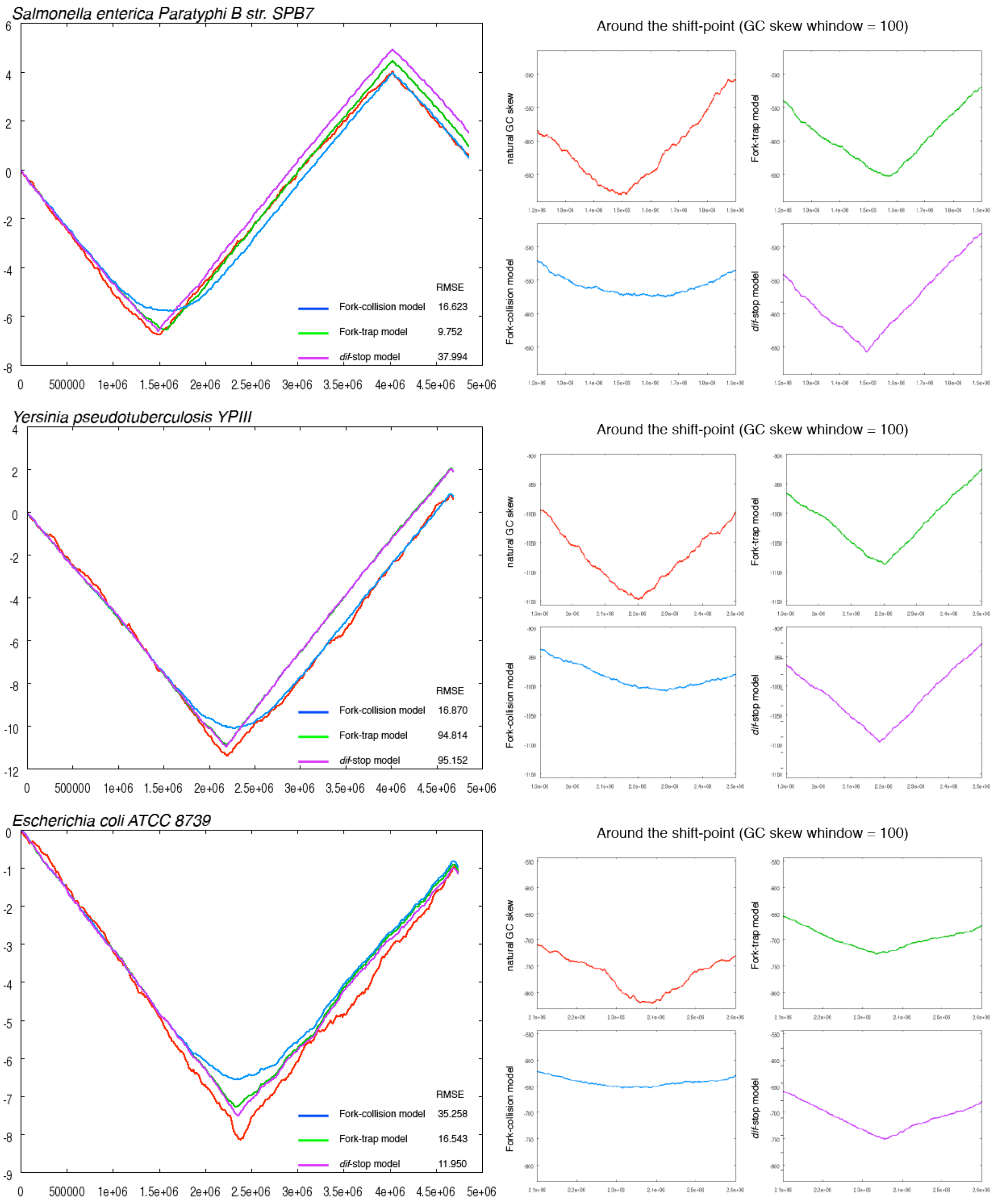

Salmonella enterica Paratyphi B str. SPB7
Around the shift-point (GC skew whindow = 100)
RMSE
Fork-collision model 16.623
Fork-trap model 9.752
dif-stop model 37.994
natural GC skew
Fork-trap model
Fork-collision model
dif-stop model
Yersinia pseudotuberculosis YPIII
Around the shift-point (GC skew whindow = 100)
RMSE
Fork-collision model 16.870
Fork-trap model 94.814
dif-stop model 95.152
natural GC skew
Fork-trap model
Fork-collision model
dif-stop model
Escherichia coli ATCC 8739
Around the shift-point (GC skew whindow = 100)
RMSE
Fork-collision model 35.258
Fork-trap model 16.543
dif-stop model 11.950
natural GC skew
Fork-trap model
Fork-collision model
dif-stop model

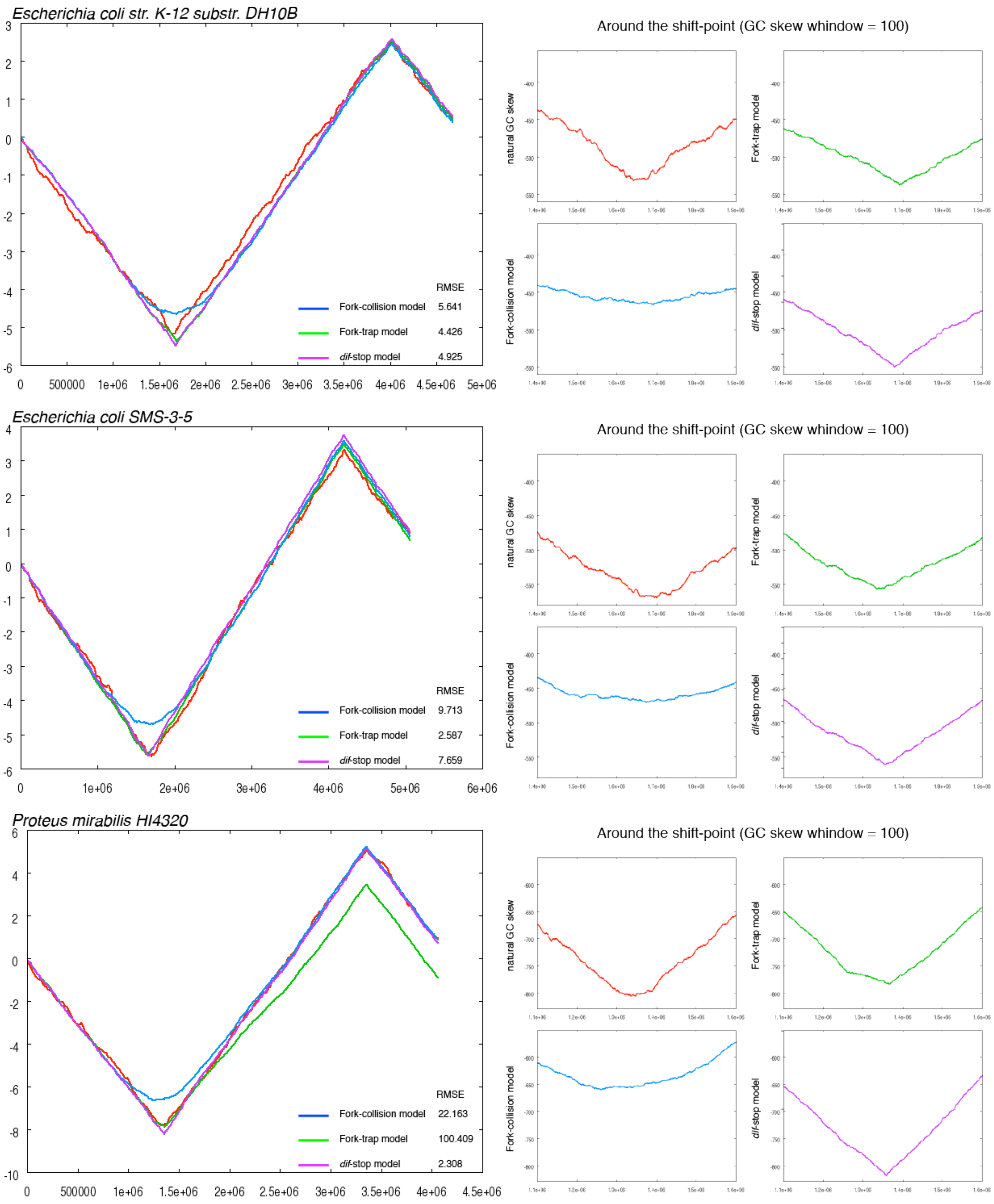
Escherichia coli str. K-12 substr. DH10B
RMSE
Fork-collision model 5.641
Fork-trap model 4.426
dif-stop model 4.925
0 500000 1e+06 1.5e+06 2e+06 2.5e+06 3e+06 3.5e+06 4e+06 4.5e+06 5e+06
Around the shift-point (GC skew whindow = 100)
natural GC skew
Fork-trap model
Fork-collision model
dif-stop model
Escherichia coli SMS-3-5
RMSE
Fork-collision model 9.713
Fork-trap model 2.587
dif-stop model 7.659
0 1e+06 2e+06 3e+06 4e+06 5e+06 6e+06
Around the shift-point (GC skew whindow = 100)
natural GC skew
Fork-trap model
Fork-collision model
dif-stop model
Proteus mirabilis HI4320
RMSE
Fork-collision model 22.163
Fork-trap model 100.409
dif-stop model 2.308
0 500000 1e+06 1.5e+06 2e+06 2.5e+06 3e+06 3.5e+06 4e+06 4.5e+06
Around the shift-point (GC skew whindow = 100)
natural GC skew
Fork-trap model
Fork-collision model
dif-stop model

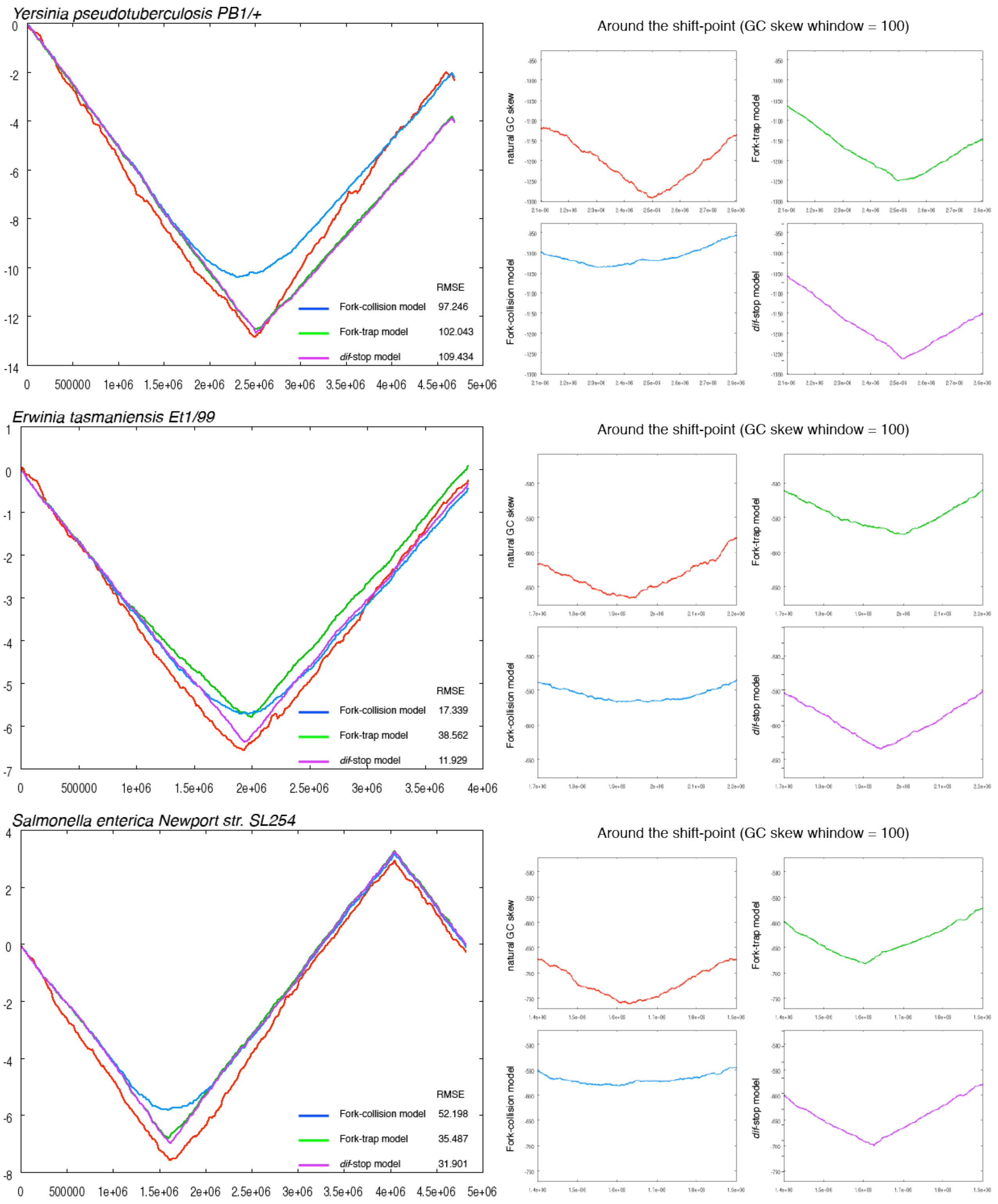
Yersinia pseudotuberculosis PB1/+
RMSE
Fork-collision model 97.246
Fork-trap model 102.043
dif-stop model 109.434
Around the shift-point (GC skew whindow = 100)
natural GC skew
Fork-trap model
Fork-collision model
dif-stop model
Erwinia tasmaniensis Et1/99
RMSE
Fork-collision model 17.339
Fork-trap model 38.562
dif-stop model 11.929
Around the shift-point (GC skew whindow = 100)
natural GC skew
Fork-trap model
Fork-collision model
dif-stop model
Salmonella enterica Newport str. SL254
RMSE
Fork-collision model 52.198
Fork-trap model 35.487
dif-stop model 31.901
Around the shift-point (GC skew whindow = 100)
natural GC skew
Fork-trap model
Fork-collision model
dif-stop model

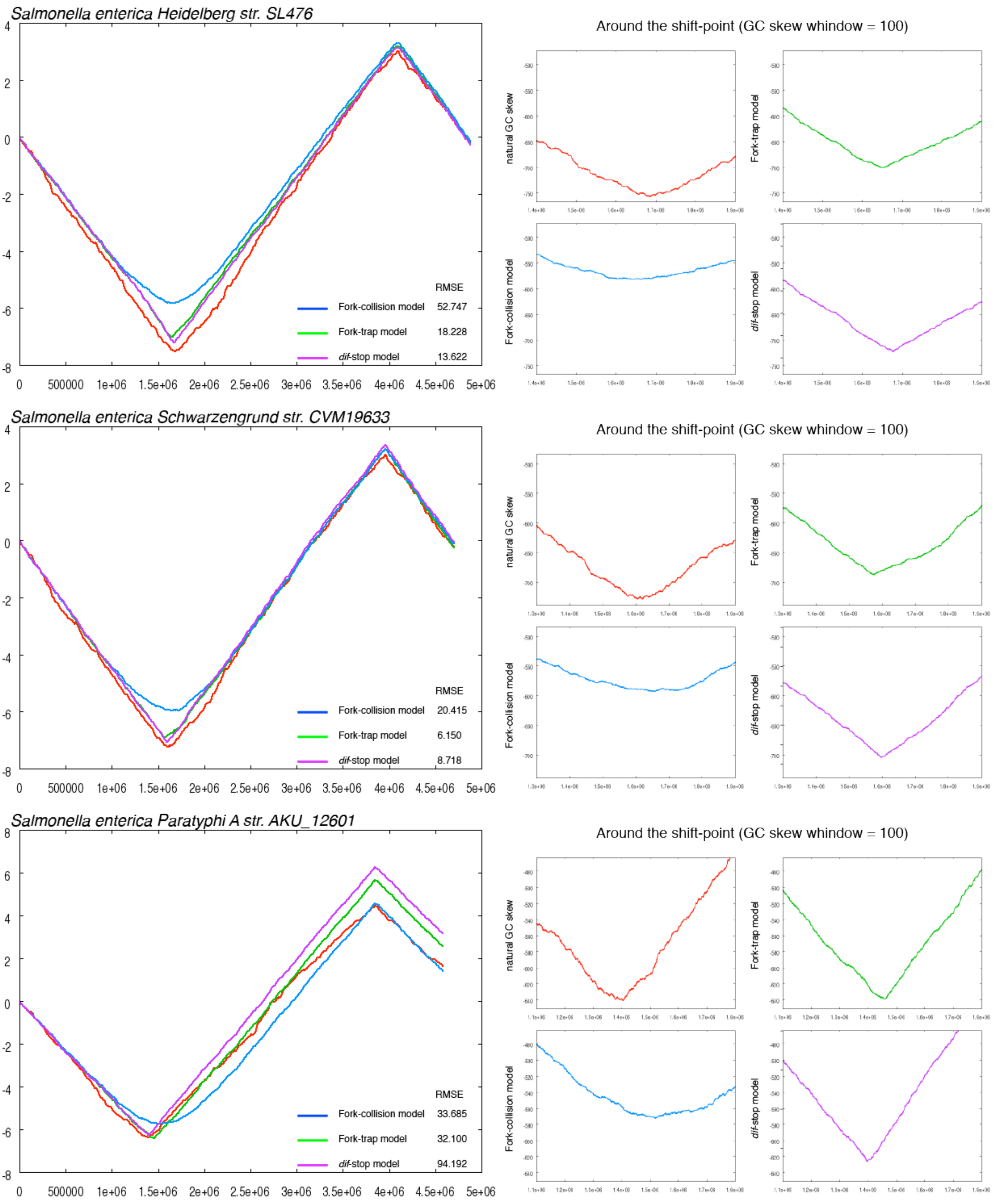

Salmonella enterica Heidelberg str. SL476
RMSE
Fork-collision model 52.747
Fork-trap model 18.228
dif-stop model 13.622
0
500000
1e+06
1.5e+06
2e+06
2.5e+06
3e+06
3.5e+06
4e+06
4.5e+06
5e+06
Around the shift-point (GC skew whindow = 100)
natural GC skew
Fork-trap model
Fork-collision model
dif-stop model
Salmonella enterica Schwarzengrund str. CVM19633
RMSE
Fork-collision model 20.415
Fork-trap model 6.150
dif-stop model 8.718
Around the shift-point (GC skew whindow = 100)
Salmonella enterica Paratyphi A str. AKU_12601
RMSE
Fork-collision model 33.685
Fork-trap model 32.100
dif-stop model 94.192
Around the shift-point (GC skew whindow = 100)

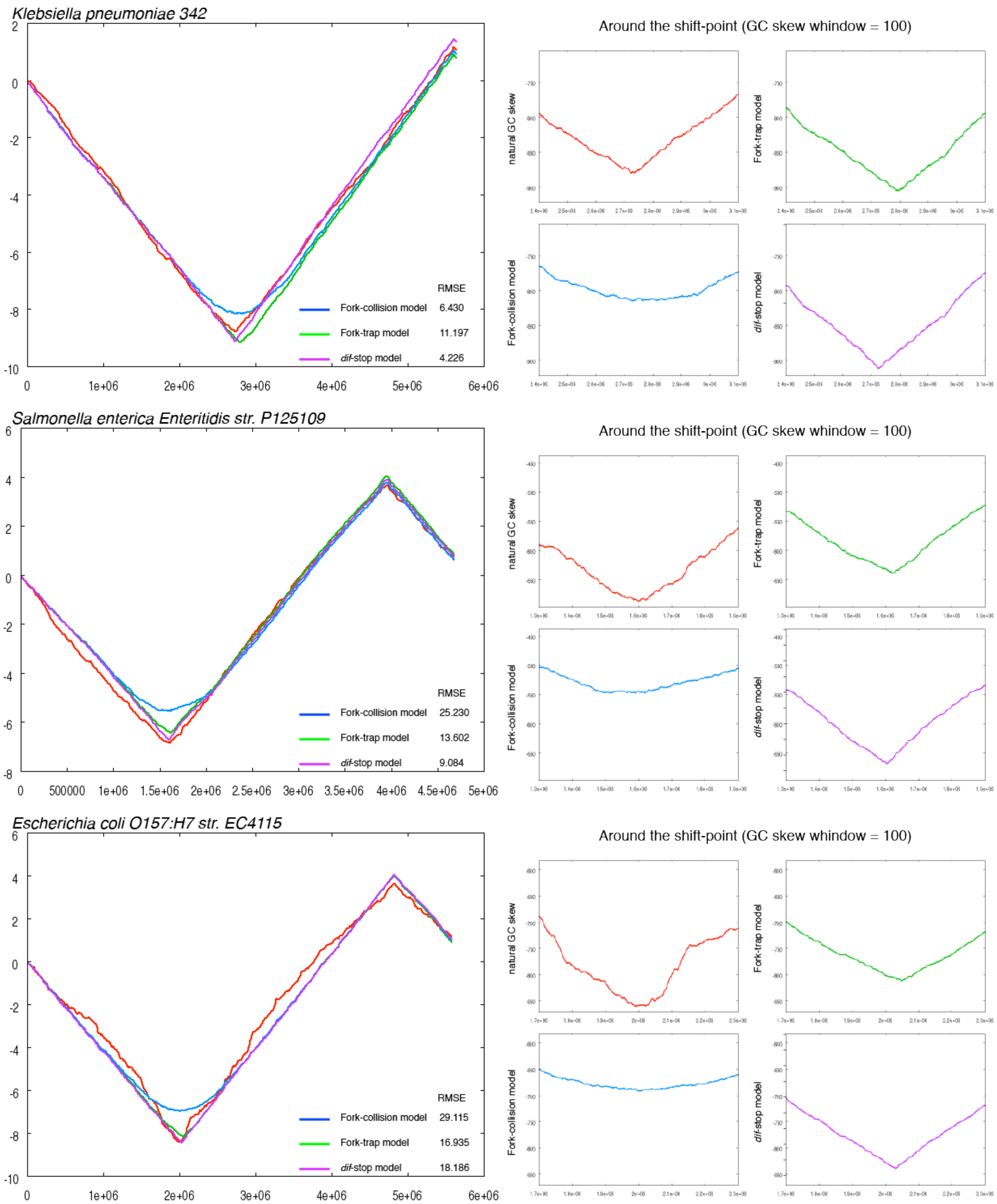
Klebsiella pneumoniae 342
RMSE
Fork-collision model 6.430
Fork-trap model 11.197
dif-stop model 4.226
Around the shift-point (GC skew whindow = 100)
natural GC skew
Fork-trap model
Fork-collision model
dif-stop model
Salmonella enterica Enteritidis str. P125109
RMSE
Fork-collision model 25.230
Fork-trap model 13.602
dif-stop model 9.084
Around the shift-point (GC skew whindow = 100)
natural GC skew
Fork-trap model
Fork-collision model
dif-stop model
Escherichia coli O157:H7 str. EC4115
RMSE
Fork-collision model 29.115
Fork-trap model 16.935
dif-stop model 18.186
Around the shift-point (GC skew whindow = 100)
natural GC skew
Fork-trap model
Fork-collision model
dif-stop model

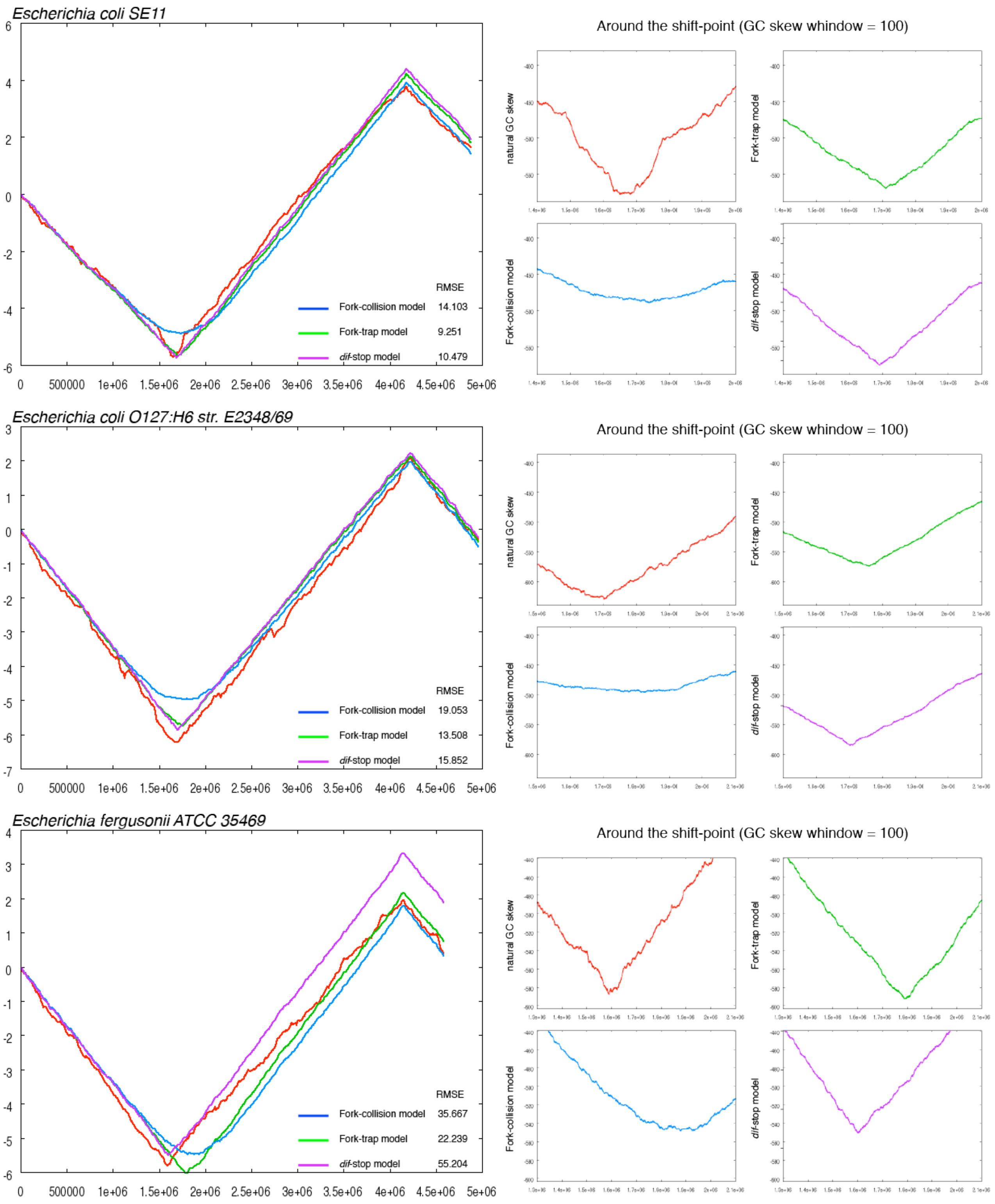

Escherichia coli SE11
Around the shift-point (GC skew whindow = 100)
RMSE
Fork-collision model 14.103
Fork-trap model 9.251
dif-stop model 10.479
natural GC skew
Fork-trap model
Fork-collision model
dif-stop model
Escherichia coli O127:H6 str. E2348/69
Around the shift-point (GC skew whindow = 100)
RMSE
Fork-collision model 19.053
Fork-trap model 13.508
dif-stop model 15.852
natural GC skew
Fork-trap model
Fork-collision model
dif-stop model
Escherichia fergusonii ATCC 35469
Around the shift-point (GC skew whindow = 100)
RMSE
Fork-collision model 35.667
Fork-trap model 22.239
dif-stop model 55.204
natural GC skew
Fork-trap model
Fork-collision model
dif-stop model

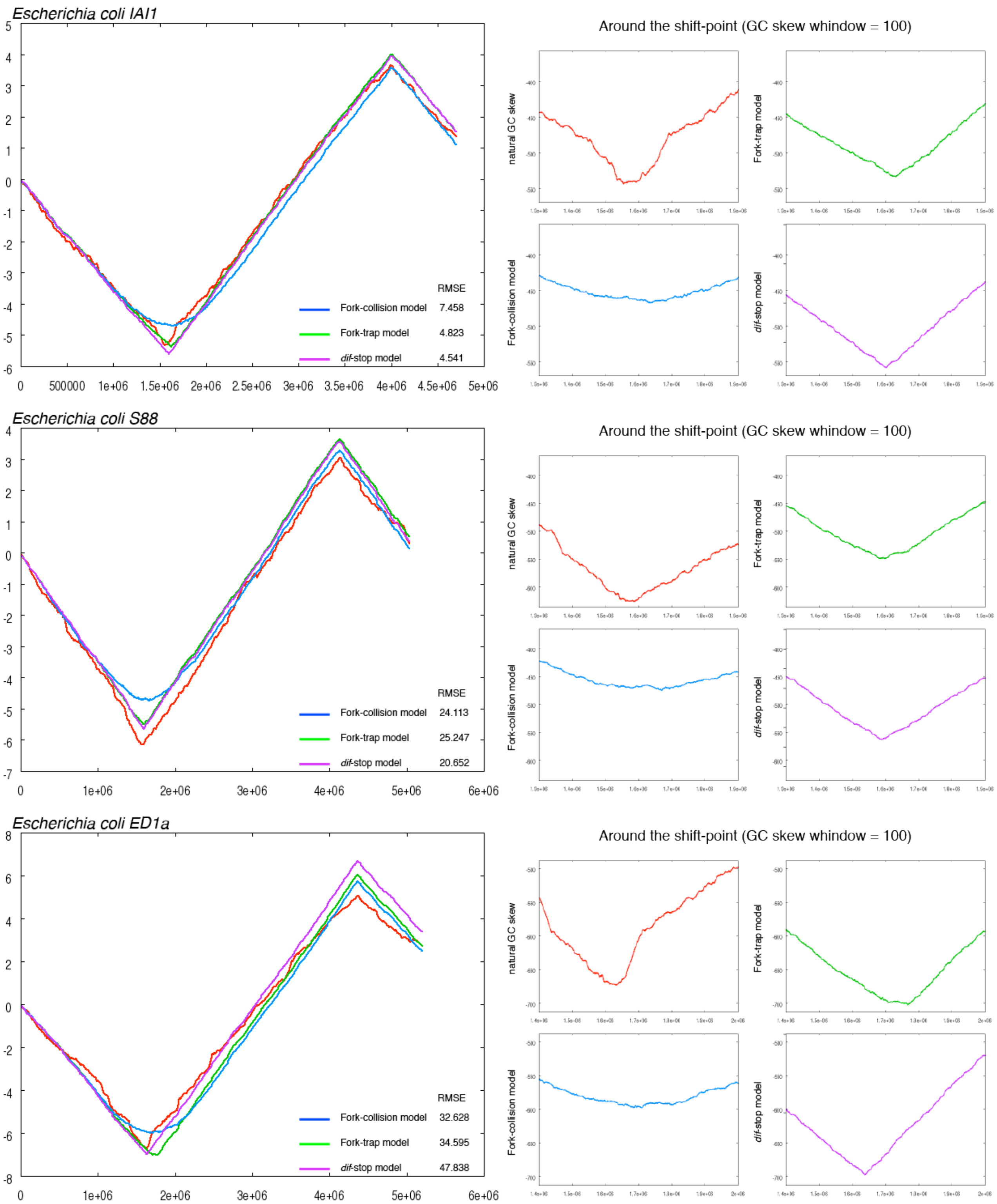
Escherichia coli IAI1
Around the shift-point (GC skew whindow = 100)
RMSE
Fork-collision model 7.458
Fork-trap model 4.823
dif-stop model 4.541
natural GC skew
Fork-trap model
Fork-collision model
dif-stop model
Escherichia coli S88
Around the shift-point (GC skew whindow = 100)
RMSE
Fork-collision model 24.113
Fork-trap model 25.247
dif-stop model 20.652
natural GC skew
Fork-trap model
Fork-collision model
dif-stop model
Escherichia coli ED1a
Around the shift-point (GC skew whindow = 100)
RMSE
Fork-collision model 32.628
Fork-trap model 34.595
dif-stop model 47.838
natural GC skew
Fork-trap model
Fork-collision model
dif-stop model

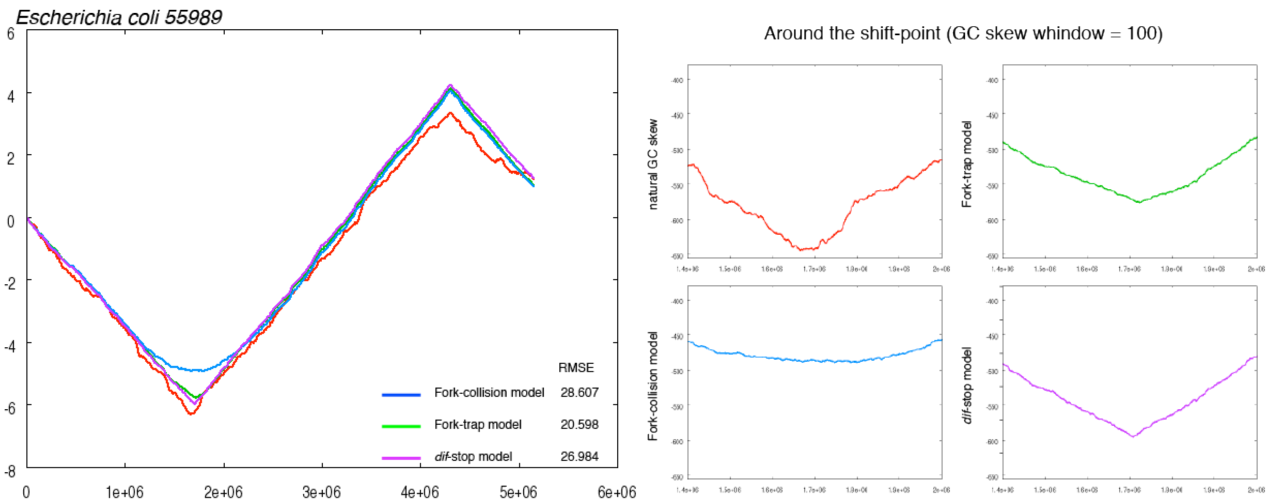

Escherichia coli 55989
Around the shift-point (GC skew whindow = 100)
RMSE
Fork-collision model 28.607
Fork-trap model 20.598
dif-stop model 26.984
natural GC skew
Fork-trap model
Fork-collision model
dif-stop model

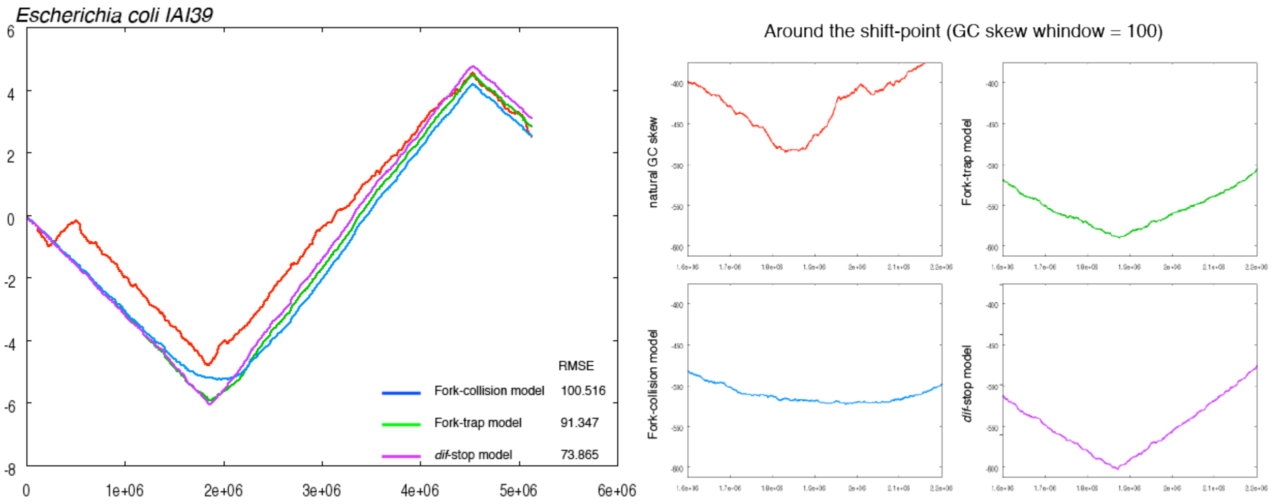

Escherichia coli IAI39
Around the shift-point (GC skew whindow = 100)
RMSE
Fork-collision model 100.516
Fork-trap model 91.347
dif-stop model 73.865
natural GC skew
Fork-trap model
Fork-collision model
dif-stop model

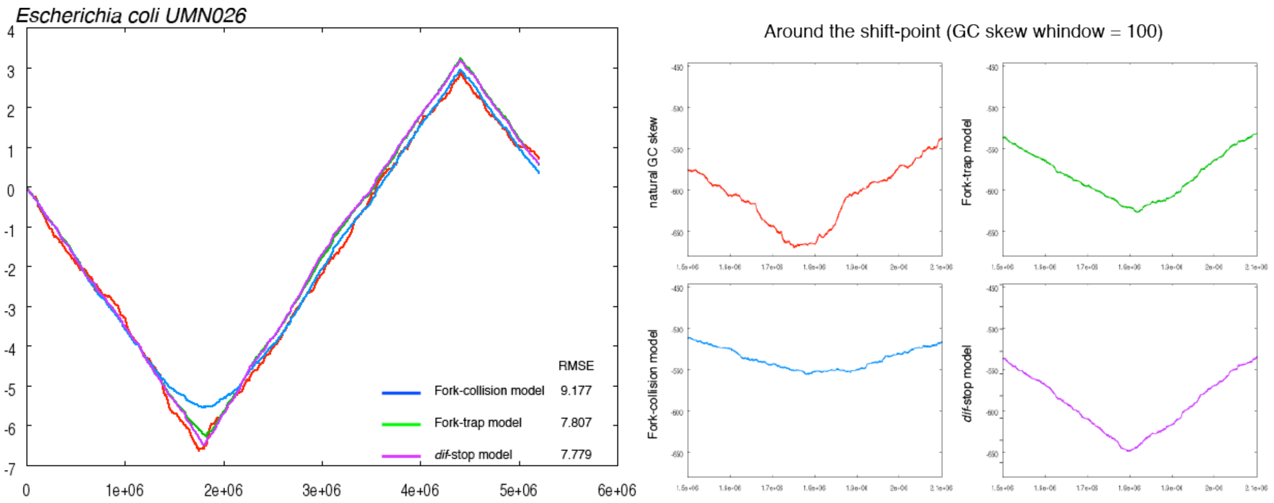

Escherichia coli UMN026
Around the shift-point (GC skew whindow = 100)
RMSE
Fork-collision model 9.177
Fork-trap model 7.807
dif-stop model 7.779
natural GC skew
Fork-trap model
Fork-collision model
dif-stop model

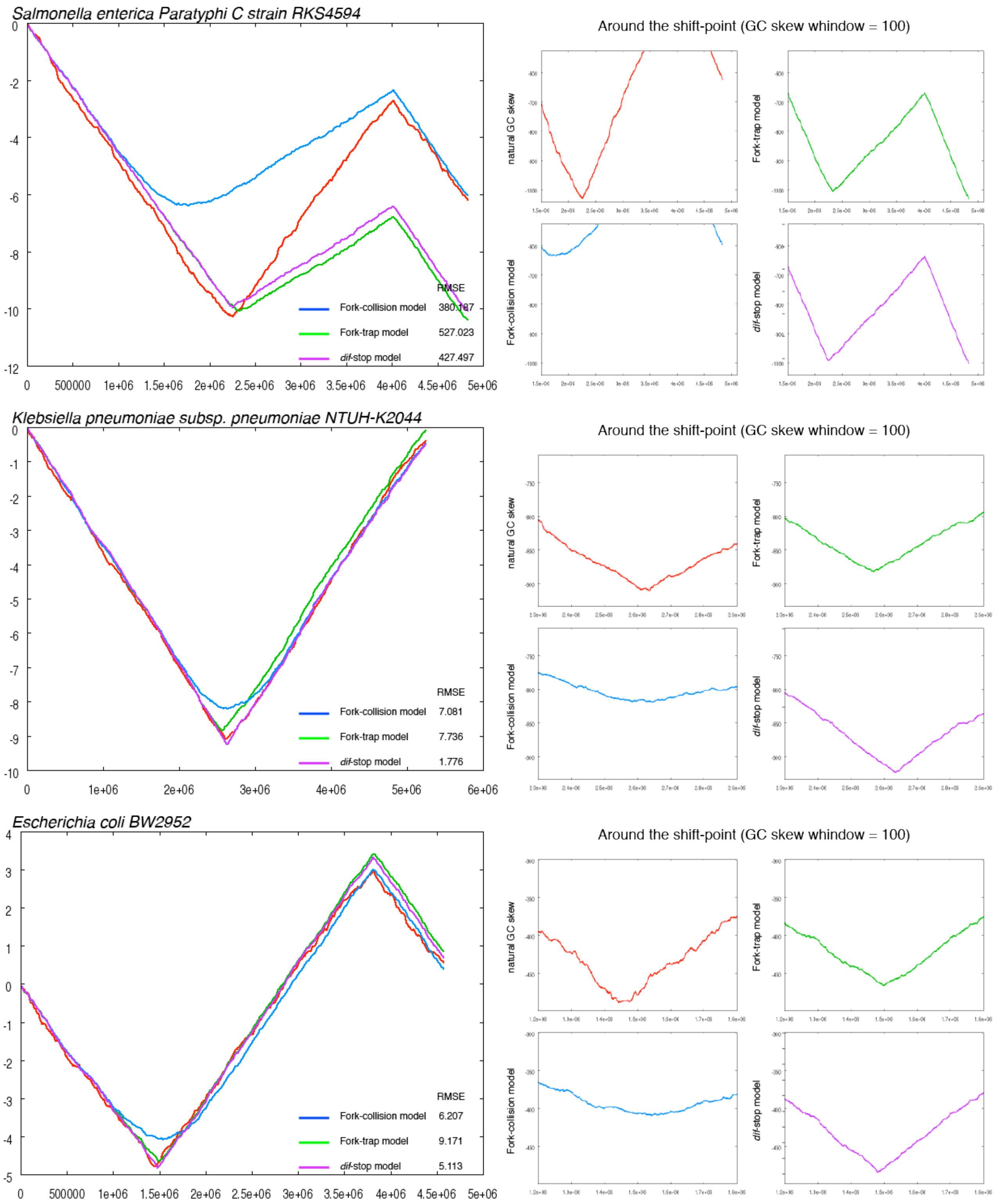

Salmonella enterica Paratyphi C strain RKS4594
RMSE
Fork-collision model 380.187
Fork-trap model 527.023
dif-stop model 427.497
Around the shift-point (GC skew whindow = 100)
natural GC skew
Fork-trap model
Fork-collision model
dif-stop model
Klebsiella pneumoniae subsp. pneumoniae NTUH-K2044
RMSE
Fork-collision model 7.081
Fork-trap model 7.736
dif-stop model 1.776
Around the shift-point (GC skew whindow = 100)
natural GC skew
Fork-trap model
Fork-collision model
dif-stop model
Escherichia coli BW2952
RMSE
Fork-collision model 6.207
Fork-trap model 9.171
dif-stop model 5.113
Around the shift-point (GC skew whindow = 100)
natural GC skew
Fork-trap model
Fork-collision model
dif-stop model

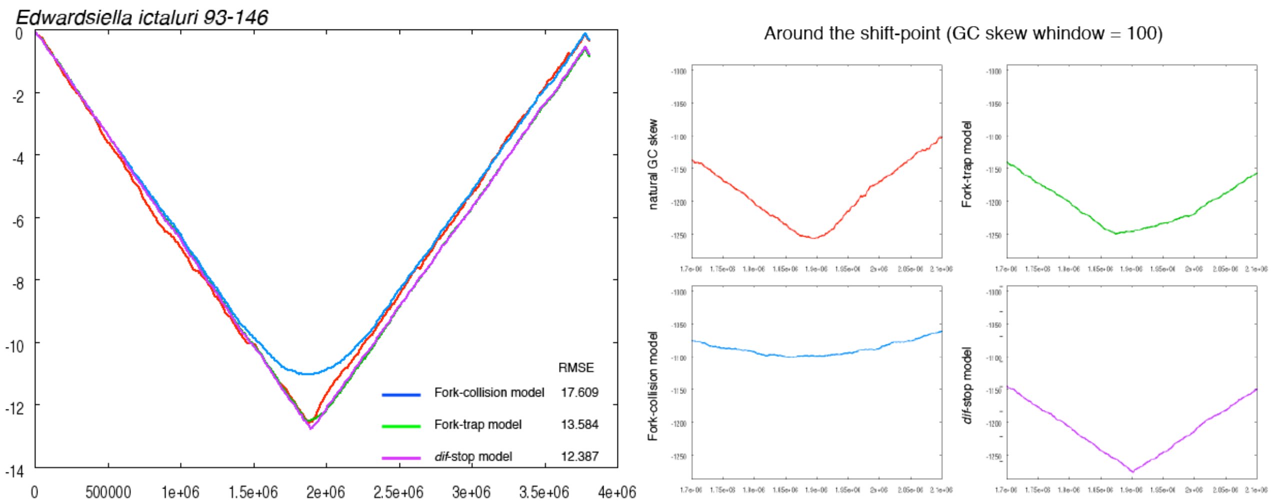
Edwardsiella ictaluri 93-146
Around the shift-point (GC skew whindow = 100)
RMSE
Fork-collision model 17.609
Fork-trap model 13.584
dif-stop model 12.387
natural GC skew
Fork-trap model
Fork-collision model
dif-stop model

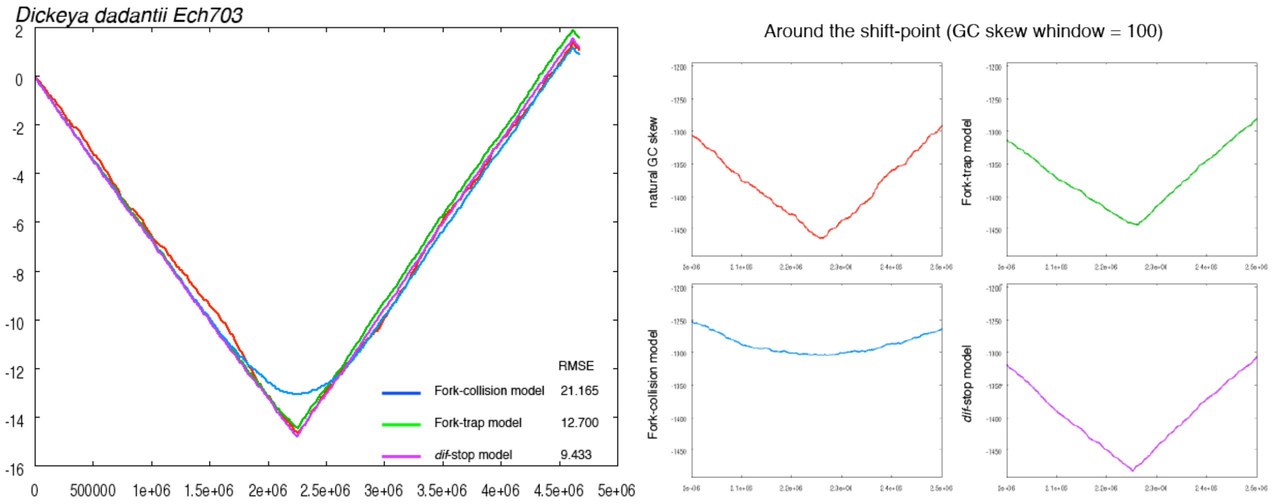
Dickeya dadantii Ech703
Around the shift-point (GC skew whindow = 100)
RMSE
Fork-collision model 21.165
Fork-trap model 12.700
dif-stop model 9.433
natural GC skew
Fork-trap model
Fork-collision model
dif-stop model

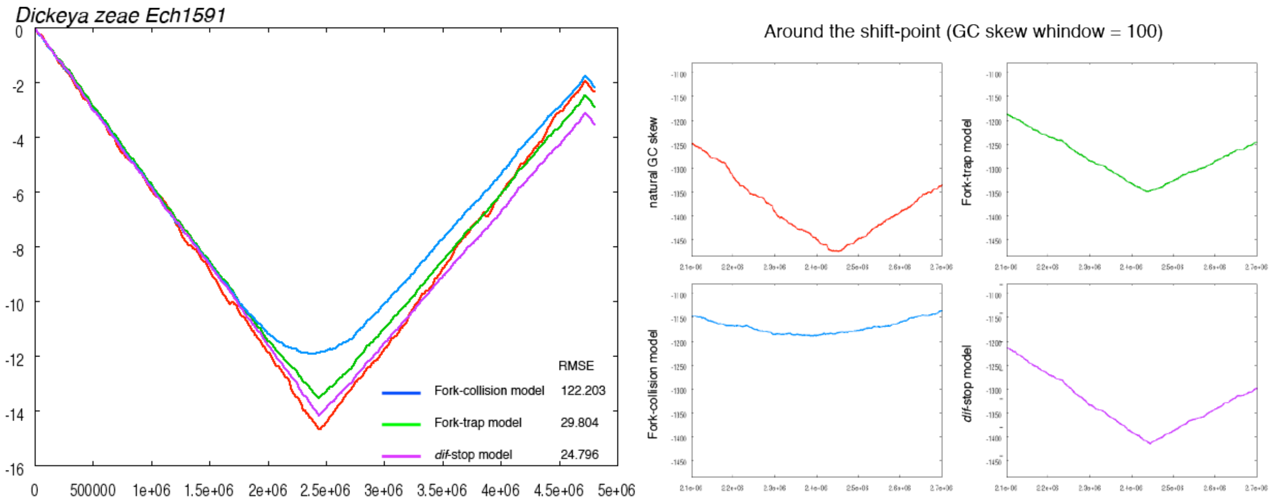
Dickeya zeae Ech1591
Around the shift-point (GC skew whindow = 100)
RMSE
Fork-collision model 122.203
Fork-trap model 29.804
dif-stop model 24.796
natural GC skew
Fork-trap model
Fork-collision model
dif-stop model

Escherichia coli B str. REL606

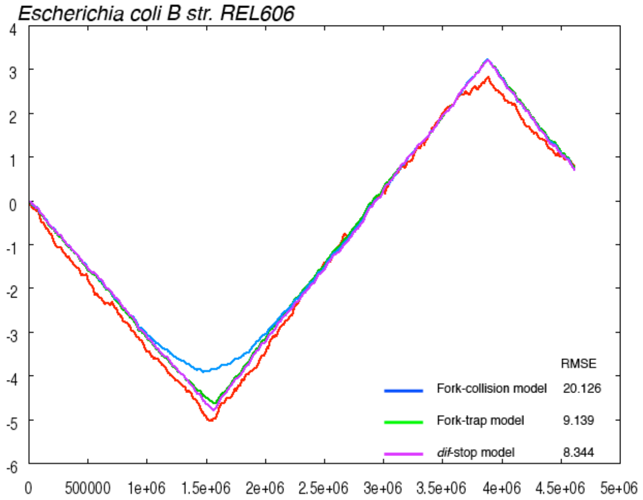

Around the shift-point (GC skew whindow = 100)

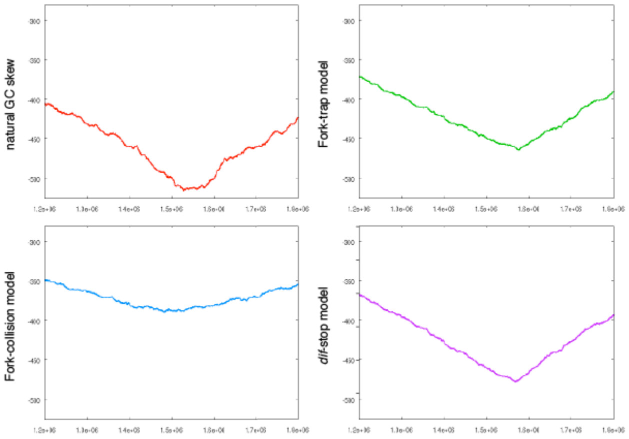

Escherichia coli O157:H7 str. TW14359

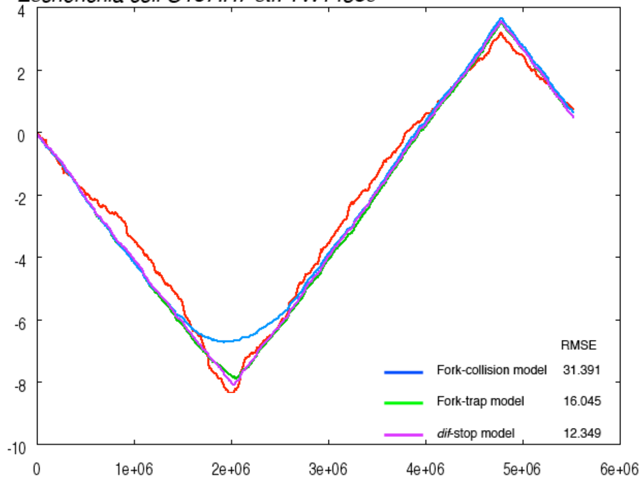

Around the shift-point (GC skew whindow = 100)

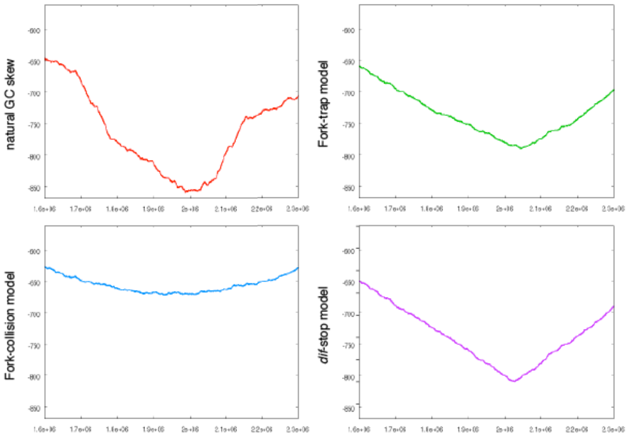

Supplement: Figure S3 — Simulation results in all target organisms. The GC skew simulation results of the overall shapes and close-up around the shift-points in all target organisms are shown. The left figures show the overall view of GC skew, and the regions surrounded by dashed lines around the shift-point are extended as the right figures. In the right figures, red, green, blue and purple lines represent the natural GC skew, fork-trap model, fork- collision model and dif-stop model, respectively. (PDF) [file pone.0034526.s003.pdf]
