# Supplementary material for: Validation of Bacterial Replication Termination Models Using Simulation of Genomic Mutations
Source: PLoS One. 2012 Apr 3;7(4):e34526. doi: 10.1371/journal.pone.0034526 (PMC3317982; doi:10.1371/journal.pone.0034526)
Supplement: Figure S4 — Simulation results with three termination models and combination models in all target organisms. Simulated GC skew graphs are shown, for the bacterial natural GC skew (red), fork-collision model (blue), fork-trap model (green), dif-stop model (purple), and probabilistic combination (light blue). (PDF) [file pone.0034526.s004.pdf]

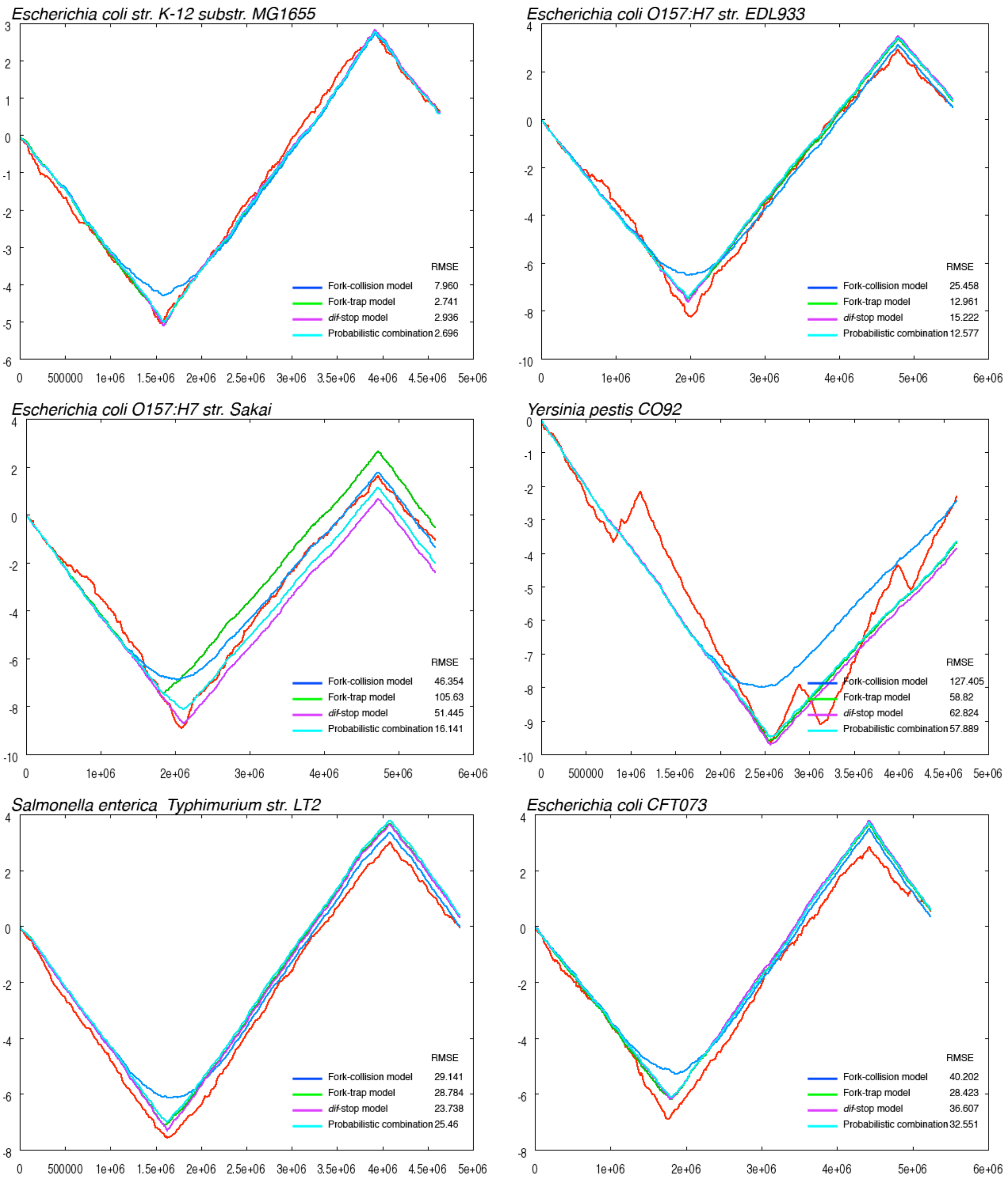
Escherichia coli str. K-12 substr. MG1655
RMSE
Fork-collision model 7.960
Fork-trap model 2.741
dif-stop model 2.936
Probabilistic combination 2.696
Escherichia coli O157:H7 str. EDL933
RMSE
Fork-collision model 25.458
Fork-trap model 12.961
dif-stop model 15.222
Probabilistic combination 12.577
Escherichia coli O157:H7 str. Sakai
RMSE
Fork-collision model 46.354
Fork-trap model 105.63
dif-stop model 51.445
Probabilistic combination 16.141
Yersinia pestis CO92
RMSE
Fork-collision model 127.405
Fork-trap model 58.82
dif-stop model 62.824
Probabilistic combination 57.889
Salmonella enterica Typhimurium str. LT2
RMSE
Fork-collision model 29.141
Fork-trap model 28.784
dif-stop model 23.738
Probabilistic combination 25.46
Escherichia coli CFT073
RMSE
Fork-collision model 40.202
Fork-trap model 28.423
dif-stop model 36.607
Probabilistic combination 32.551

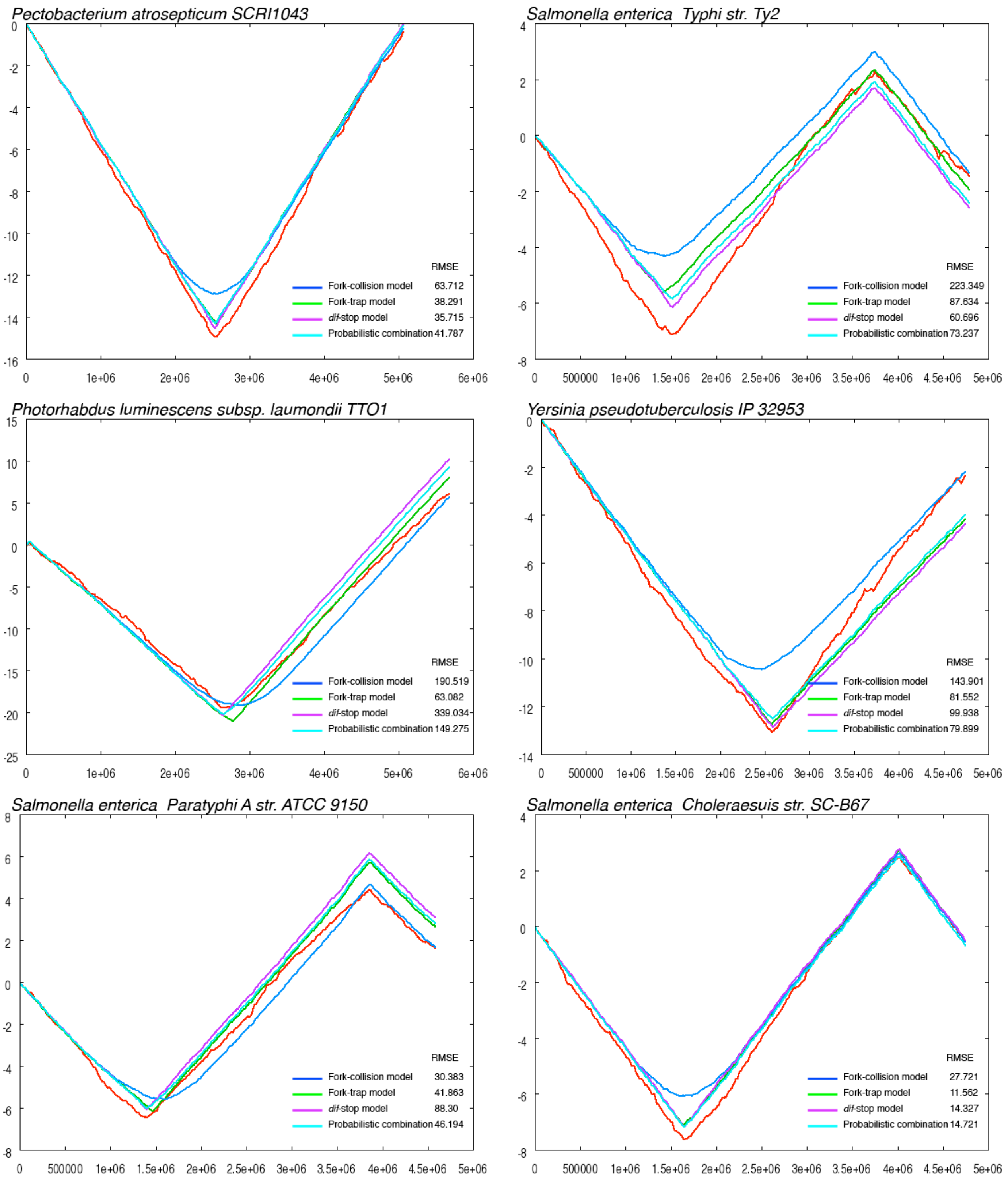

Pectobacterium atrosepticum SCRI1043
RMSE
Fork-collision model 63.712
Fork-trap model 38.291
dif-stop model 35.715
Probabilistic combination 41.787
Salmonella enterica Typhi str. Ty2
RMSE
Fork-collision model 223.349
Fork-trap model 87.634
dif-stop model 60.696
Probabilistic combination 73.237
Photorhabdus luminescens subsp. laumondii TTO1
RMSE
Fork-collision model 190.519
Fork-trap model 63.082
dif-stop model 339.034
Probabilistic combination 149.275
Yersinia pseudotuberculosis IP 32953
RMSE
Fork-collision model 143.901
Fork-trap model 81.552
dif-stop model 99.938
Probabilistic combination 79.899
Salmonella enterica Paratyphi A str. ATCC 9150
RMSE
Fork-collision model 30.383
Fork-trap model 41.863
dif-stop model 88.30
Probabilistic combination 46.194
Salmonella enterica Choleraesuis str. SC-B67
RMSE
Fork-collision model 27.721
Fork-trap model 11.562
dif-stop model 14.327
Probabilistic combination 14.721

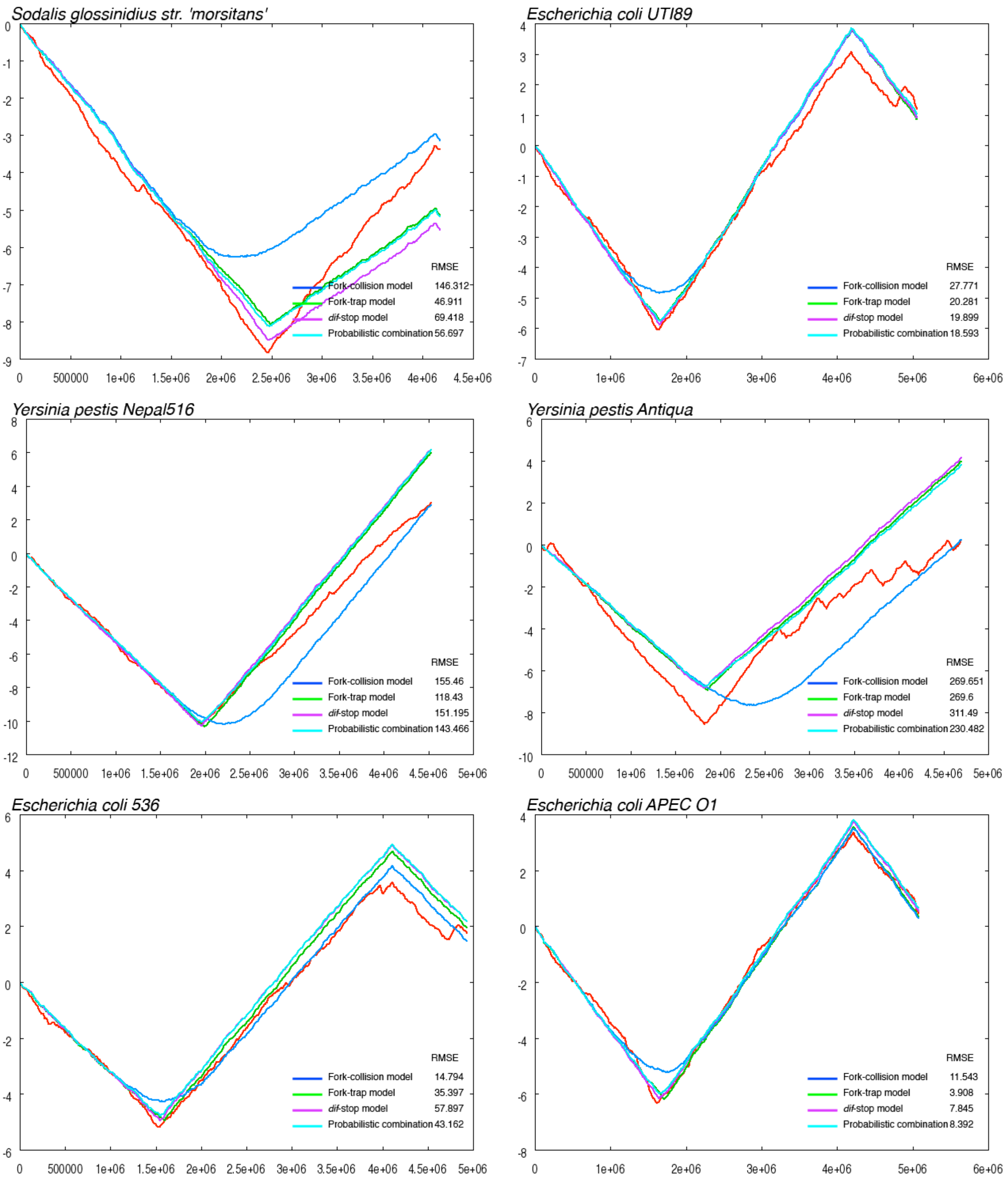

Sodalis glossinidius str. 'morsitans'
RMSE
Fork-collision model 146.312
Fork-trap model 46.911
dif-stop model 69.418
Probabilistic combination 56.697
Escherichia coli UTI89
RMSE
Fork-collision model 27.771
Fork-trap model 20.281
dif-stop model 19.899
Probabilistic combination 18.593
Yersinia pestis Nepal516
RMSE
Fork-collision model 155.46
Fork-trap model 118.43
dif-stop model 151.195
Probabilistic combination 143.466
Yersinia pestis Antiqua
RMSE
Fork-collision model 269.651
Fork-trap model 269.6
dif-stop model 311.49
Probabilistic combination 230.482
Escherichia coli 536
RMSE
Fork-collision model 14.794
Fork-trap model 35.397
dif-stop model 57.897
Probabilistic combination 43.162
Escherichia coli APEC O1
RMSE
Fork-collision model 11.543
Fork-trap model 3.908
dif-stop model 7.845
Probabilistic combination 8.392

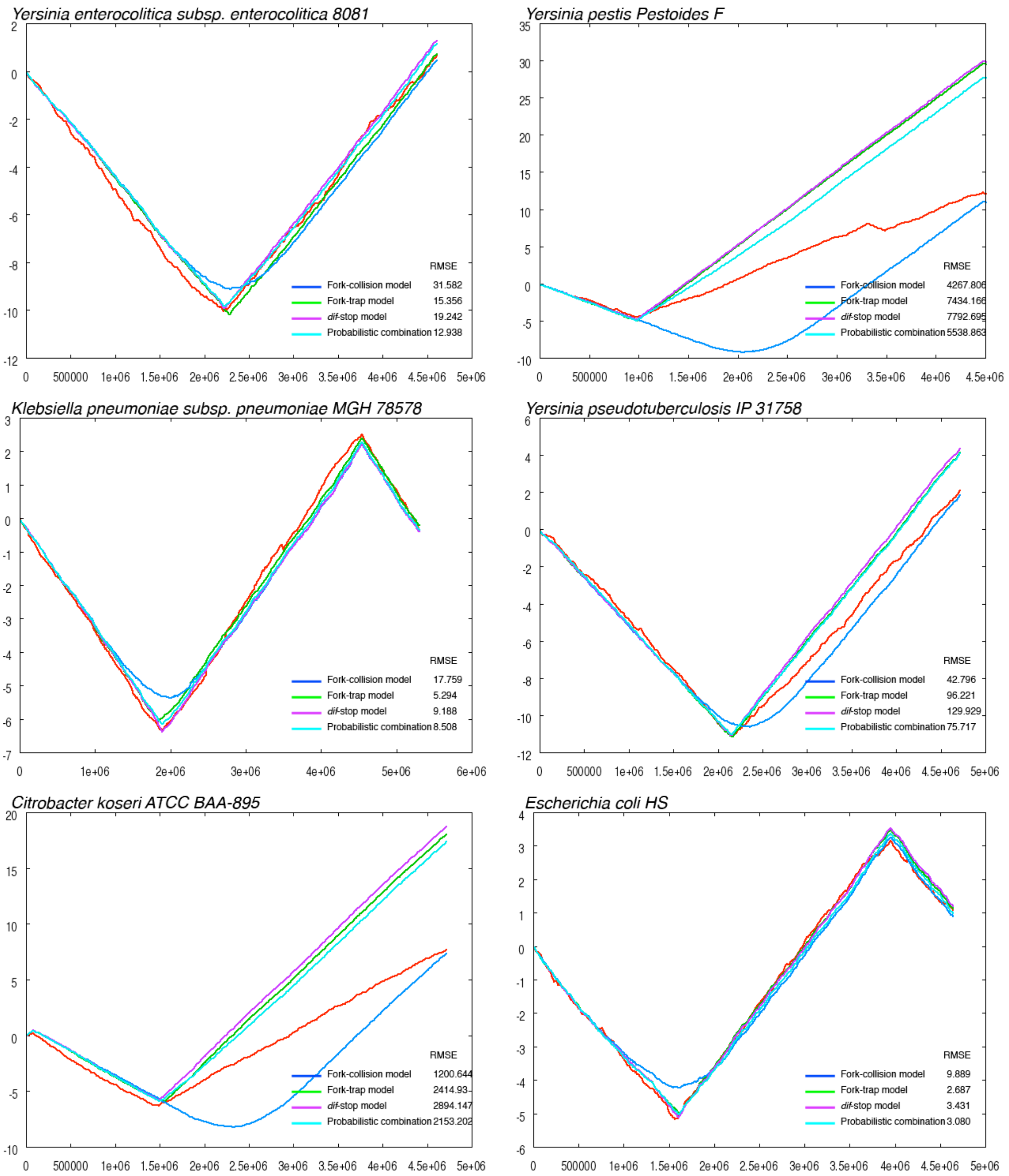
Yersinia enterocolitica subsp. enterocolitica 8081
RMSE
Fork-collision model 31.582
Fork-trap model 15.356
dif-stop model 19.242
Probabilistic combination 12.938
Yersinia pestis Pestoides F
RMSE
Fork-collision model 4267.806
Fork-trap model 7434.166
dif-stop model 7792.695
Probabilistic combination 5538.863
Klebsiella pneumoniae subsp. pneumoniae MGH 78578
RMSE
Fork-collision model 17.759
Fork-trap model 5.294
dif-stop model 9.188
Probabilistic combination 8.508
Yersinia pseudotuberculosis IP 31758
RMSE
Fork-collision model 42.796
Fork-trap model 96.221
dif-stop model 129.929
Probabilistic combination 75.717
Citrobacter koseri ATCC BAA-895
RMSE
Fork-collision model 1200.644
Fork-trap model 2414.93
dif-stop model 2894.147
Probabilistic combination 2153.202
Escherichia coli HS
RMSE
Fork-collision model 9.889
Fork-trap model 2.687
dif-stop model 3.431
Probabilistic combination 3.080

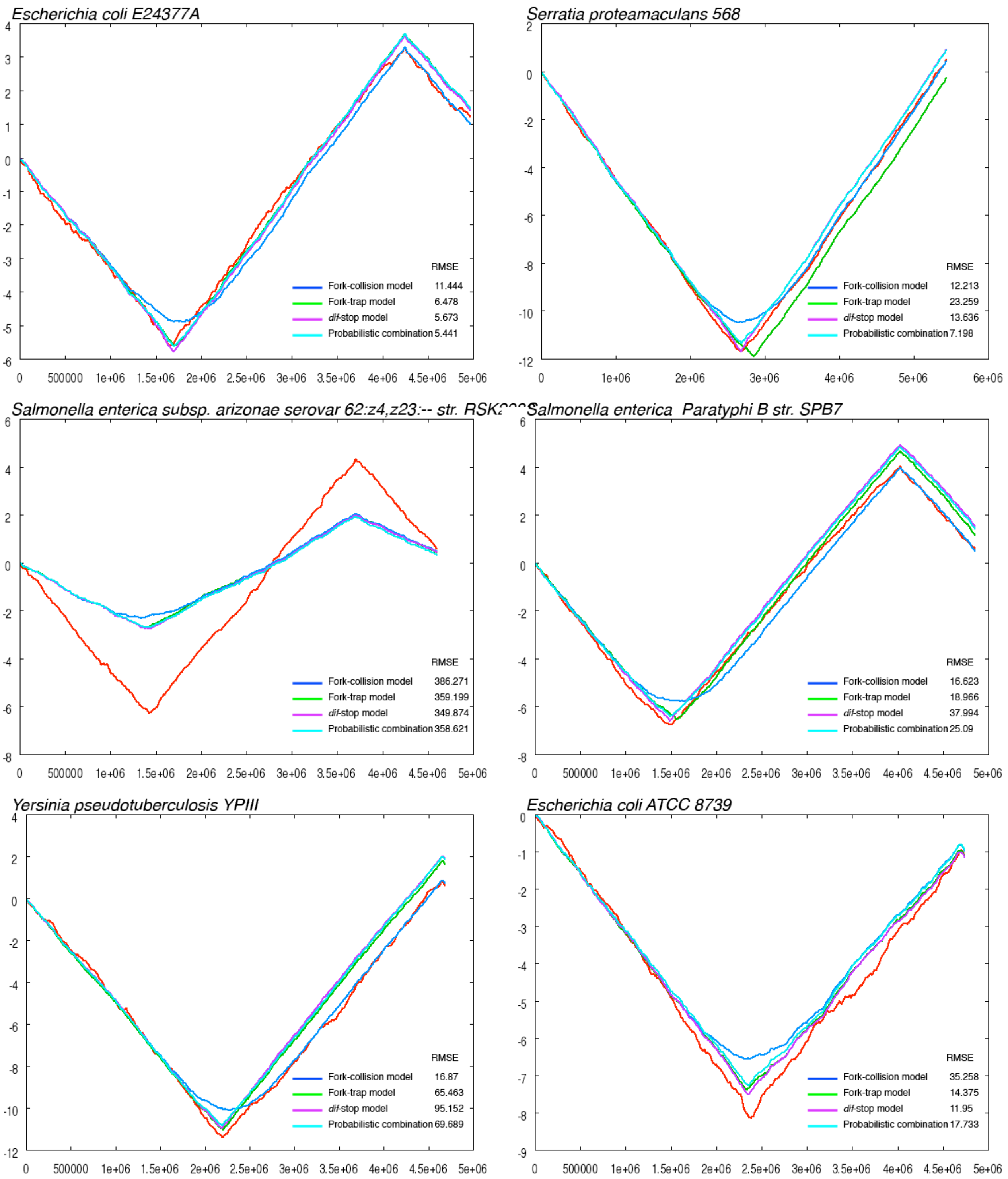
Escherichia coli E24377A
RMSE
Fork-collision model 11.444
Fork-trap model 6.478
dif-stop model 5.673
Probabilistic combination 5.441
Serratia proteamaculans 568
RMSE
Fork-collision model 12.213
Fork-trap model 23.259
dif-stop model 13.636
Probabilistic combination 7.198
Salmonella enterica subsp. arizonae serovar 62:z4,z23:-- str. RSK2
RMSE
Fork-collision model 386.271
Fork-trap model 359.199
dif-stop model 349.874
Probabilistic combination 358.621
Salmonella enterica Paratyphi B str. SPB7
RMSE
Fork-collision model 16.623
Fork-trap model 18.966
dif-stop model 37.994
Probabilistic combination 25.09
Yersinia pseudotuberculosis YPIII
RMSE
Fork-collision model 16.87
Fork-trap model 65.463
dif-stop model 95.152
Probabilistic combination 69.689
Escherichia coli ATCC 8739
RMSE
Fork-collision model 35.258
Fork-trap model 14.375
dif-stop model 11.95
Probabilistic combination 17.733

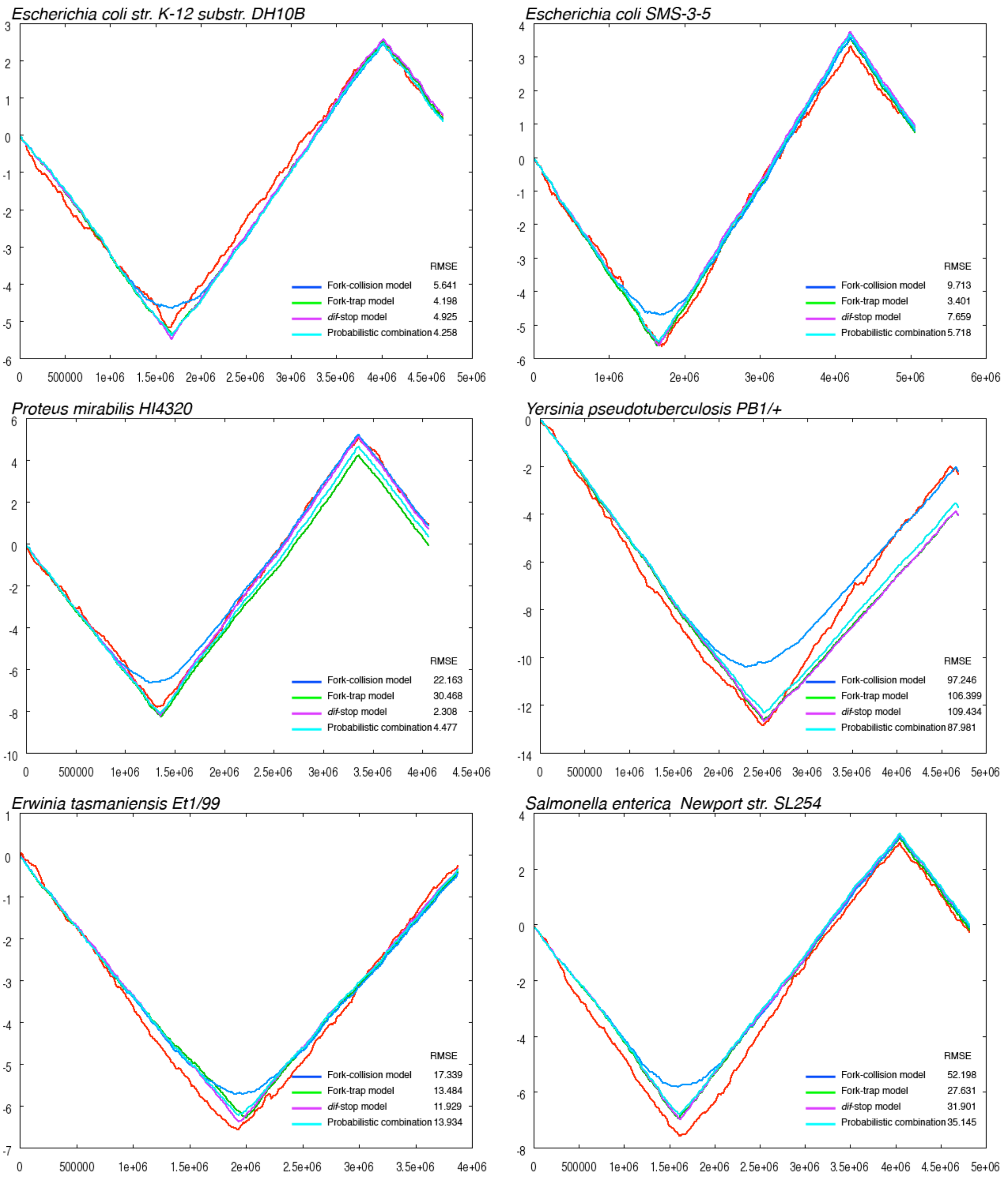
Escherichia coli str. K-12 substr. DH10B
RMSE
Fork-collision model 5.641
Fork-trap model 4.198
dif-stop model 4.925
Probabilistic combination 4.258
Escherichia coli SMS-3-5
RMSE
Fork-collision model 9.713
Fork-trap model 3.401
dif-stop model 7.659
Probabilistic combination 5.718
Proteus mirabilis HI4320
RMSE
Fork-collision model 22.163
Fork-trap model 30.468
dif-stop model 2.308
Probabilistic combination 4.477
Yersinia pseudotuberculosis PB1/+
RMSE
Fork-collision model 97.246
Fork-trap model 106.399
dif-stop model 109.434
Probabilistic combination 87.981
Erwinia tasmaniensis Et1/99
RMSE
Fork-collision model 17.339
Fork-trap model 13.484
dif-stop model 11.929
Probabilistic combination 13.934
Salmonella enterica Newport str. SL254
RMSE
Fork-collision model 52.198
Fork-trap model 27.631
dif-stop model 31.901
Probabilistic combination 35.145

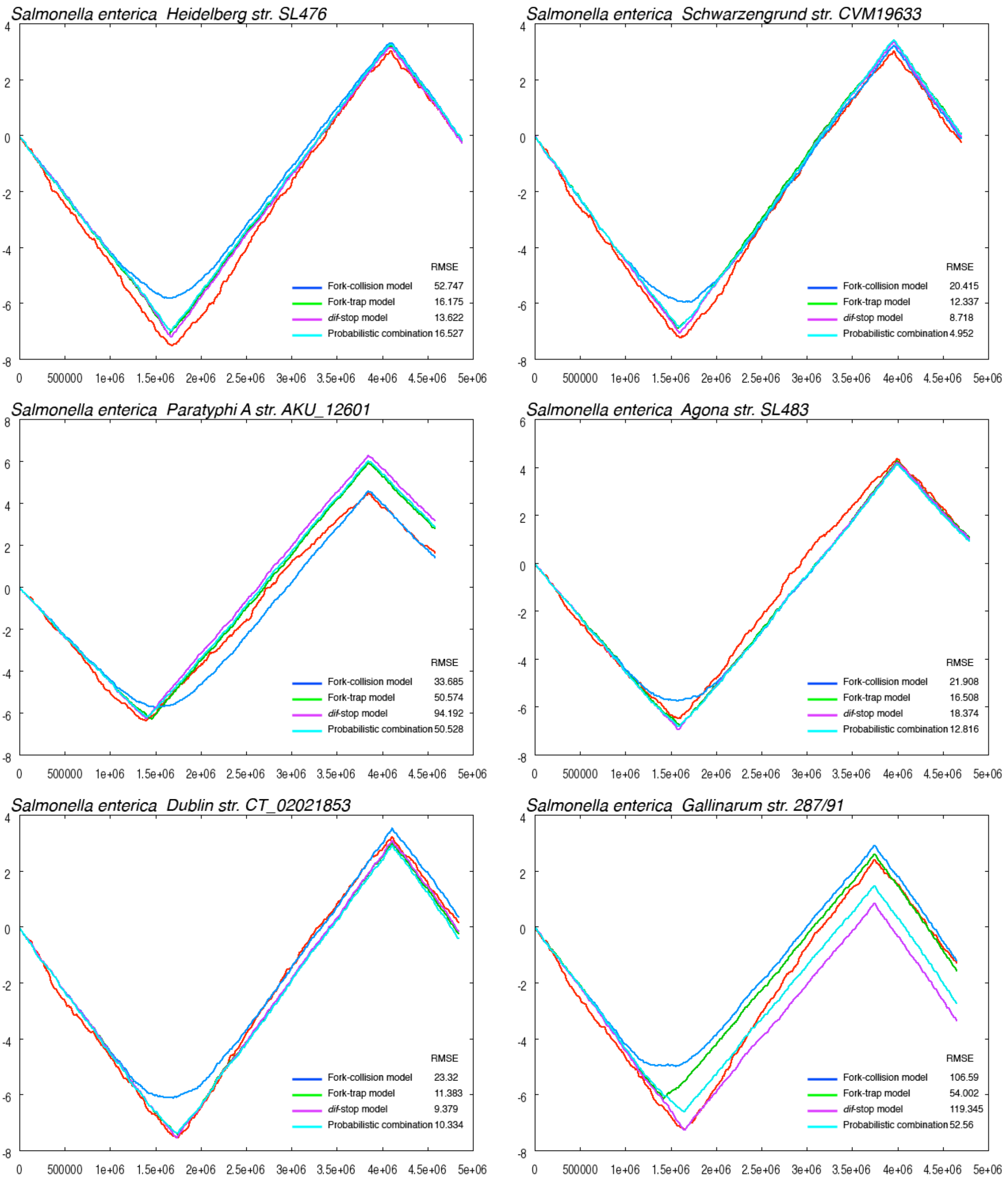

Salmonella enterica Heidelberg str. SL476
RMSE
Fork-collision model 52.747
Fork-trap model 16.175
dif-stop model 13.622
Probabilistic combination 16.527
Salmonella enterica Schwarzengrund str. CVM19633
RMSE
Fork-collision model 20.415
Fork-trap model 12.337
dif-stop model 8.718
Probabilistic combination 4.952
Salmonella enterica Paratyphi A str. AKU_12601
RMSE
Fork-collision model 33.685
Fork-trap model 50.574
dif-stop model 94.192
Probabilistic combination 50.528
Salmonella enterica Agona str. SL483
RMSE
Fork-collision model 21.908
Fork-trap model 16.508
dif-stop model 18.374
Probabilistic combination 12.816
Salmonella enterica Dublin str. CT_02021853
RMSE
Fork-collision model 23.32
Fork-trap model 11.383
dif-stop model 9.379
Probabilistic combination 10.334
Salmonella enterica Gallinarum str. 287/91
RMSE
Fork-collision model 106.59
Fork-trap model 54.002
dif-stop model 119.345
Probabilistic combination 52.56

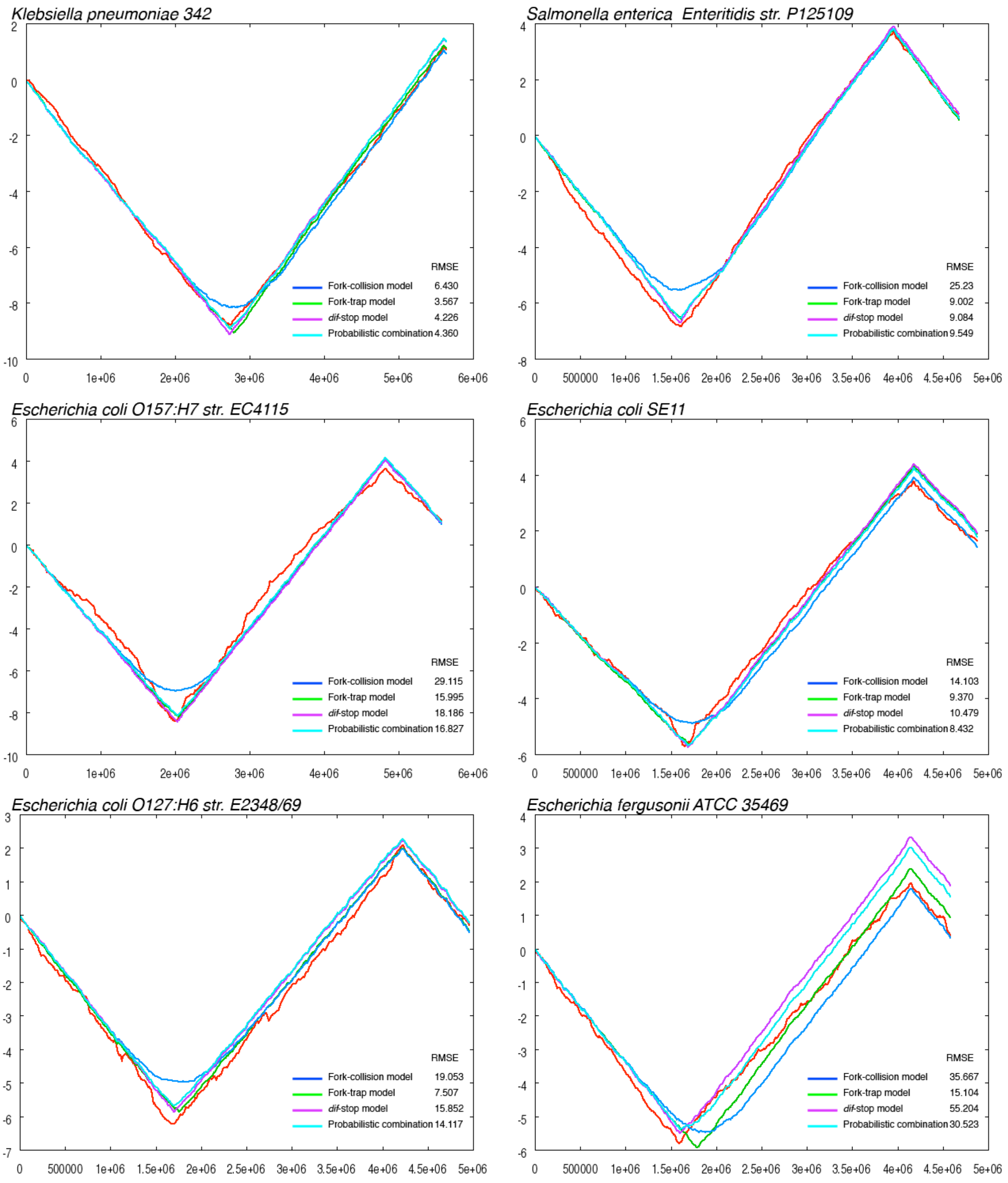
Klebsiella pneumoniae 342
RMSE
Fork-collision model 6.430
Fork-trap model 3.567
dif-stop model 4.226
Probabilistic combination 4.360
Salmonella enterica Enteritidis str. P125109
RMSE
Fork-collision model 25.23
Fork-trap model 9.002
dif-stop model 9.084
Probabilistic combination 9.549
Escherichia coli O157:H7 str. EC4115
RMSE
Fork-collision model 29.115
Fork-trap model 15.995
dif-stop model 18.186
Probabilistic combination 16.827
Escherichia coli SE11
RMSE
Fork-collision model 14.103
Fork-trap model 9.370
dif-stop model 10.479
Probabilistic combination 8.432
Escherichia coli O127:H6 str. E2348/69
RMSE
Fork-collision model 19.053
Fork-trap model 7.507
dif-stop model 15.852
Probabilistic combination 14.117
Escherichia fergusonii ATCC 35469
RMSE
Fork-collision model 35.667
Fork-trap model 15.104
dif-stop model 55.204
Probabilistic combination 30.523

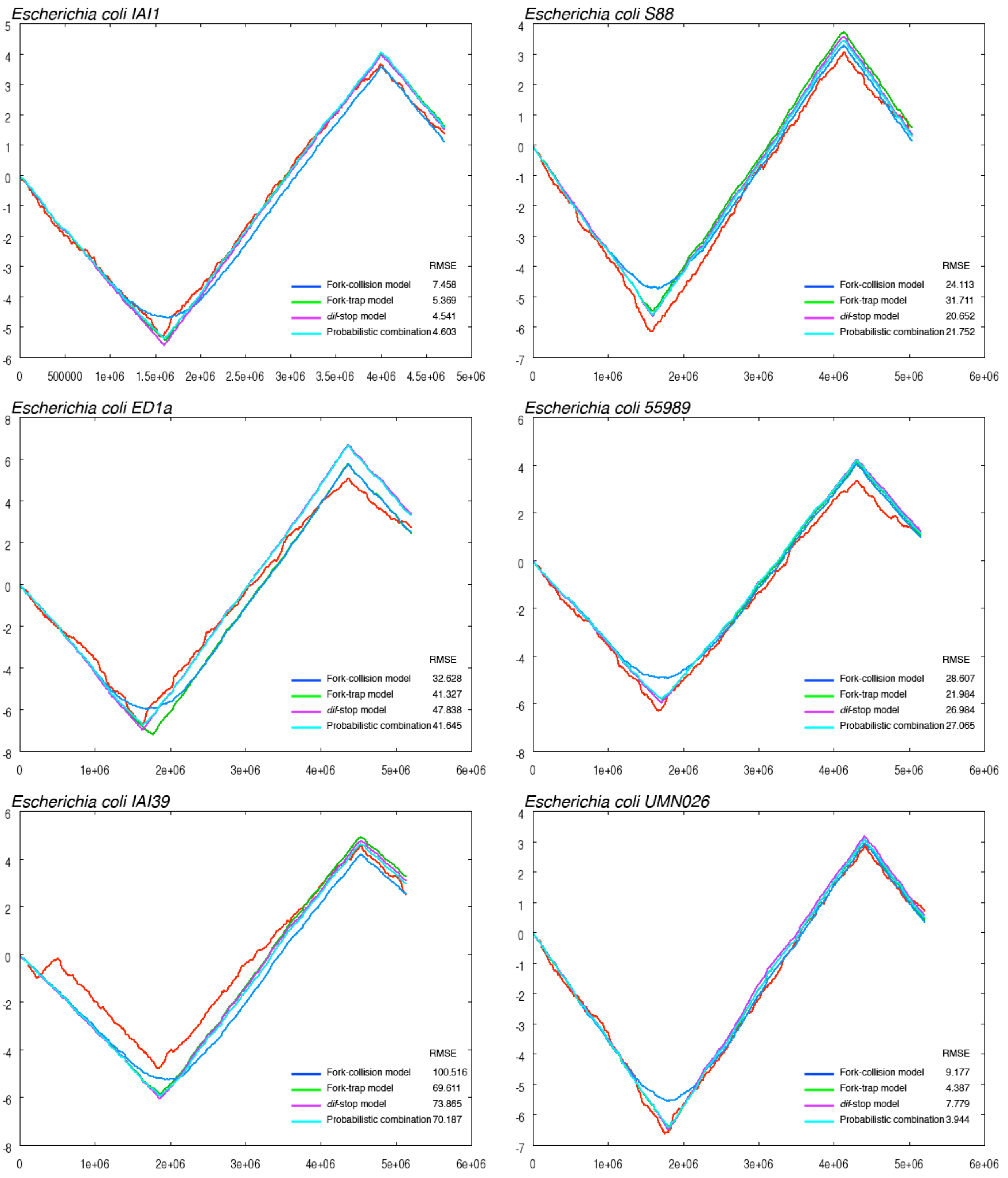

Escherichia coli IAI1
RMSE
Fork-collision model 7.458
Fork-trap model 5.369
dif-stop model 4.541
Probabilistic combination 4.603
0 500000 1e+06 1.5e+06 2e+06 2.5e+06 3e+06 3.5e+06 4e+06 4.5e+06 5e+06
Escherichia coli S88
RMSE
Fork-collision model 24.113
Fork-trap model 31.711
dif-stop model 20.652
Probabilistic combination 21.752
0 1e+06 2e+06 3e+06 4e+06 5e+06 6e+06
Escherichia coli ED1a
RMSE
Fork-collision model 32.628
Fork-trap model 41.327
dif-stop model 47.838
Probabilistic combination 41.645
0 1e+06 2e+06 3e+06 4e+06 5e+06 6e+06
Escherichia coli 55989
RMSE
Fork-collision model 28.607
Fork-trap model 21.984
dif-stop model 26.984
Probabilistic combination 27.065
0 1e+06 2e+06 3e+06 4e+06 5e+06 6e+06
Escherichia coli IAI39
RMSE
Fork-collision model 100.516
Fork-trap model 69.611
dif-stop model 73.865
Probabilistic combination 70.187
0 1e+06 2e+06 3e+06 4e+06 5e+06 6e+06
Escherichia coli UMN026
RMSE
Fork-collision model 9.177
Fork-trap model 4.387
dif-stop model 7.779
Probabilistic combination 3.944
0 1e+06 2e+06 3e+06 4e+06 5e+06 6e+06

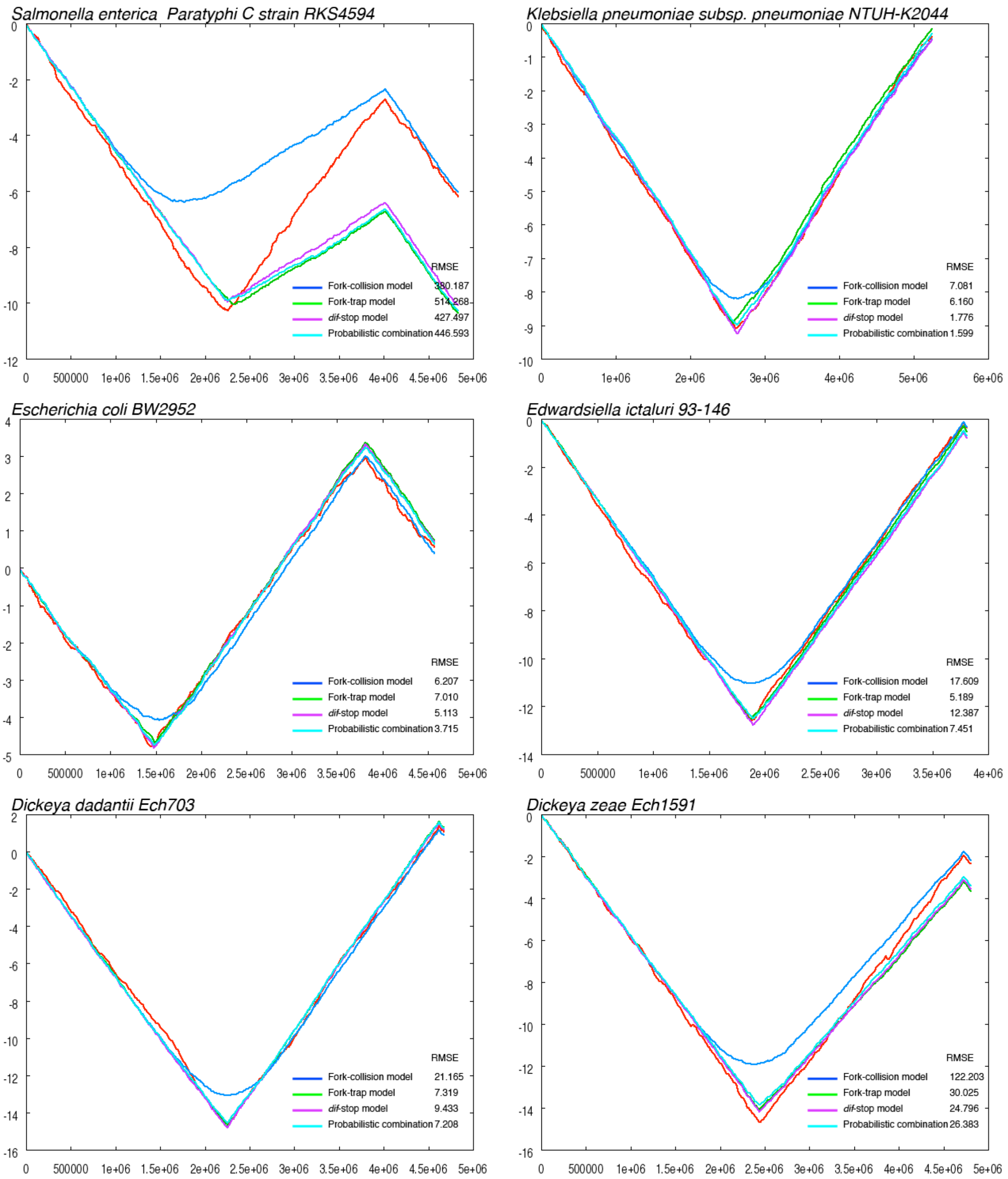
Salmonella enterica Paratyphi C strain RKS4594
RMSE
Fork-collision model 380.187
Fork-trap model 514.268
dif-stop model 427.497
Probabilistic combination 446.593
Klebsiella pneumoniae subsp. pneumoniae NTUH-K2044
RMSE
Fork-collision model 7.081
Fork-trap model 6.160
dif-stop model 1.776
Probabilistic combination 1.599
Escherichia coli BW2952
RMSE
Fork-collision model 6.207
Fork-trap model 7.010
dif-stop model 5.113
Probabilistic combination 3.715
Edwardsiella ictaluri 93-146
RMSE
Fork-collision model 17.609
Fork-trap model 5.189
dif-stop model 12.387
Probabilistic combination 7.451
Dickeya dadantii Ech703
RMSE
Fork-collision model 21.165
Fork-trap model 7.319
dif-stop model 9.433
Probabilistic combination 7.208
Dickeya zeae Ech1591
RMSE
Fork-collision model 122.203
Fork-trap model 30.025
dif-stop model 24.796
Probabilistic combination 26.383

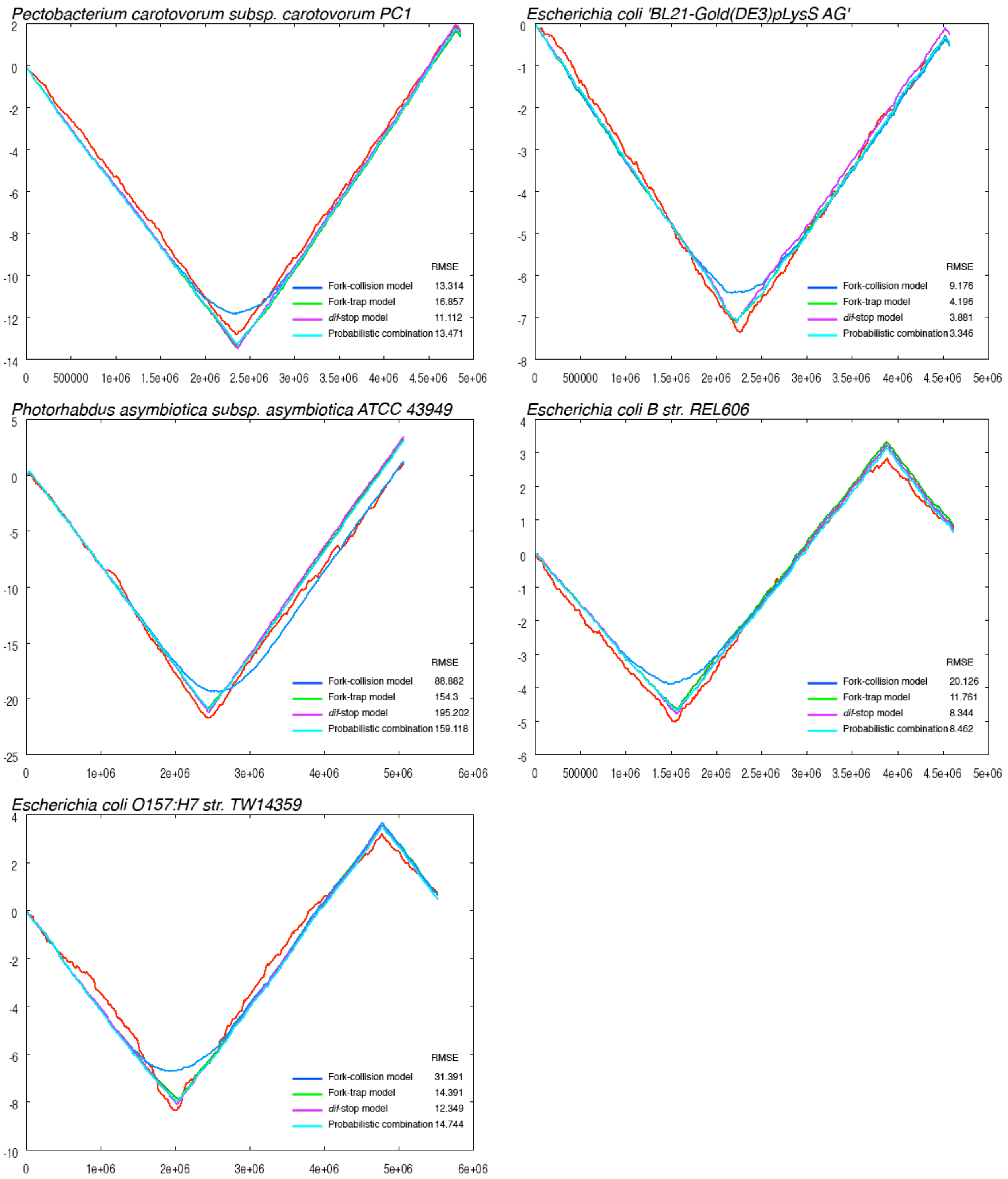
Pectobacterium carotovorum subsp. carotovorum PC1
RMSE
Fork-collision model 13.314
Fork-trap model 16.857
dif-stop model 11.112
Probabilistic combination 13.471
Escherichia coli 'BL21-Gold(DE3)pLysS AG'
RMSE
Fork-collision model 9.176
Fork-trap model 4.196
dif-stop model 3.881
Probabilistic combination 3.346
Photorhabdus asymbiotica subsp. asymbiotica ATCC 43949
RMSE
Fork-collision model 88.882
Fork-trap model 154.3
dif-stop model 195.202
Probabilistic combination 159.118
Escherichia coli B str. REL606
RMSE
Fork-collision model 20.126
Fork-trap model 11.761
dif-stop model 8.344
Probabilistic combination 8.462
Escherichia coli O157:H7 str. TW14359
RMSE
Fork-collision model 31.391
Fork-trap model 14.391
dif-stop model 12.349
Probabilistic combination 14.744
