# Supplementary material for: Validation of Bacterial Replication Termination Models Using Simulation of Genomic Mutations
Source: PLoS One. 2012 Apr 3;7(4):e34526. doi: 10.1371/journal.pone.0034526 (PMC3317982; doi:10.1371/journal.pone.0034526)
Supplement: Figure S8 — The replication fork pausing rates. The x-axes represent the genome positions and the y-axes represents the percentages of pausing rates. In each bacterium, these pausing rates, which were calculated based on the experimental evidences, are very similar with the optimized pausing rates (R = 0.725, Spearman rank-correlation coefficient). (PDF) [file pone.0034526.s008.pdf]

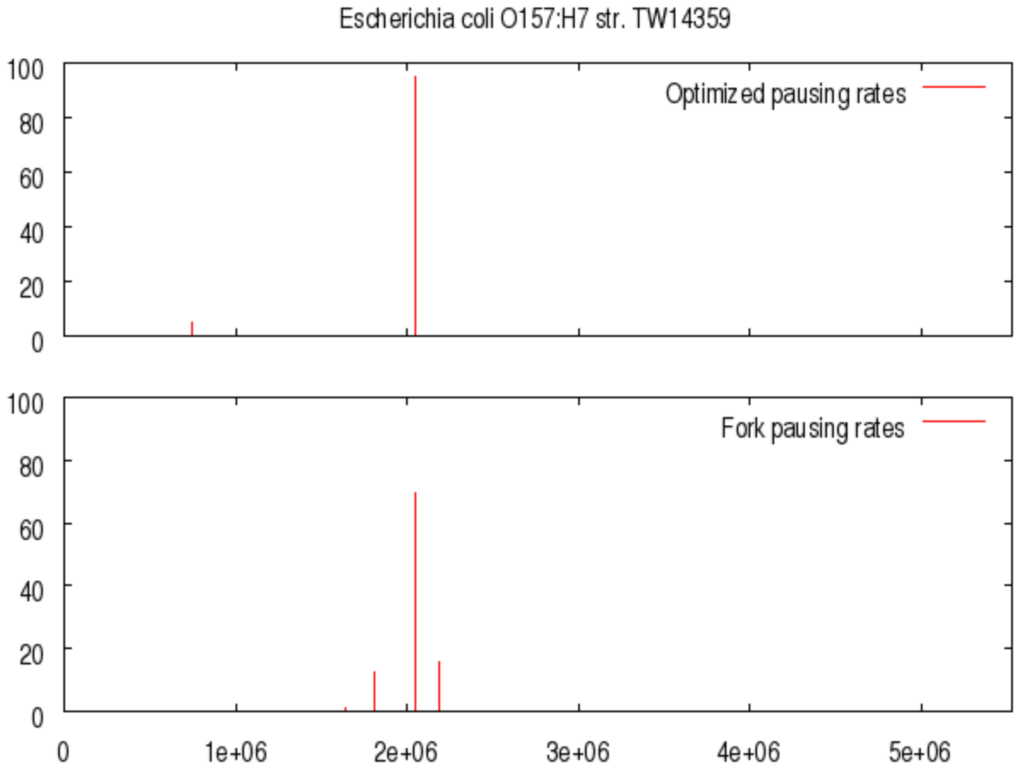
Escherichia coli O157:H7 str. TW14359
Optimized pausing rates
Fork pausing rates
0
20
40
60
80
100
0
1e+06
2e+06
3e+06
4e+06
5e+06

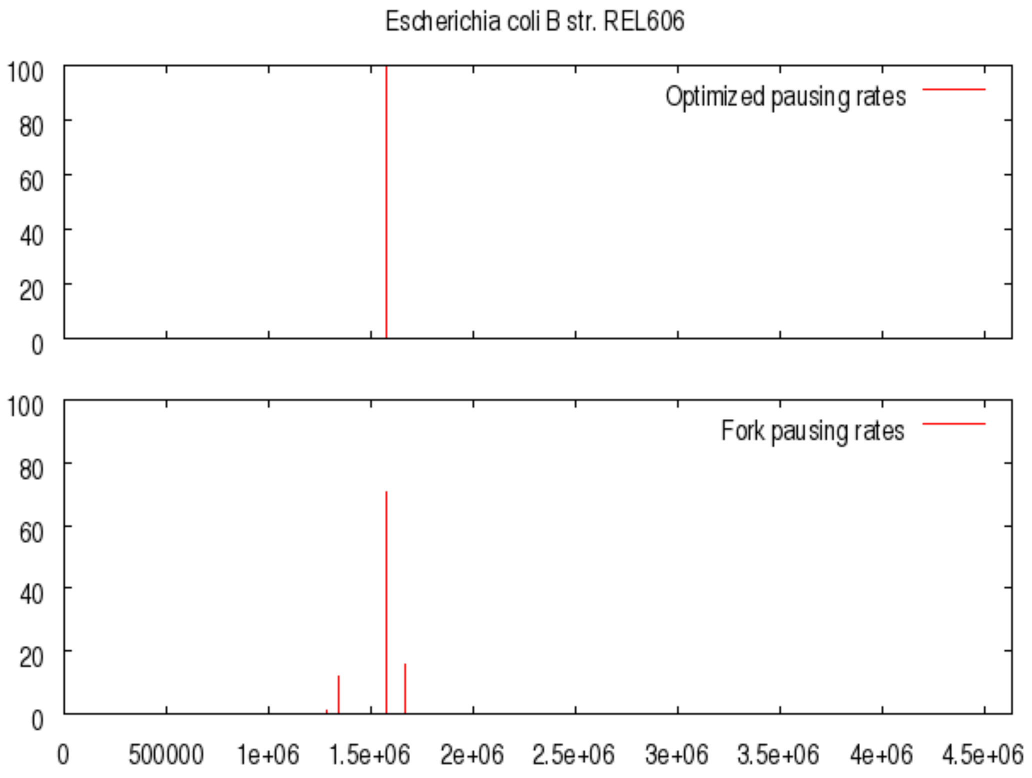
Escherichia coli B str. REL606
Optimized pausing rates
Fork pausing rates
0
20
40
60
80
100
0
500000
1e+06
1.5e+06
2e+06
2.5e+06
3e+06
3.5e+06
4e+06
4.5e+06

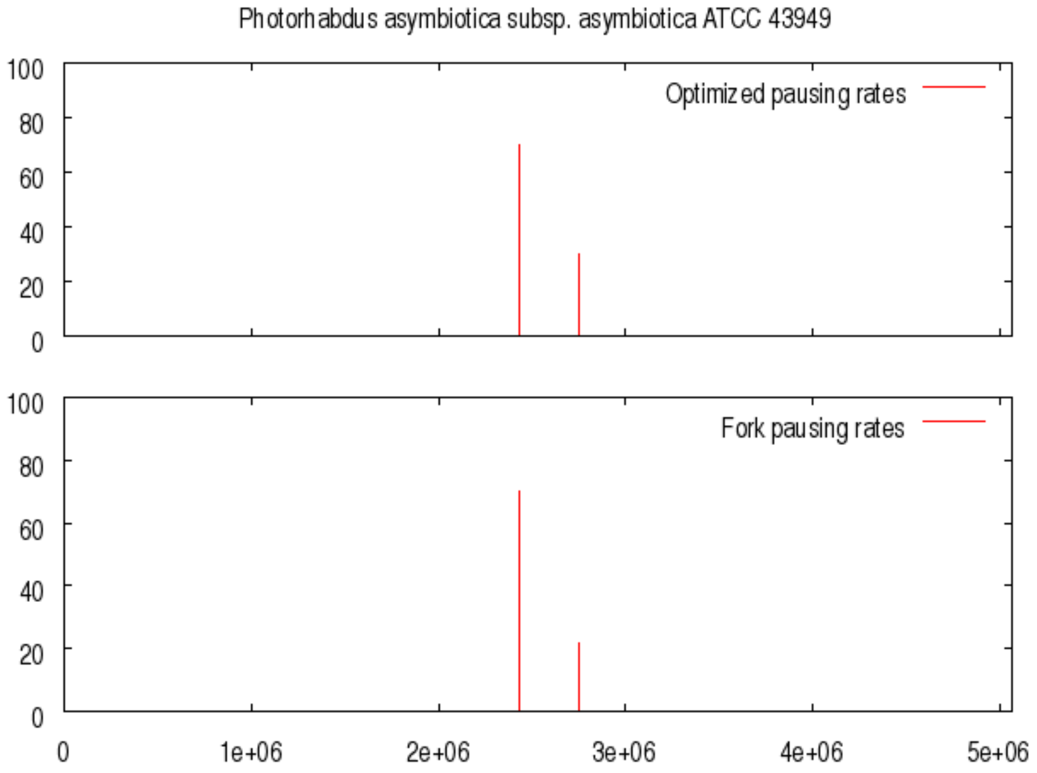
Photorhabdus asymbiotica subsp. asymbiotica ATCC 43949
Optimized pausing rates
Fork pausing rates
100
80
60
40
20
0
1e+06
2e+06
3e+06
4e+06
5e+06

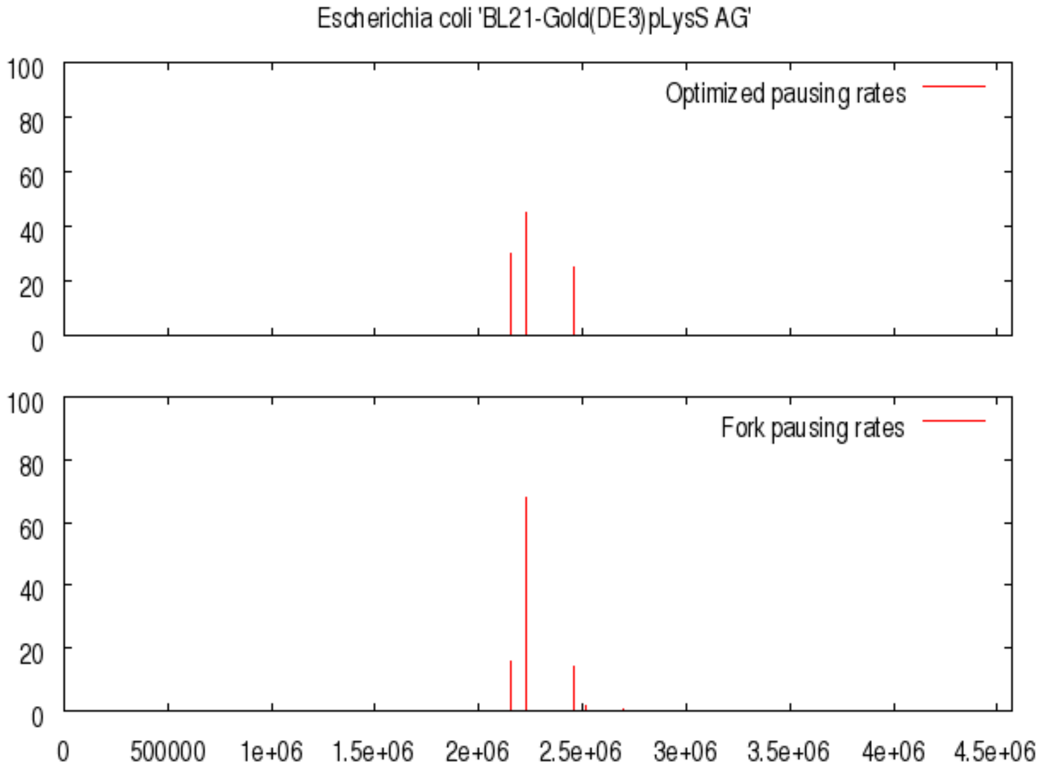
Escherichia coli 'BL21-Gold(DE3)pLysS AG'
Optimized pausing rates
Fork pausing rates
100
80
60
40
20
0
500000
1e+06
1.5e+06
2e+06
2.5e+06
3e+06
3.5e+06
4e+06
4.5e+06

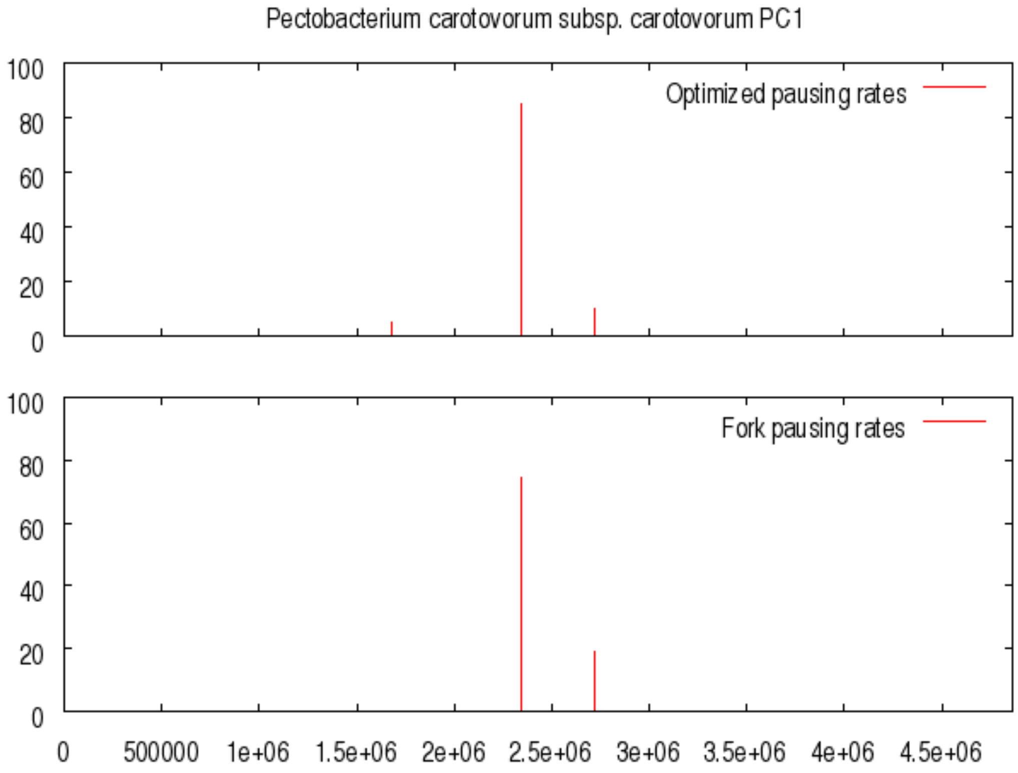
Pectobacterium carotovorum subsp. carotovorum PC1
Optimized pausing rates
Fork pausing rates
0
20
40
60
80
100
0
500000
1e+06
1.5e+06
2e+06
2.5e+06
3e+06
3.5e+06
4e+06
4.5e+06

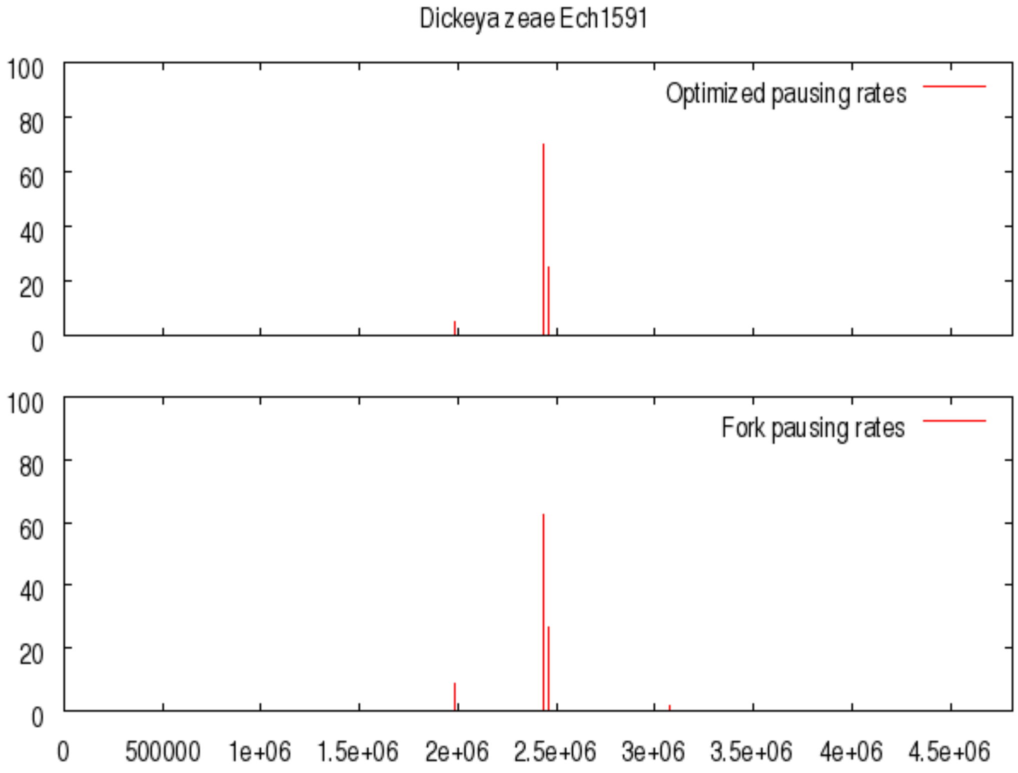
Dickeya zeae Ech1591
Optimized pausing rates
Fork pausing rates
0
20
40
60
80
100
0
500000
1e+06
1.5e+06
2e+06
2.5e+06
3e+06
3.5e+06
4e+06
4.5e+06

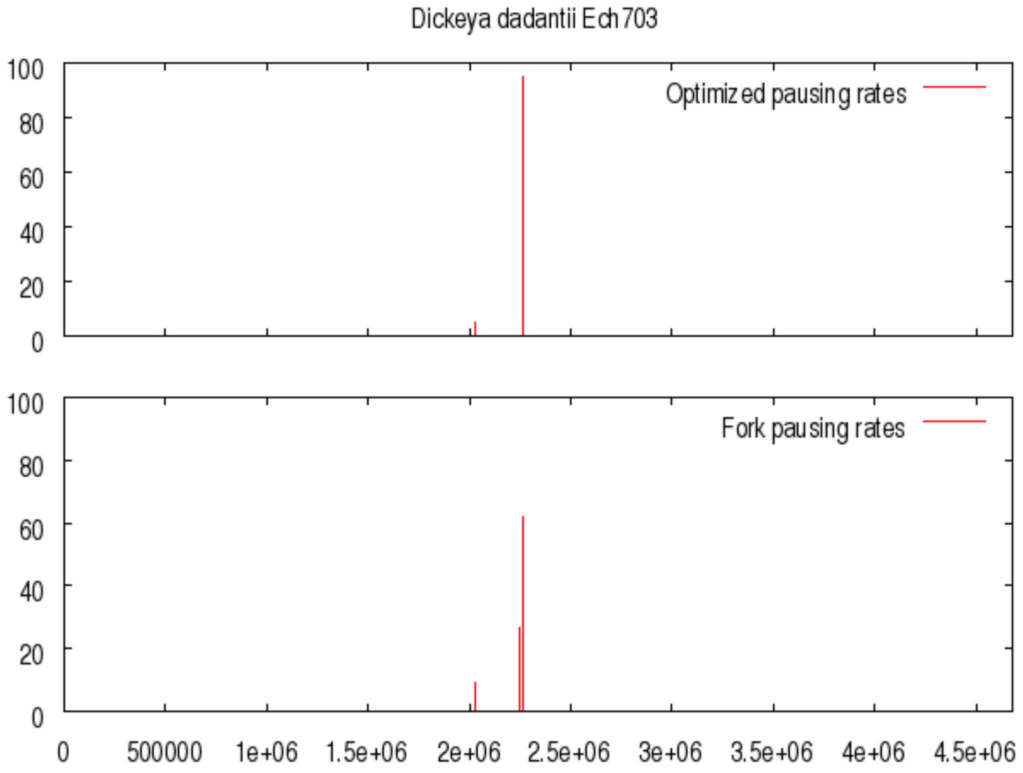
Dickeya dadantii Ech703
Optimized pausing rates
Fork pausing rates
100
80
60
40
20
0
0
500000
1e+06
1.5e+06
2e+06
2.5e+06
3e+06
3.5e+06
4e+06
4.5e+06

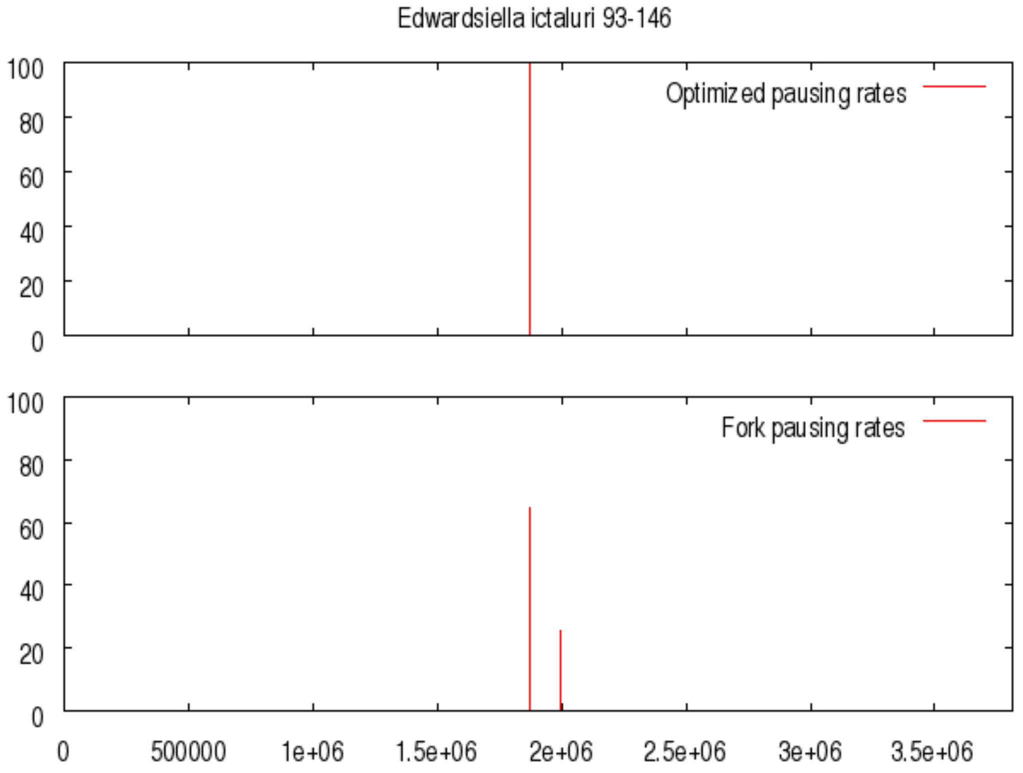
Edwardsiella ictaluri 93-146
Optimized pausing rates
Fork pausing rates
100
80
60
40
20
0
0
500000
1e+06
1.5e+06
2e+06
2.5e+06
3e+06
3.5e+06

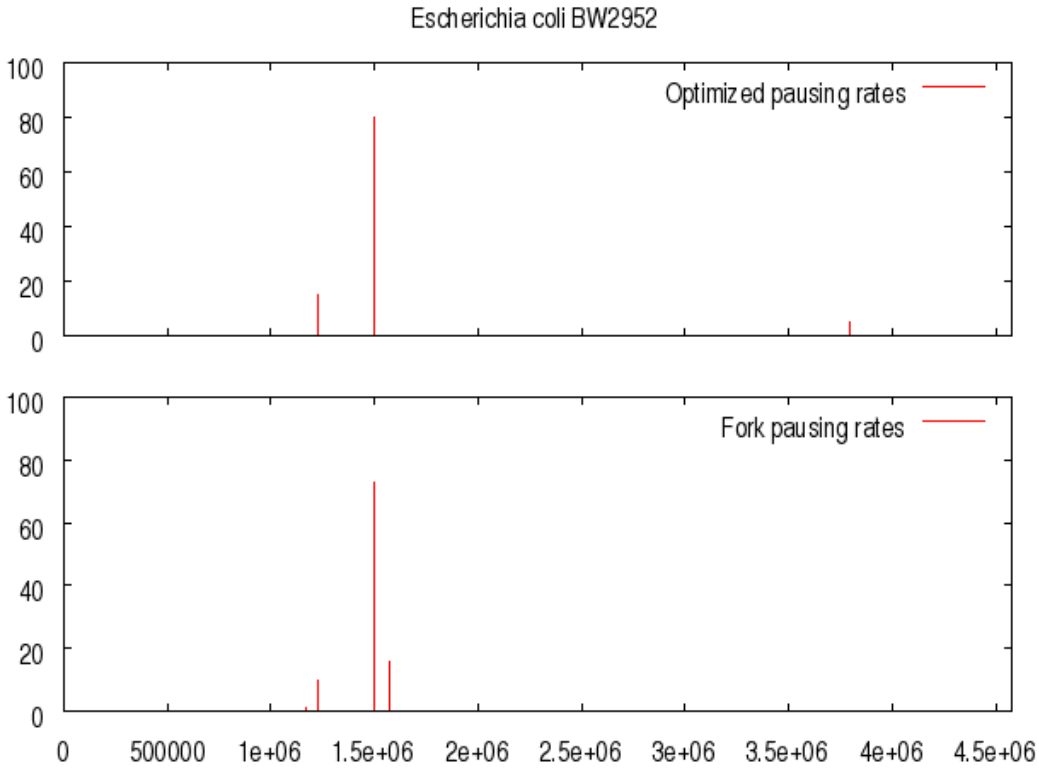
Escherichia coli BW2952
Optimized pausing rates
Fork pausing rates
100
80
60
40
20
0
500000
1e+06
1.5e+06
2e+06
2.5e+06
3e+06
3.5e+06
4e+06
4.5e+06

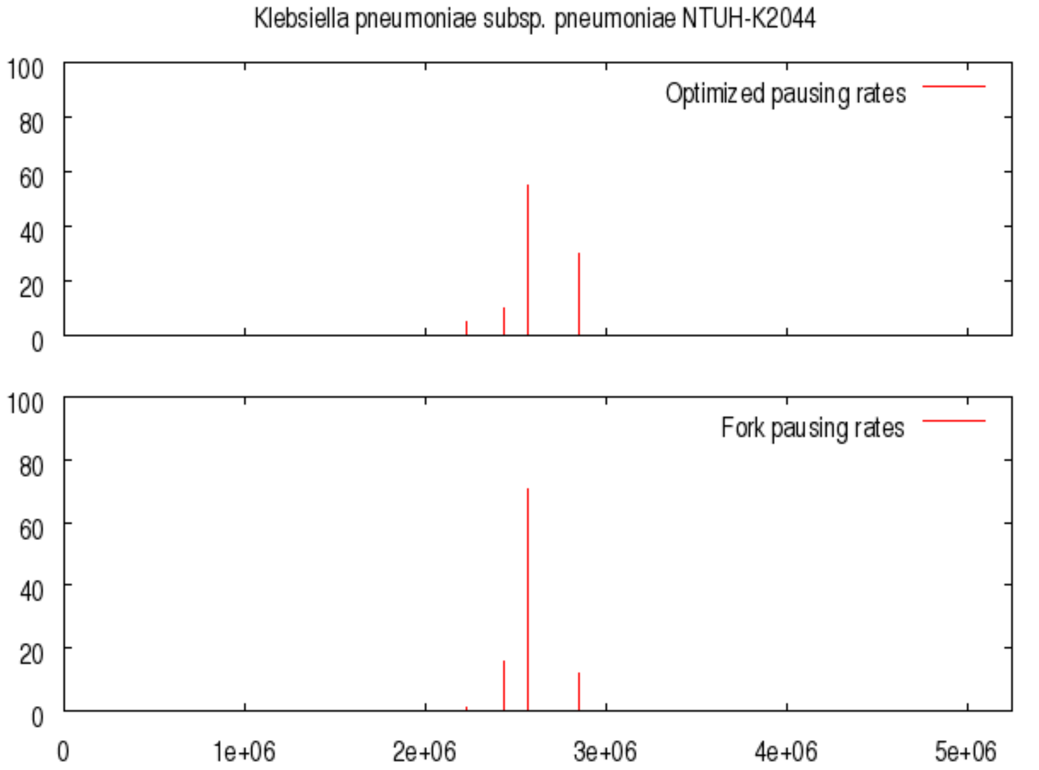
Klebsiella pneumoniae subsp. pneumoniae NTUH-K2044
Optimized pausing rates
Fork pausing rates
100
80
60
40
20
0
1e+06
2e+06
3e+06
4e+06
5e+06

Salmonella enterica subsp. enterica serovar Paratyphi C strain RKS4594

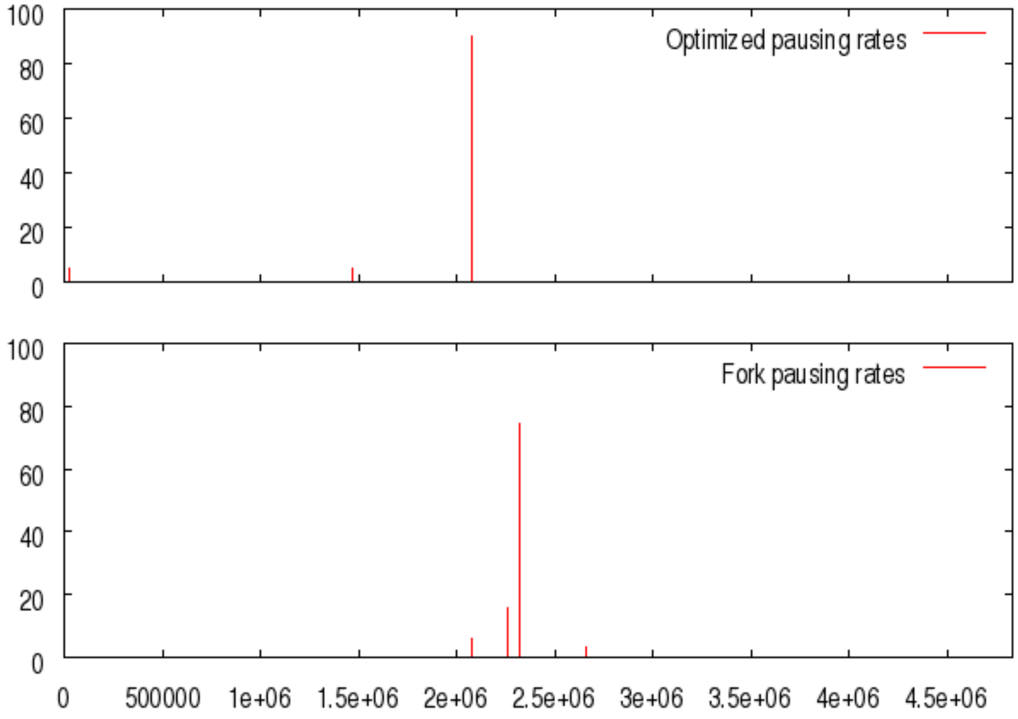

Escherichia coli UMN026

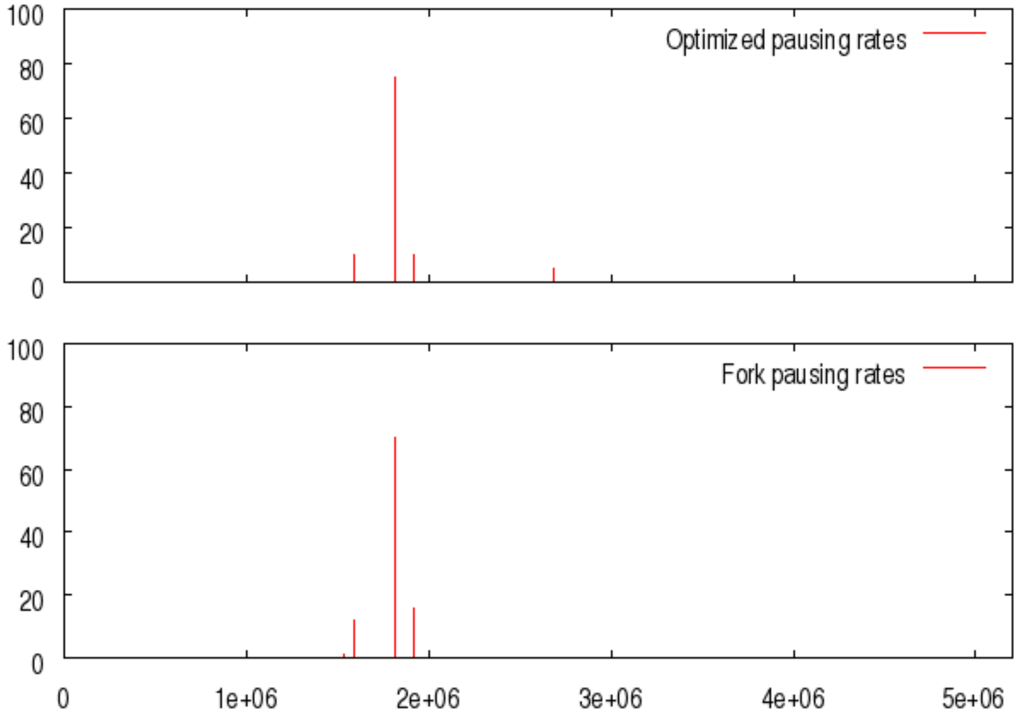

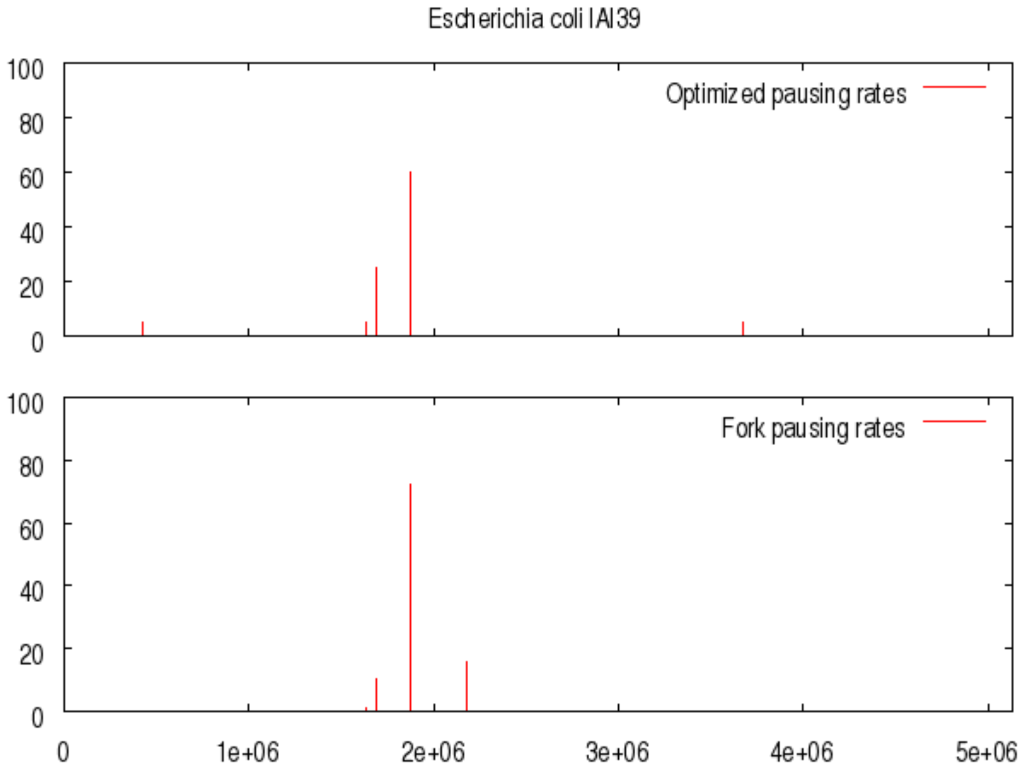
Escherichia coli IAI39
Optimized pausing rates
Fork pausing rates
100
80
60
40
20
0
0
1e+06
2e+06
3e+06
4e+06
5e+06

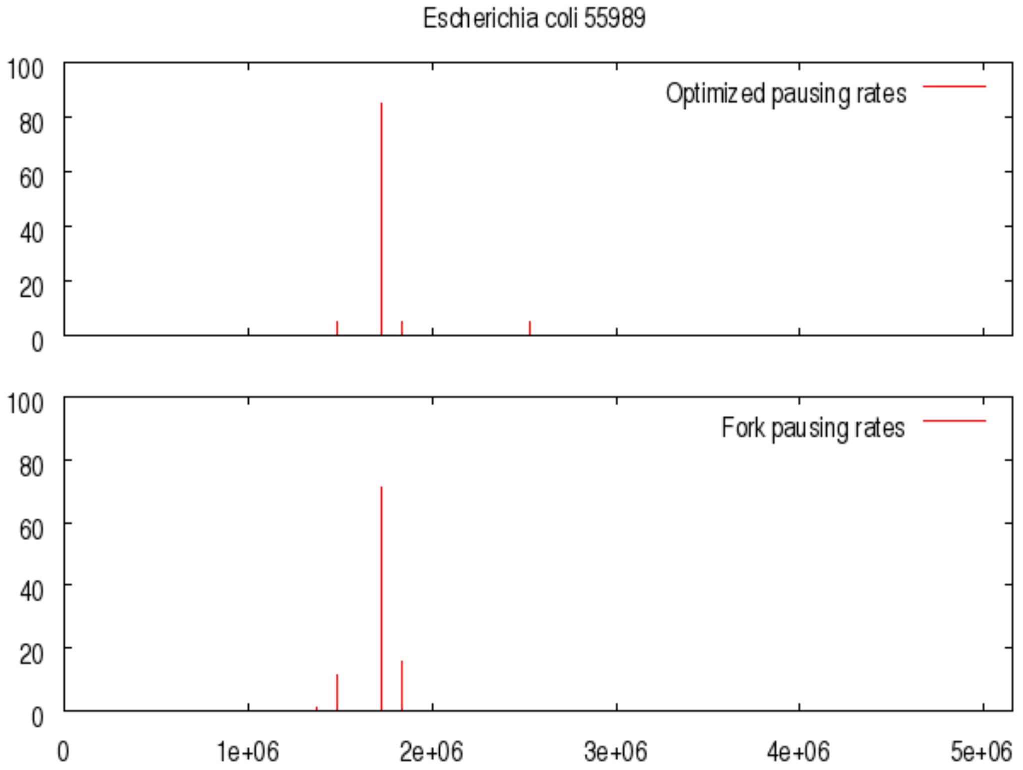
Escherichia coli 55989
Optimized pausing rates
Fork pausing rates
100
80
60
40
20
0
0
1e+06
2e+06
3e+06
4e+06
5e+06

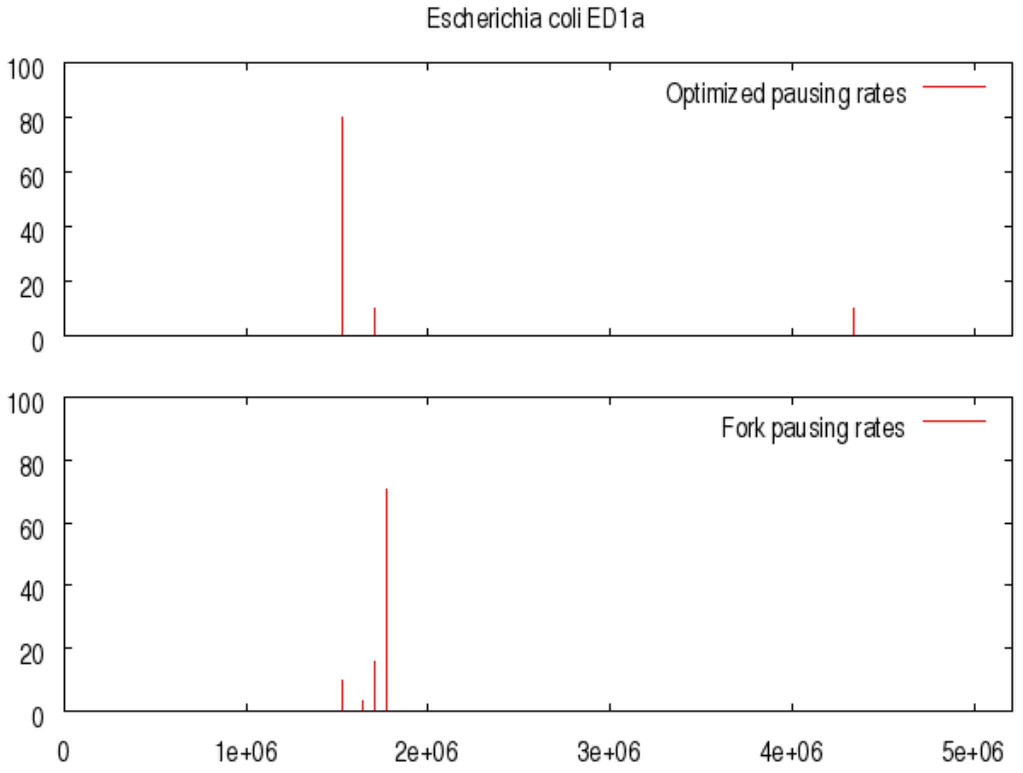
Escherichia coli ED1a
Optimized pausing rates
Fork pausing rates
100
80
60
40
20
0
1e+06
2e+06
3e+06
4e+06
5e+06

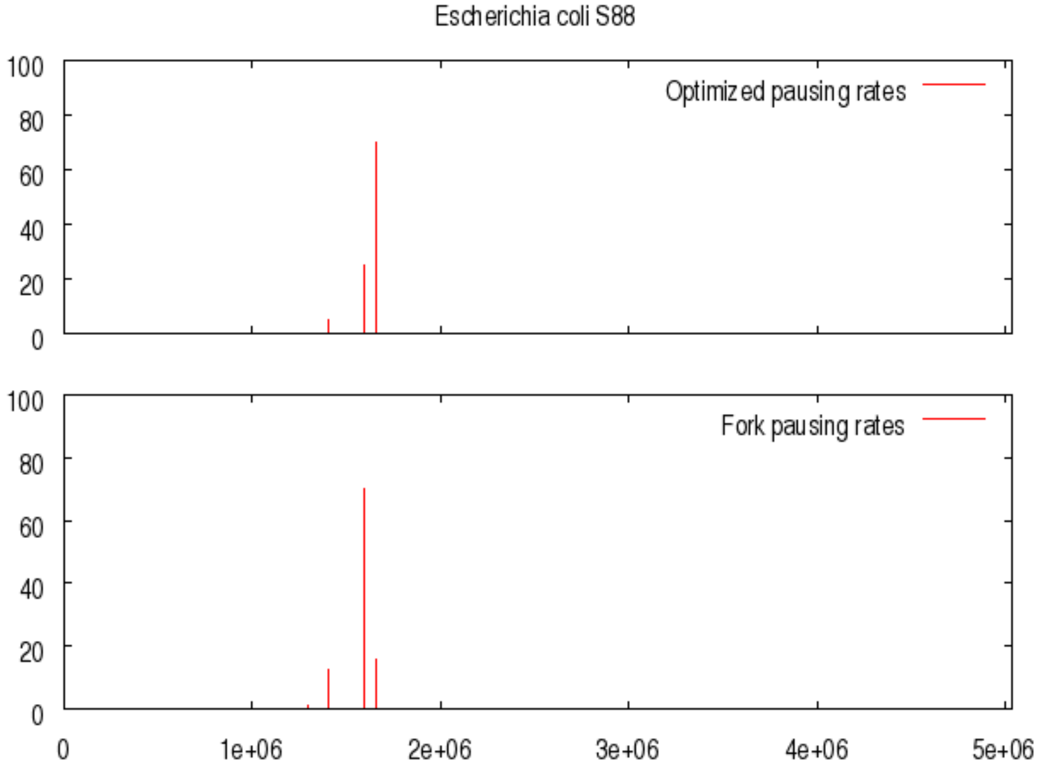
Escherichia coli S88
Optimized pausing rates
Fork pausing rates
100
80
60
40
20
0
1e+06
2e+06
3e+06
4e+06
5e+06

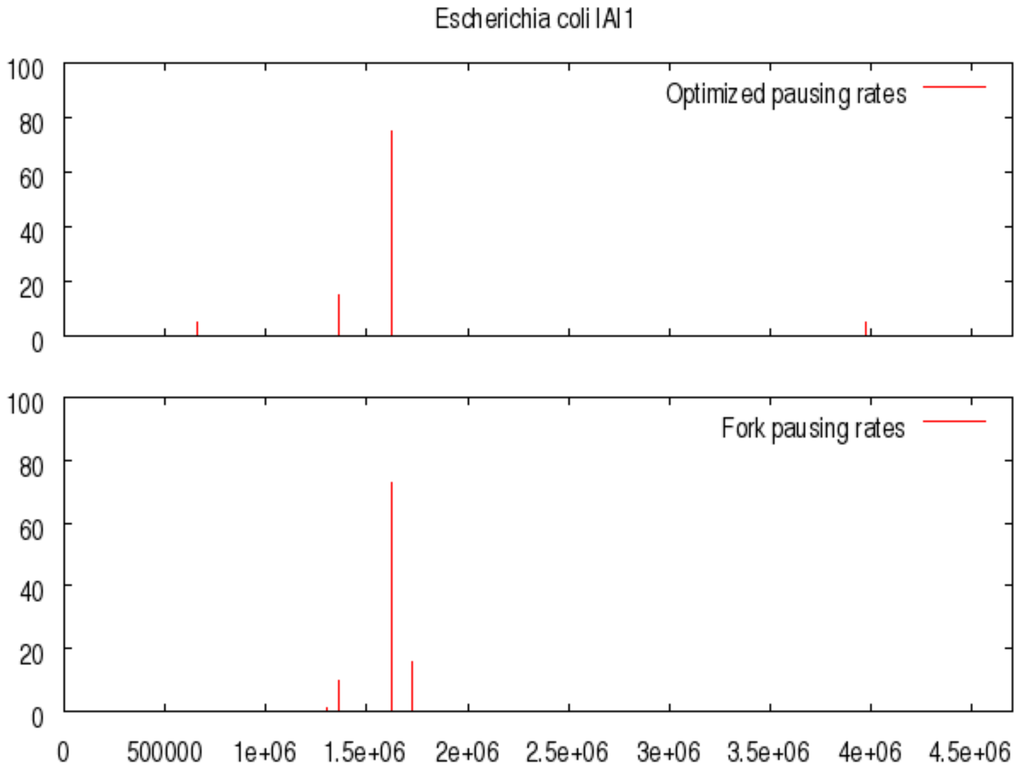
Escherichia coli IAI1
Optimized pausing rates
Fork pausing rates
100
80
60
40
20
0
0
500000
1e+06
1.5e+06
2e+06
2.5e+06
3e+06
3.5e+06
4e+06
4.5e+06

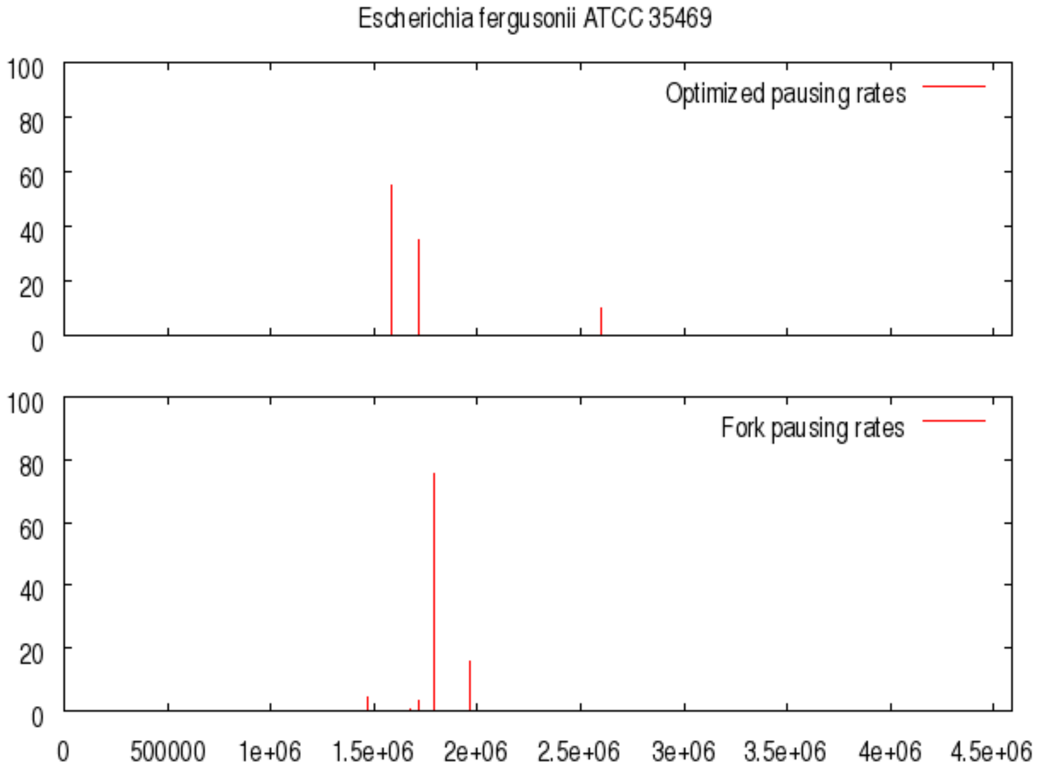
Escherichia fergusonii ATCC 35469
Optimized pausing rates
Fork pausing rates
100
80
60
40
20
0
0
500000
1e+06
1.5e+06
2e+06
2.5e+06
3e+06
3.5e+06
4e+06
4.5e+06

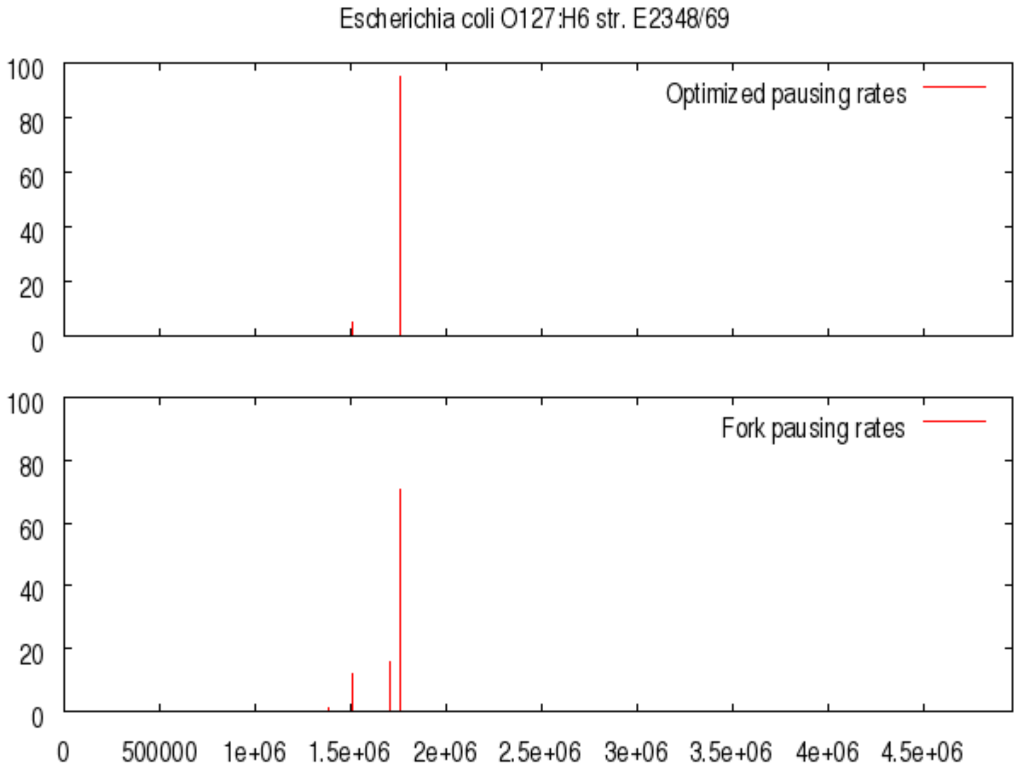
Escherichia coli O127:H6 str. E2348/69
Optimized pausing rates
Fork pausing rates
100
80
60
40
20
0
500000
1e+06
1.5e+06
2e+06
2.5e+06
3e+06
3.5e+06
4e+06
4.5e+06

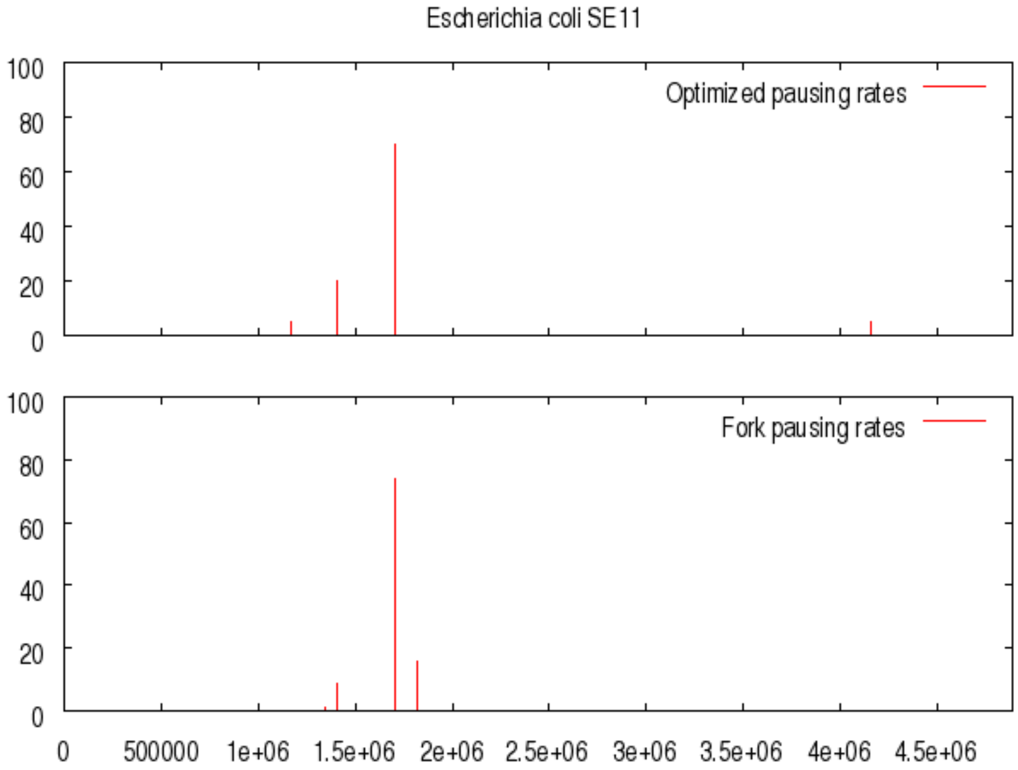
Escherichia coli SE11
Optimized pausing rates
Fork pausing rates
100
80
60
40
20
0
500000
1e+06
1.5e+06
2e+06
2.5e+06
3e+06
3.5e+06
4e+06
4.5e+06

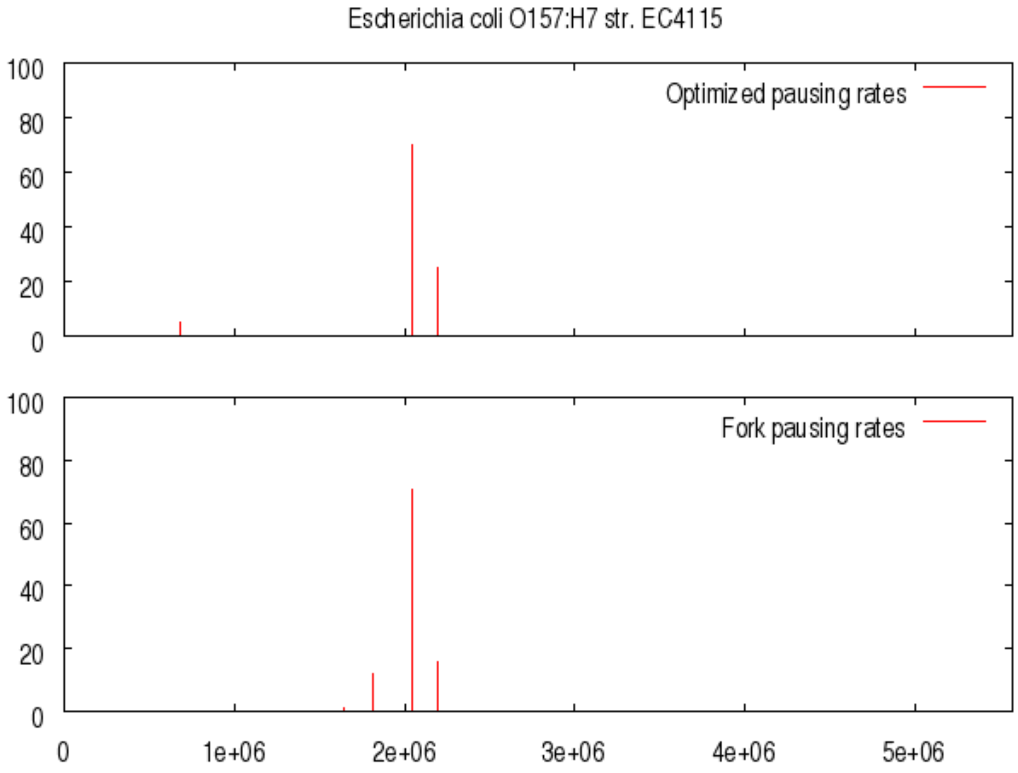
Escherichia coli O157:H7 str. EC4115
Optimized pausing rates
Fork pausing rates
100
80
60
40
20
0
1e+06
2e+06
3e+06
4e+06
5e+06

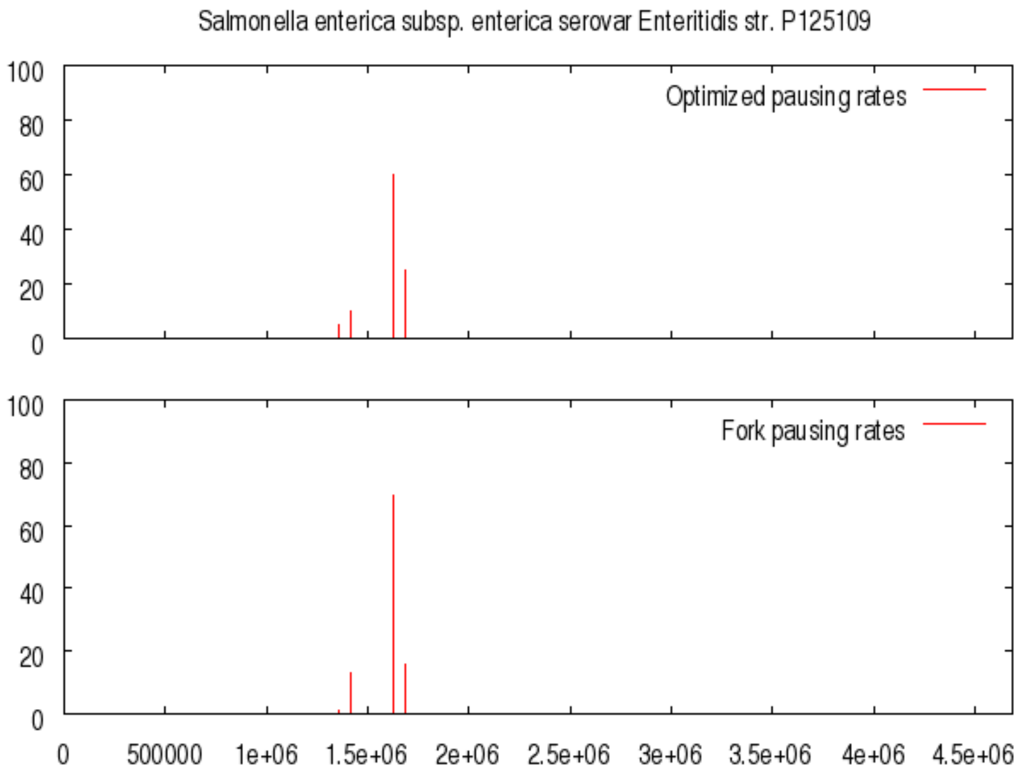
Salmonella enterica subsp. enterica serovar Enteritidis str. P125109
Optimized pausing rates
Fork pausing rates
100
80
60
40
20
0
500000
1e+06
1.5e+06
2e+06
2.5e+06
3e+06
3.5e+06
4e+06
4.5e+06

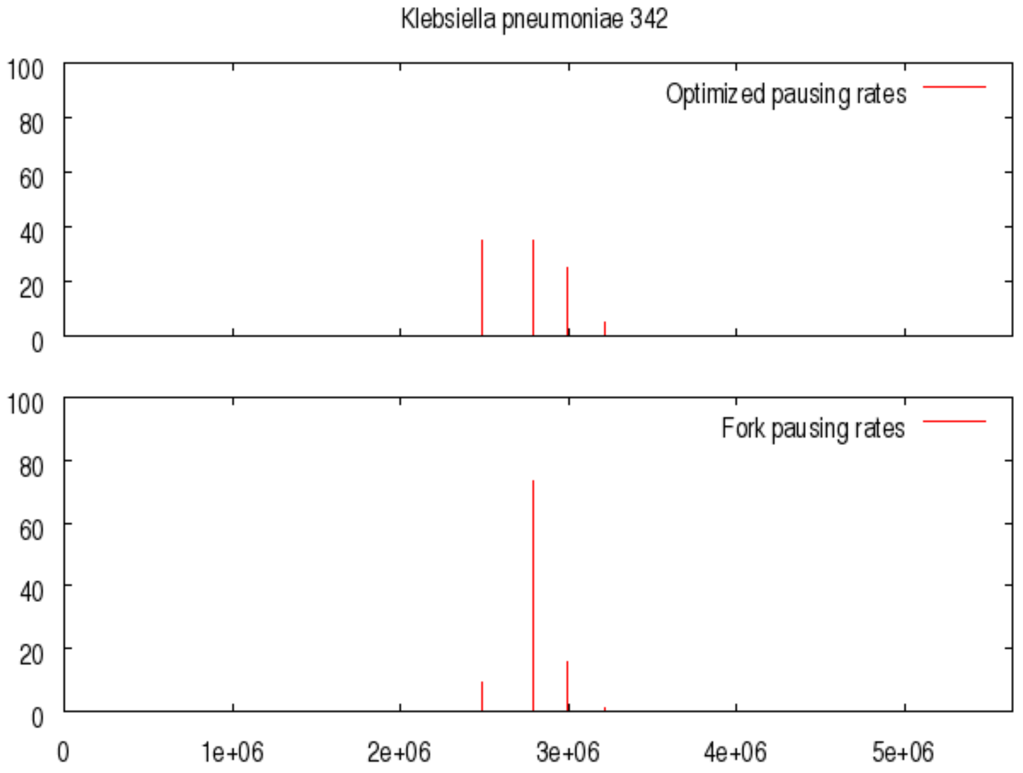
Klebsiella pneumoniae 342
Optimized pausing rates
Fork pausing rates
0
20
40
60
80
100
0
1e+06
2e+06
3e+06
4e+06
5e+06

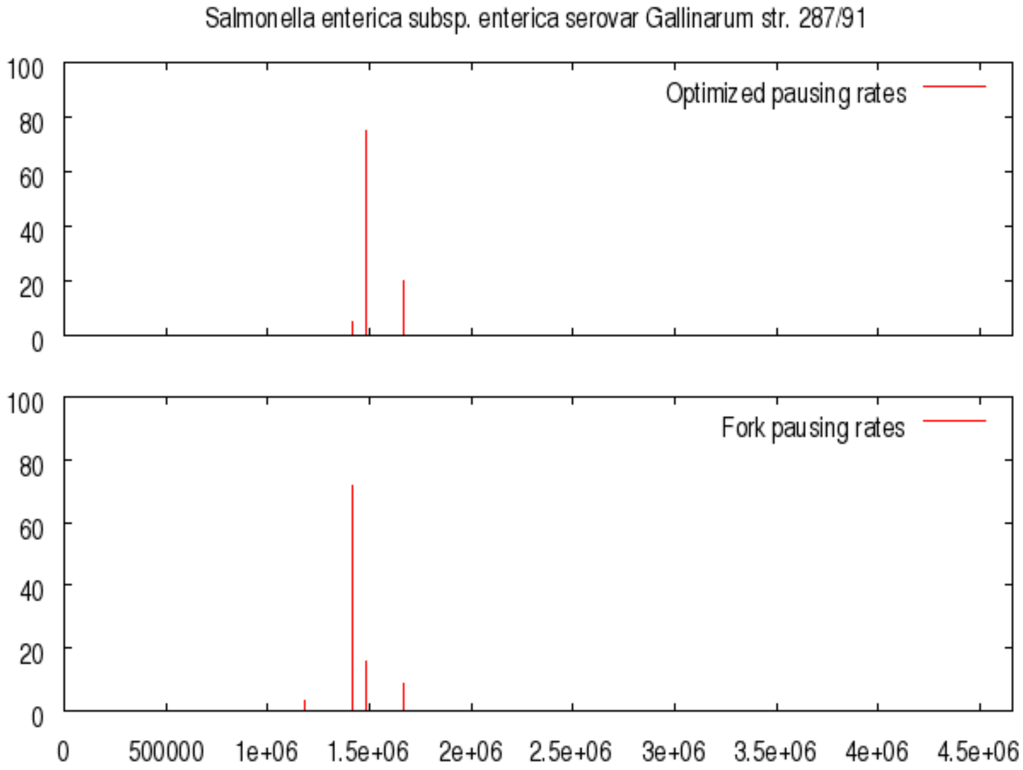
Salmonella enterica subsp. enterica serovar Gallinarum str. 287/91
Optimized pausing rates
Fork pausing rates
0
20
40
60
80
100
0
500000
1e+06
1.5e+06
2e+06
2.5e+06
3e+06
3.5e+06
4e+06
4.5e+06

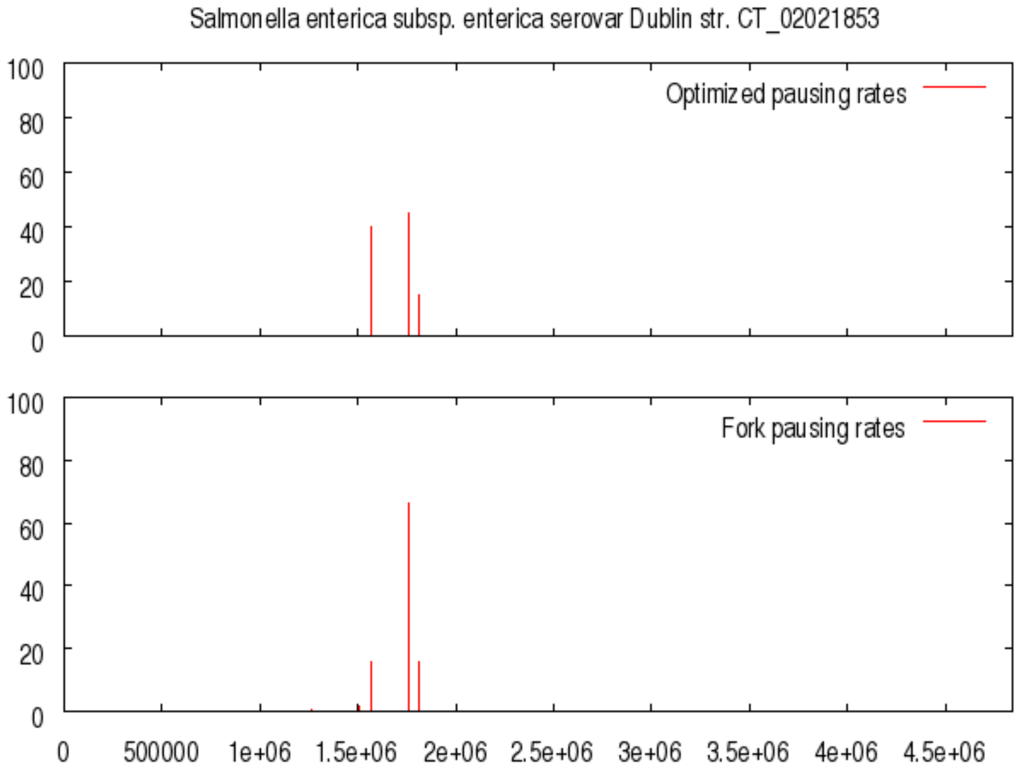
Salmonella enterica subsp. enterica serovar Dublin str. CT_02021853
Optimized pausing rates
Fork pausing rates
100
80
60
40
20
0
0
500000
1e+06
1.5e+06
2e+06
2.5e+06
3e+06
3.5e+06
4e+06
4.5e+06

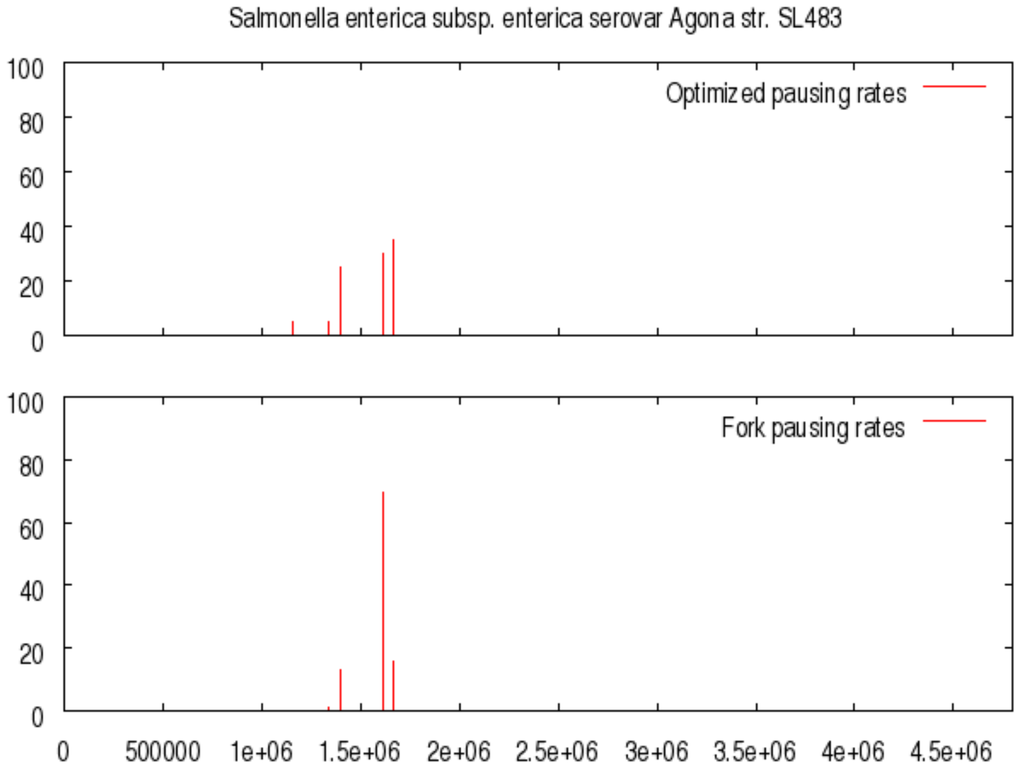
Salmonella enterica subsp. enterica serovar Agona str. SL483
Optimized pausing rates
Fork pausing rates
100
80
60
40
20
0
0
500000
1e+06
1.5e+06
2e+06
2.5e+06
3e+06
3.5e+06
4e+06
4.5e+06

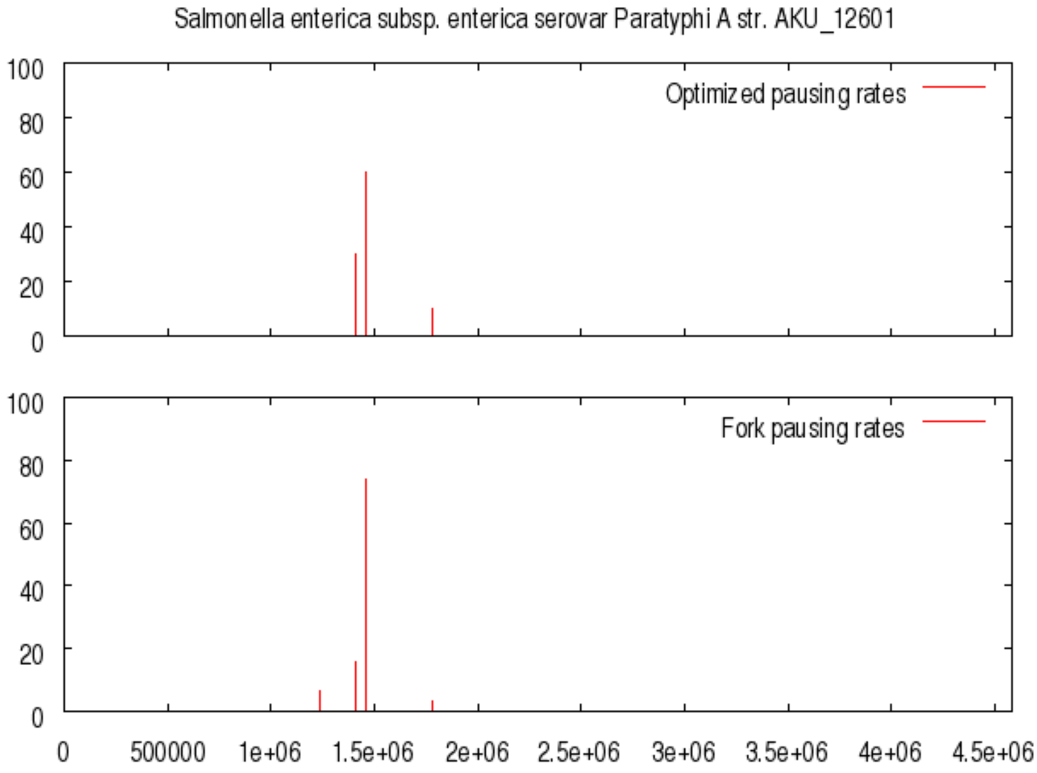
Salmonella enterica subsp. enterica serovar Paratyphi A str. AKU_12601
Optimized pausing rates
Fork pausing rates
0
20
40
60
80
100
0
500000
1e+06
1.5e+06
2e+06
2.5e+06
3e+06
3.5e+06
4e+06
4.5e+06

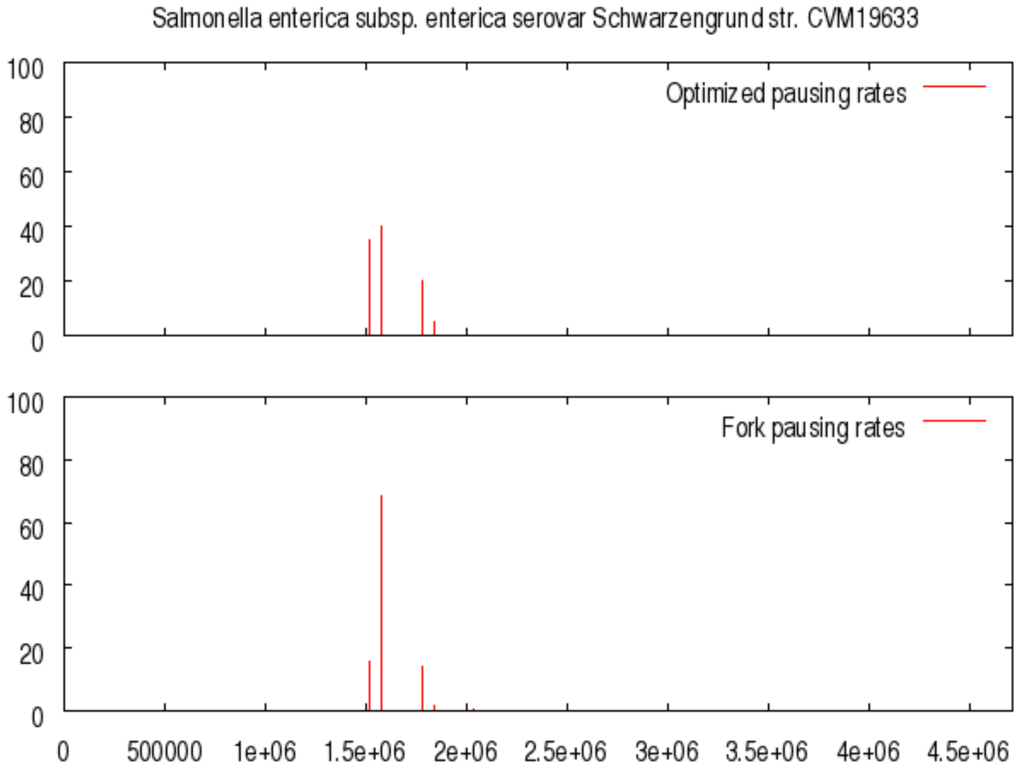
Salmonella enterica subsp. enterica serovar Schwarzengrund str. CVM19633
Optimized pausing rates
Fork pausing rates
0
20
40
60
80
100
0
500000
1e+06
1.5e+06
2e+06
2.5e+06
3e+06
3.5e+06
4e+06
4.5e+06

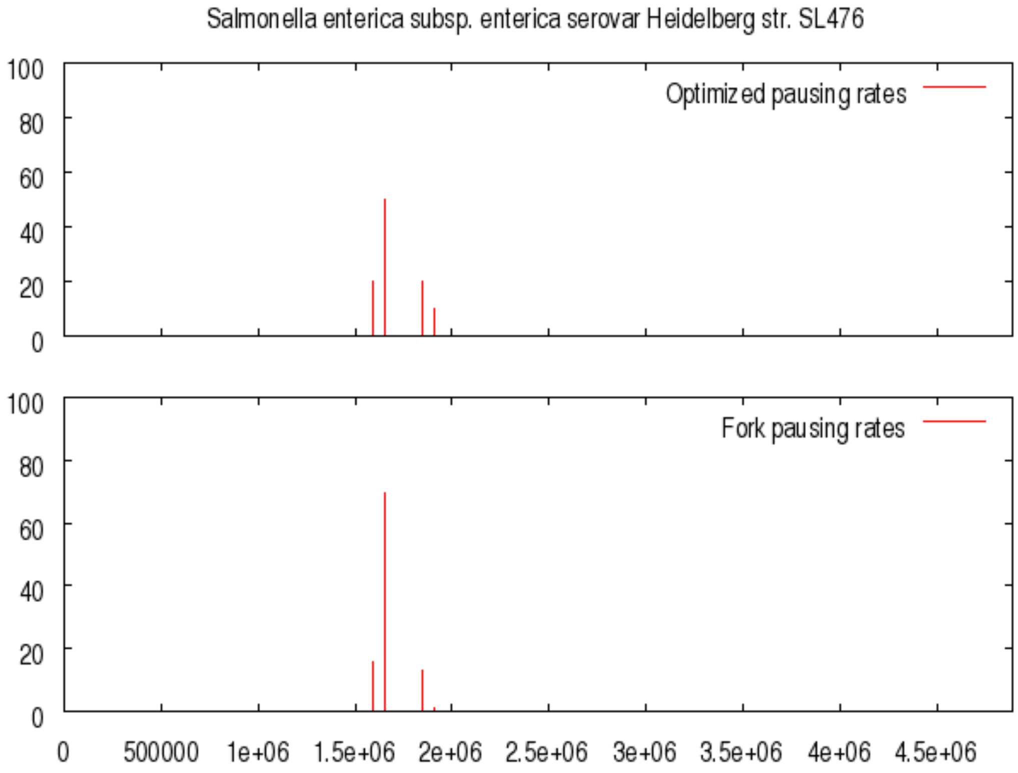
Salmonella enterica subsp. enterica serovar Heidelberg str. SL476
Optimized pausing rates
Fork pausing rates
100
80
60
40
20
0
0
500000
1e+06
1.5e+06
2e+06
2.5e+06
3e+06
3.5e+06
4e+06
4.5e+06

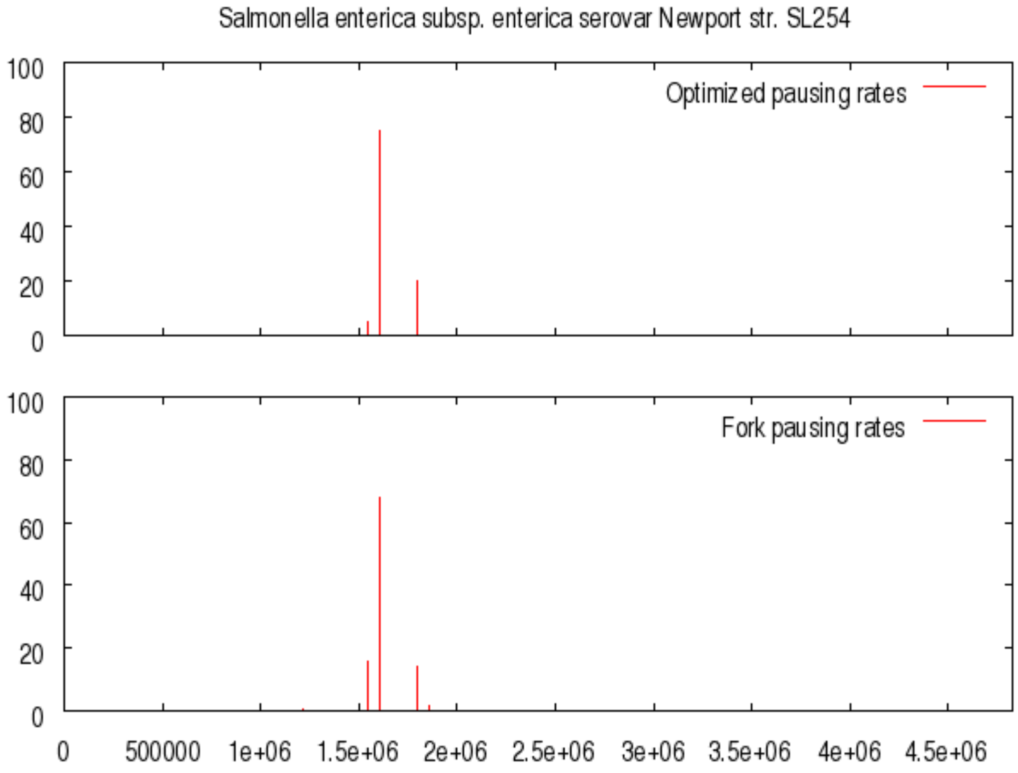
Salmonella enterica subsp. enterica serovar Newport str. SL254
Optimized pausing rates
Fork pausing rates
100
80
60
40
20
0
0
500000
1e+06
1.5e+06
2e+06
2.5e+06
3e+06
3.5e+06
4e+06
4.5e+06

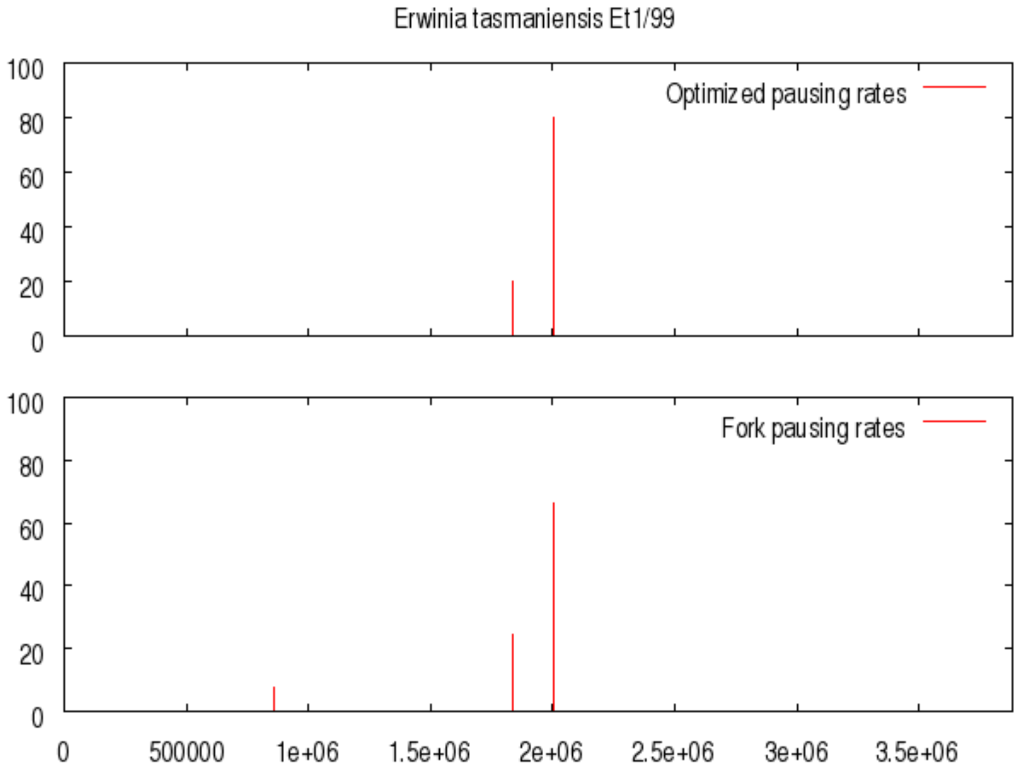
Erwinia tasmaniensis Et1/99
Optimized pausing rates
Fork pausing rates
0
20
40
60
80
100
0
500000
1e+06
1.5e+06
2e+06
2.5e+06
3e+06
3.5e+06

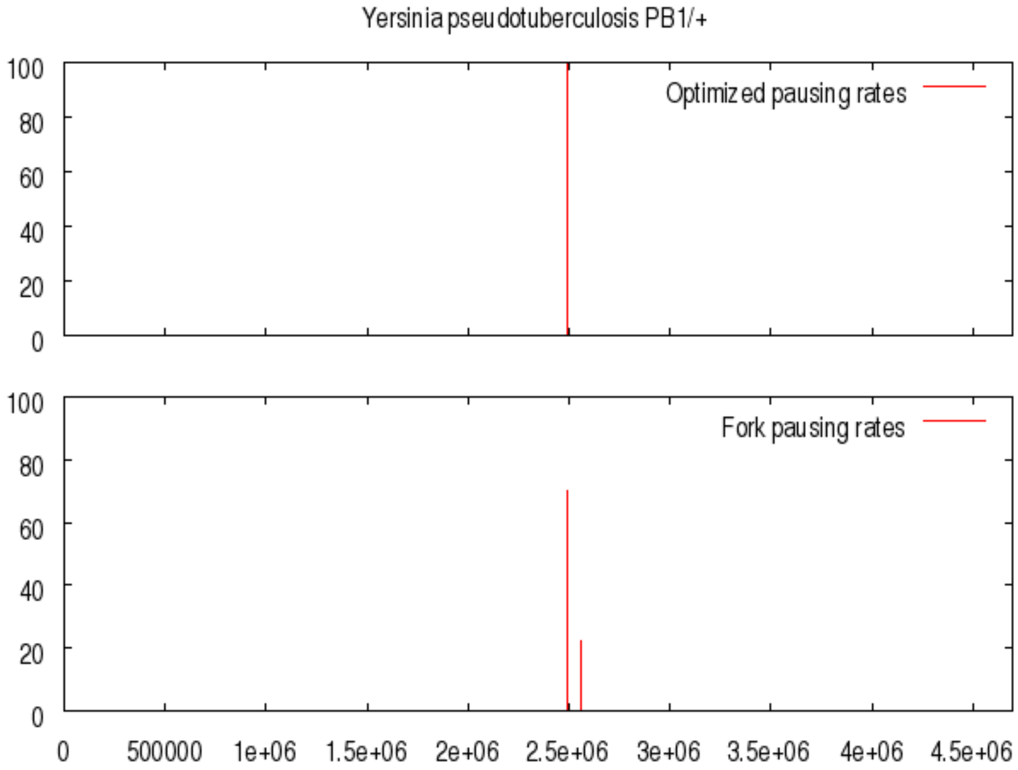
Yersinia pseudotuberculosis PB1/+
Optimized pausing rates
Fork pausing rates
0
20
40
60
80
100
0
500000
1e+06
1.5e+06
2e+06
2.5e+06
3e+06
3.5e+06
4e+06
4.5e+06

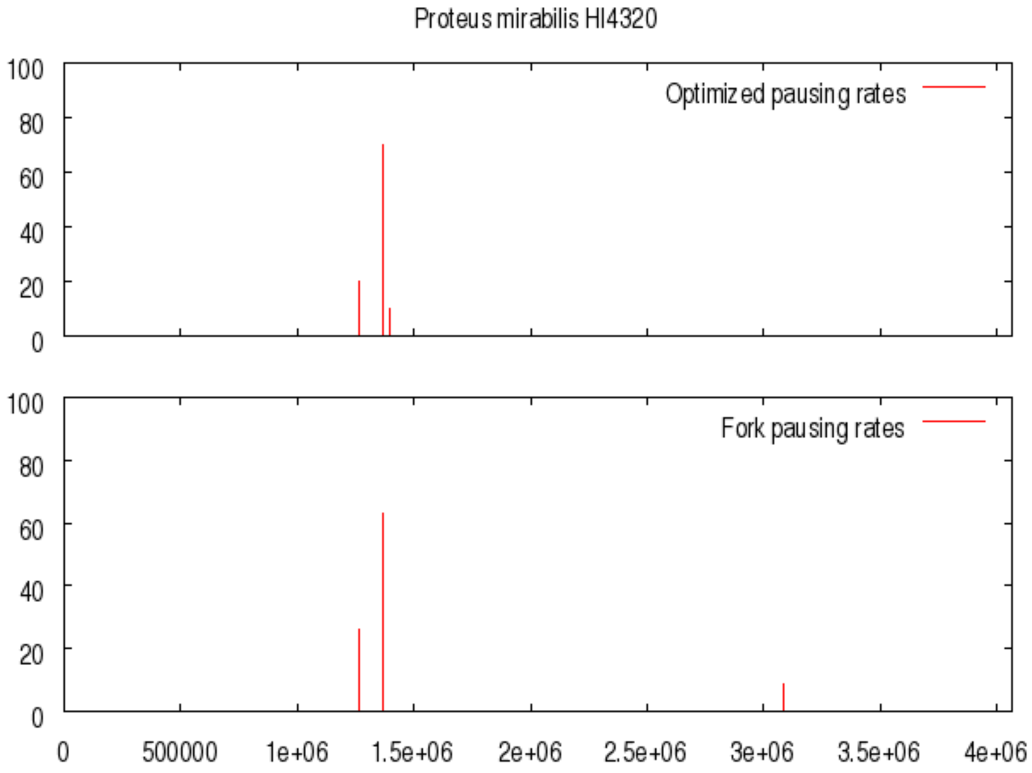
Proteus mirabilis HI4320
Optimized pausing rates
Fork pausing rates
100
80
60
40
20
0
0
500000
1e+06
1.5e+06
2e+06
2.5e+06
3e+06
3.5e+06
4e+06

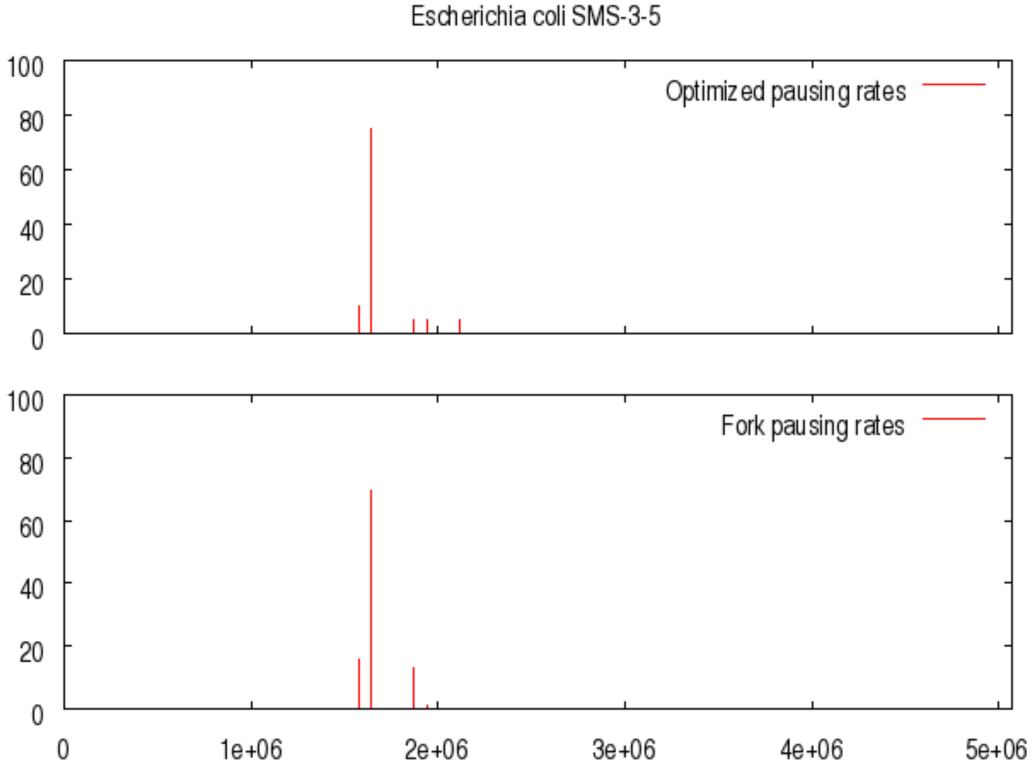
Escherichia coli SMS-3-5
Optimized pausing rates
Fork pausing rates
100
80
60
40
20
0
0
1e+06
2e+06
3e+06
4e+06
5e+06

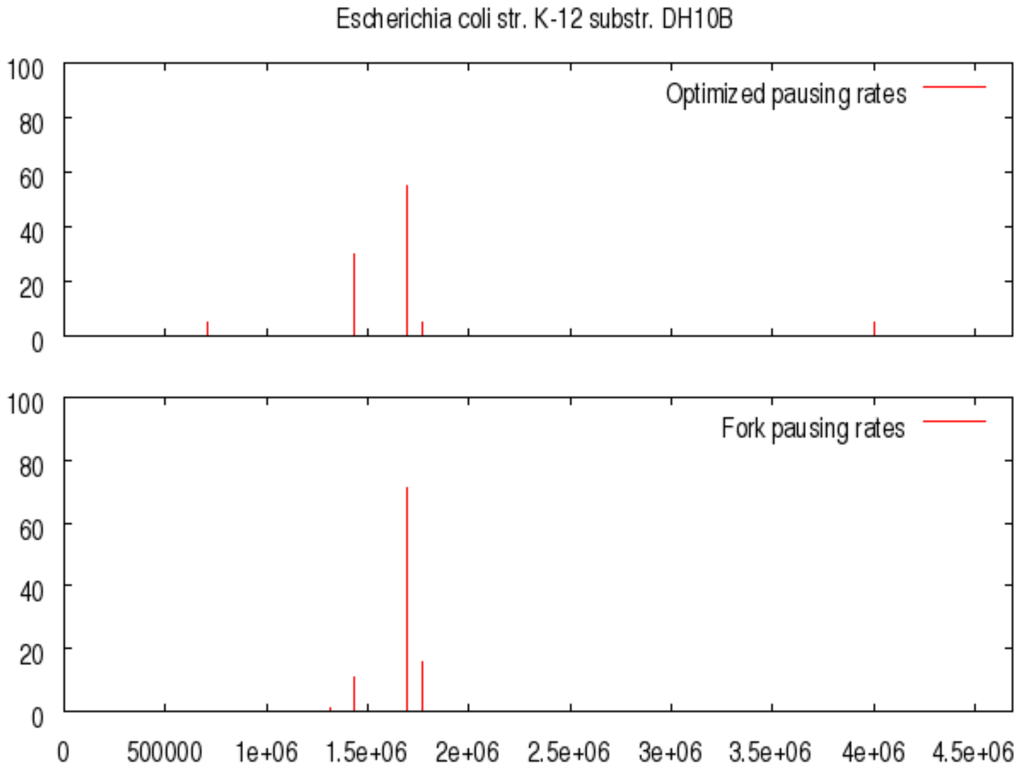
Escherichia coli str. K-12 substr. DH10B
Optimized pausing rates
Fork pausing rates
100
80
60
40
20
0
0
500000
1e+06
1.5e+06
2e+06
2.5e+06
3e+06
3.5e+06
4e+06
4.5e+06

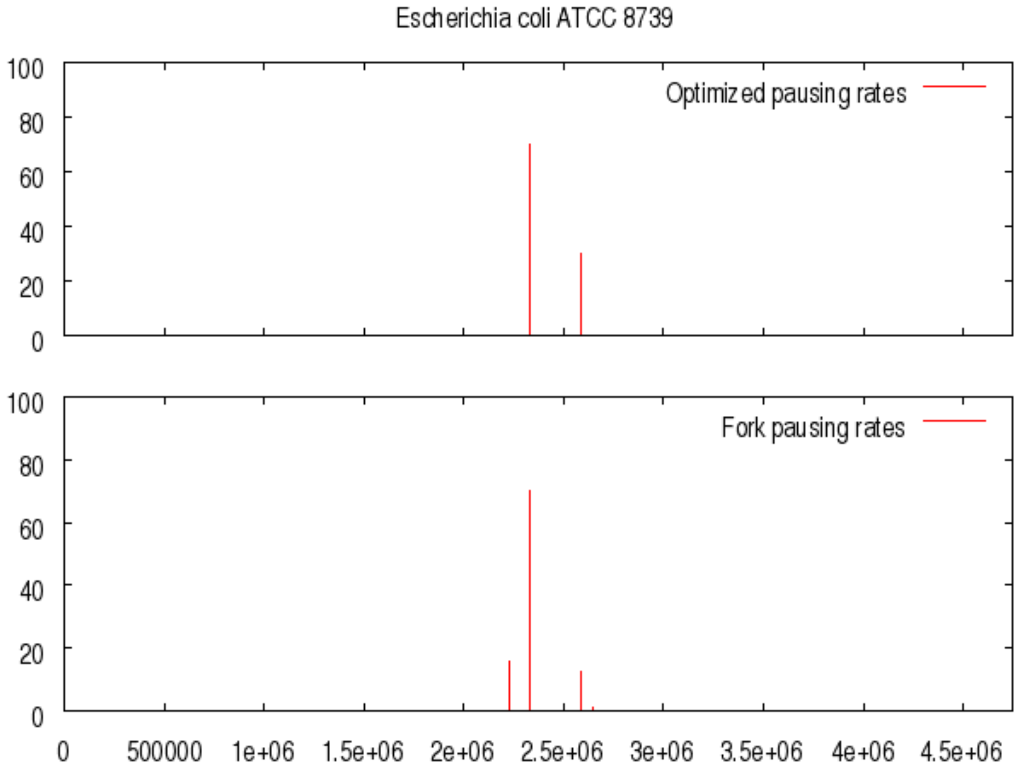
Escherichia coli ATCC 8739
Optimized pausing rates
Fork pausing rates
100
80
60
40
20
0
0
500000
1e+06
1.5e+06
2e+06
2.5e+06
3e+06
3.5e+06
4e+06
4.5e+06

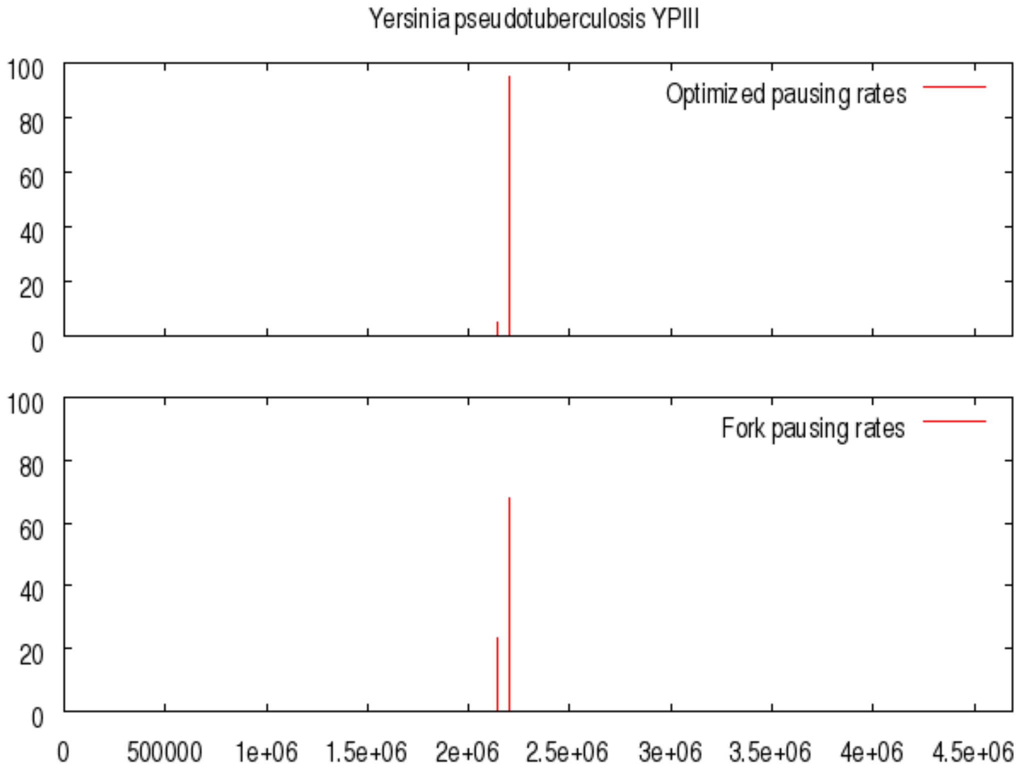
Yersinia pseudotuberculosis YPIII
Optimized pausing rates
Fork pausing rates
100
80
60
40
20
0
500000
1e+06
1.5e+06
2e+06
2.5e+06
3e+06
3.5e+06
4e+06
4.5e+06

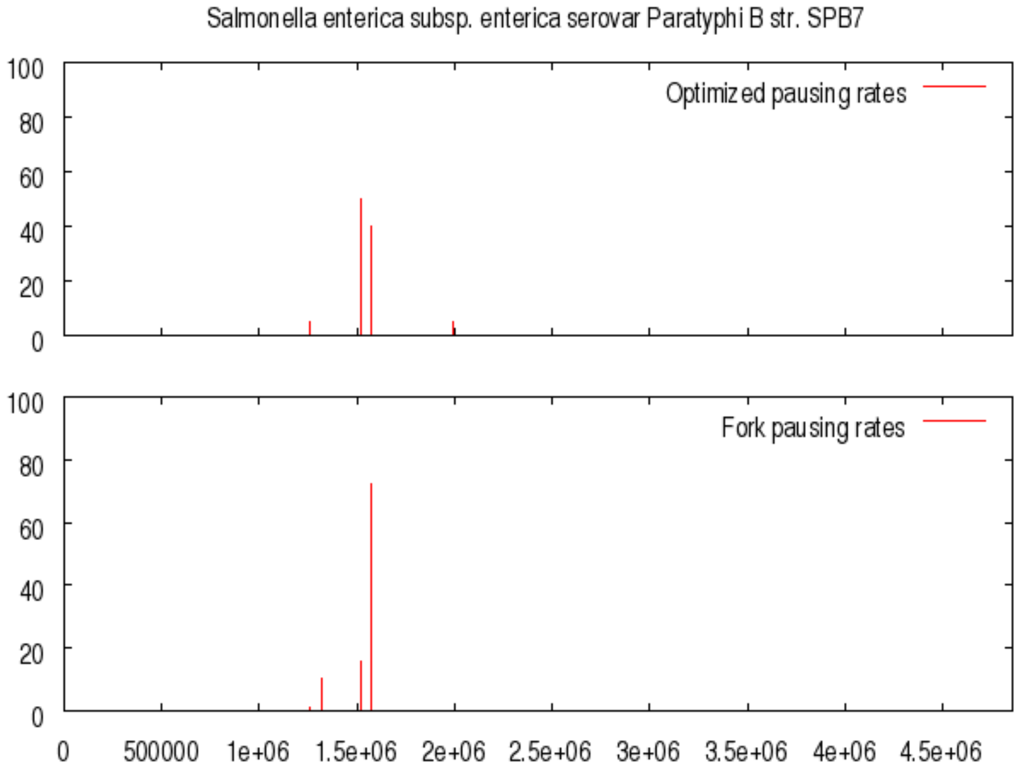
Salmonella enterica subsp. enterica serovar Paratyphi B str. SPB7
Optimized pausing rates
Fork pausing rates
100
80
60
40
20
0
500000
1e+06
1.5e+06
2e+06
2.5e+06
3e+06
3.5e+06
4e+06
4.5e+06

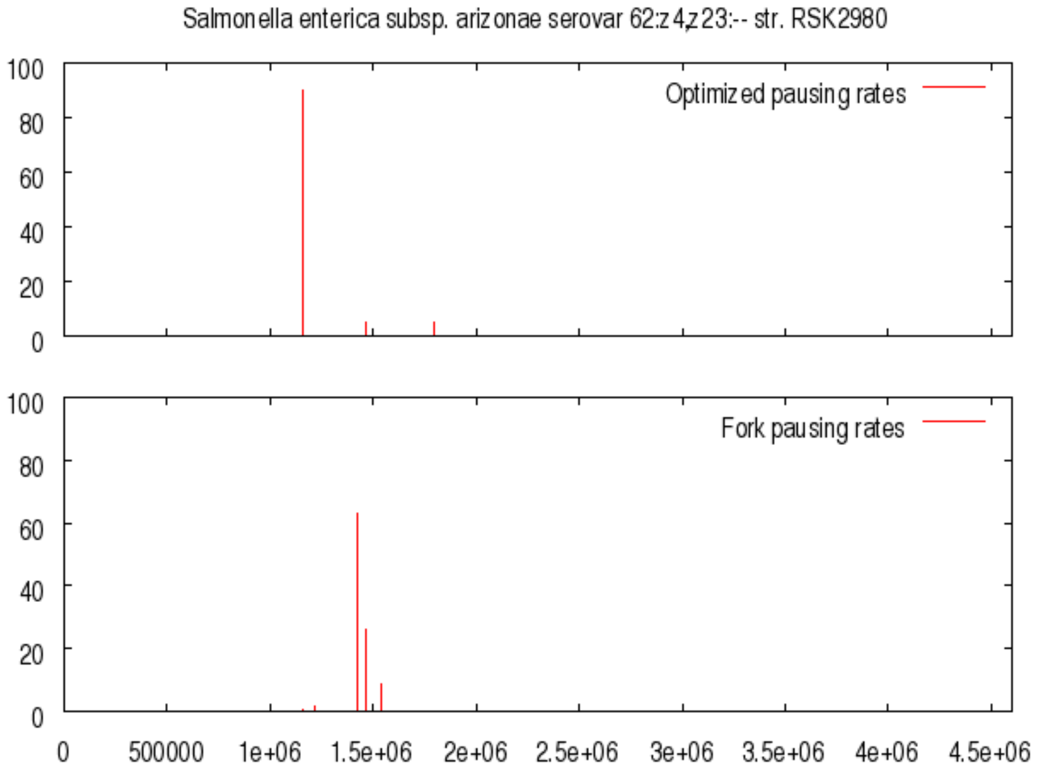

Salmonella enterica subsp. arizonae serovar 62:z4,z23:-- str. RSK2980
Optimized pausing rates
Fork pausing rates
100
80
60
40
20
0
0
500000
1e+06
1.5e+06
2e+06
2.5e+06
3e+06
3.5e+06
4e+06
4.5e+06

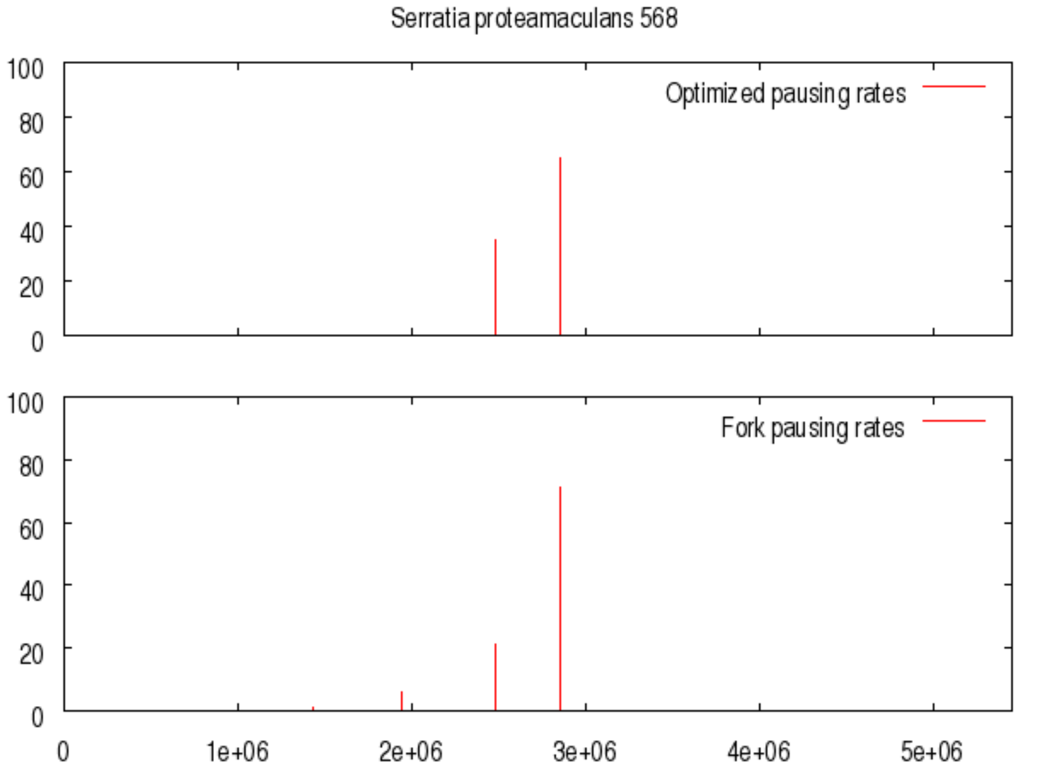

Serratia proteamaculans 568
Optimized pausing rates
Fork pausing rates
100
80
60
40
20
0
0
1e+06
2e+06
3e+06
4e+06
5e+06

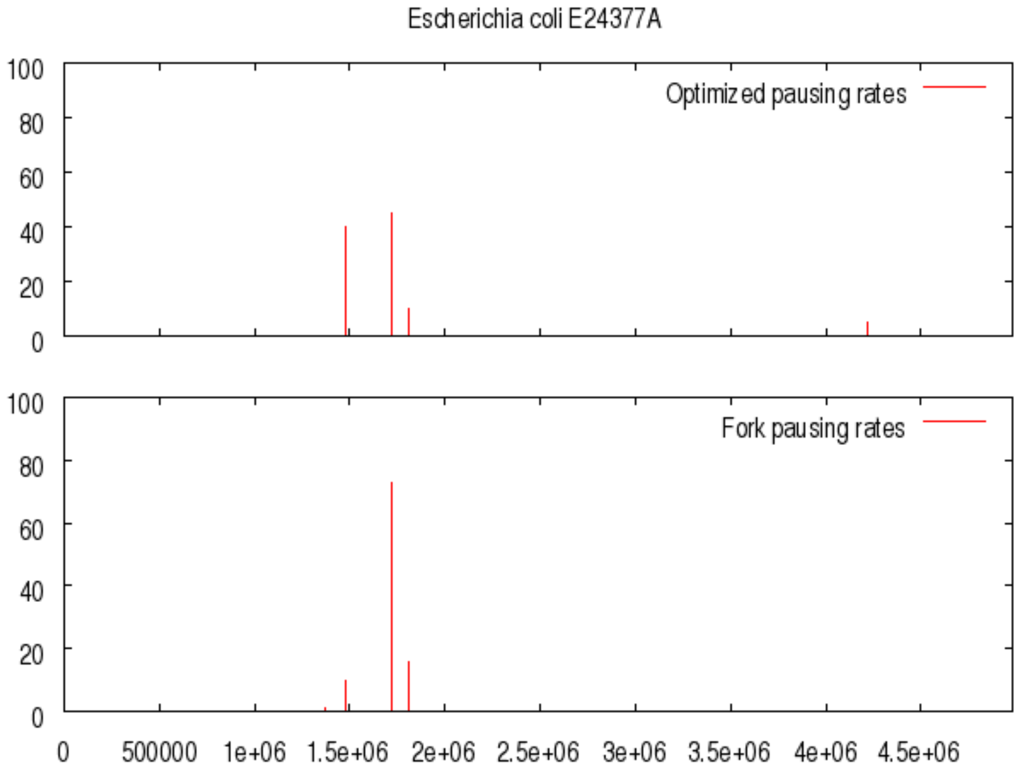
Escherichia coli E24377A
Optimized pausing rates
Fork pausing rates
100
80
60
40
20
0
0
500000
1e+06
1.5e+06
2e+06
2.5e+06
3e+06
3.5e+06
4e+06
4.5e+06

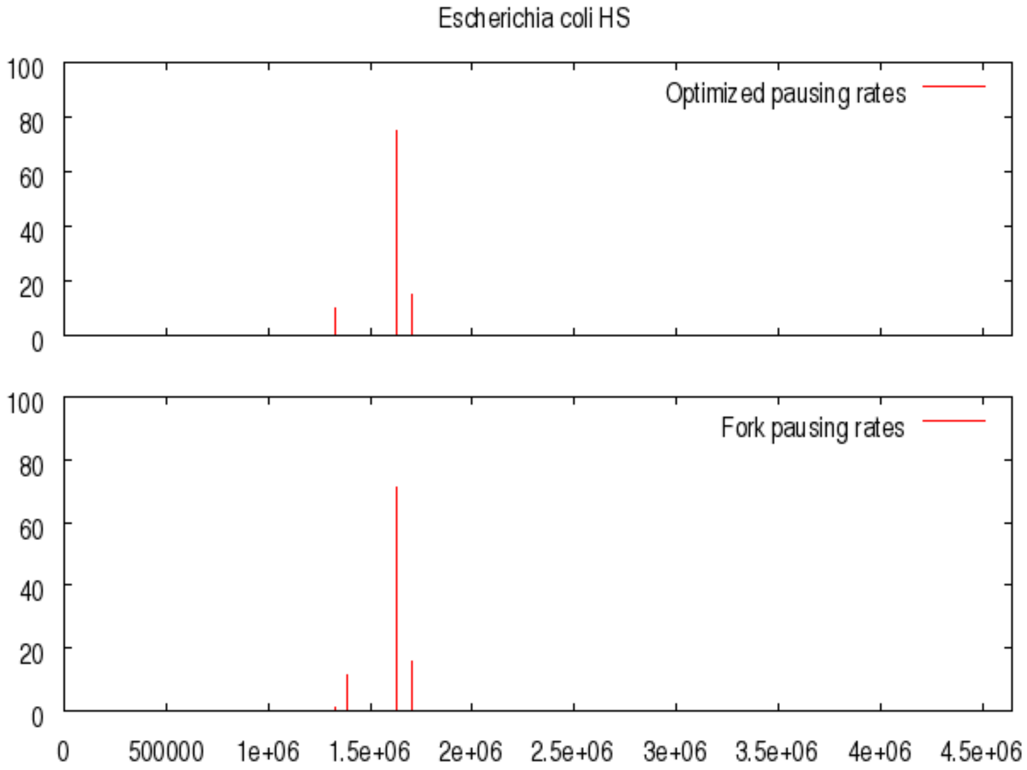
Escherichia coli HS
Optimized pausing rates
Fork pausing rates
100
80
60
40
20
0
0
500000
1e+06
1.5e+06
2e+06
2.5e+06
3e+06
3.5e+06
4e+06
4.5e+06

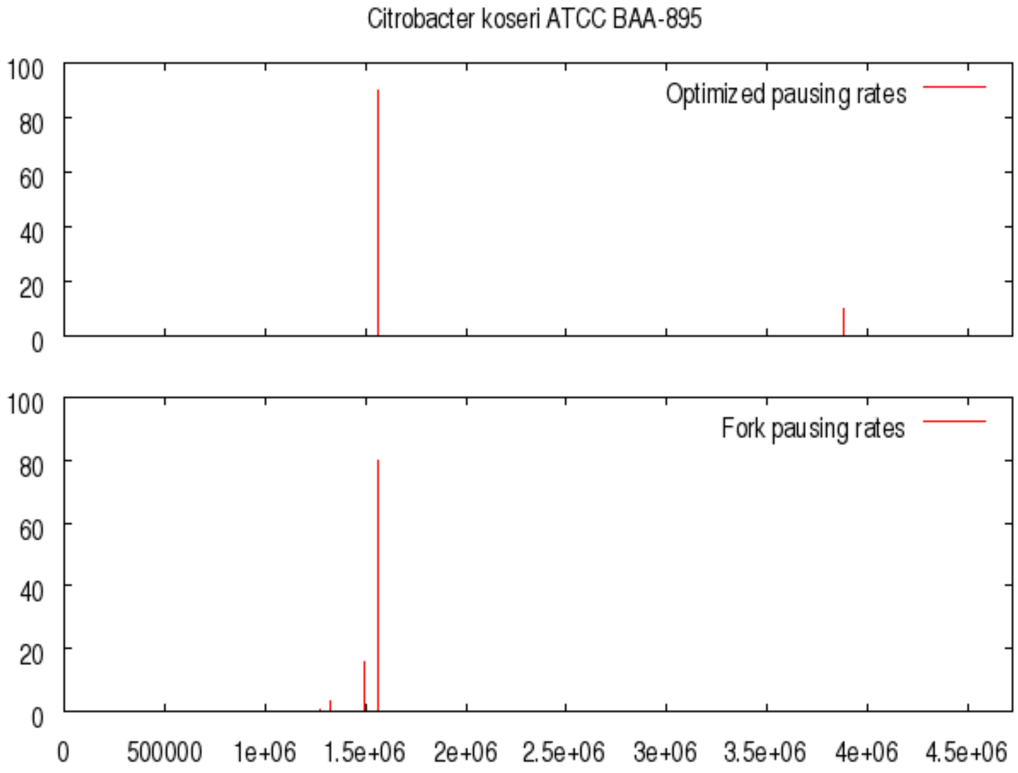
Citrobacter koseri ATCC BAA-895
Optimized pausing rates
Fork pausing rates
100
80
60
40
20
0
500000
1e+06
1.5e+06
2e+06
2.5e+06
3e+06
3.5e+06
4e+06
4.5e+06

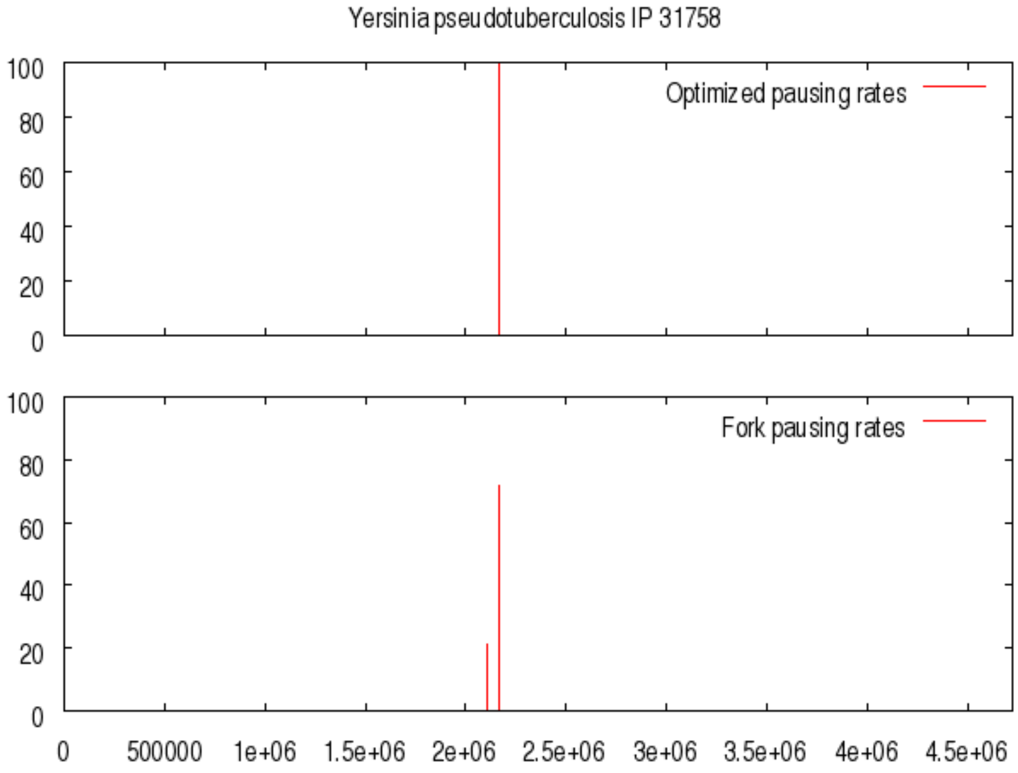
Yersinia pseudotuberculosis IP 31758
Optimized pausing rates
Fork pausing rates
100
80
60
40
20
0
500000
1e+06
1.5e+06
2e+06
2.5e+06
3e+06
3.5e+06
4e+06
4.5e+06

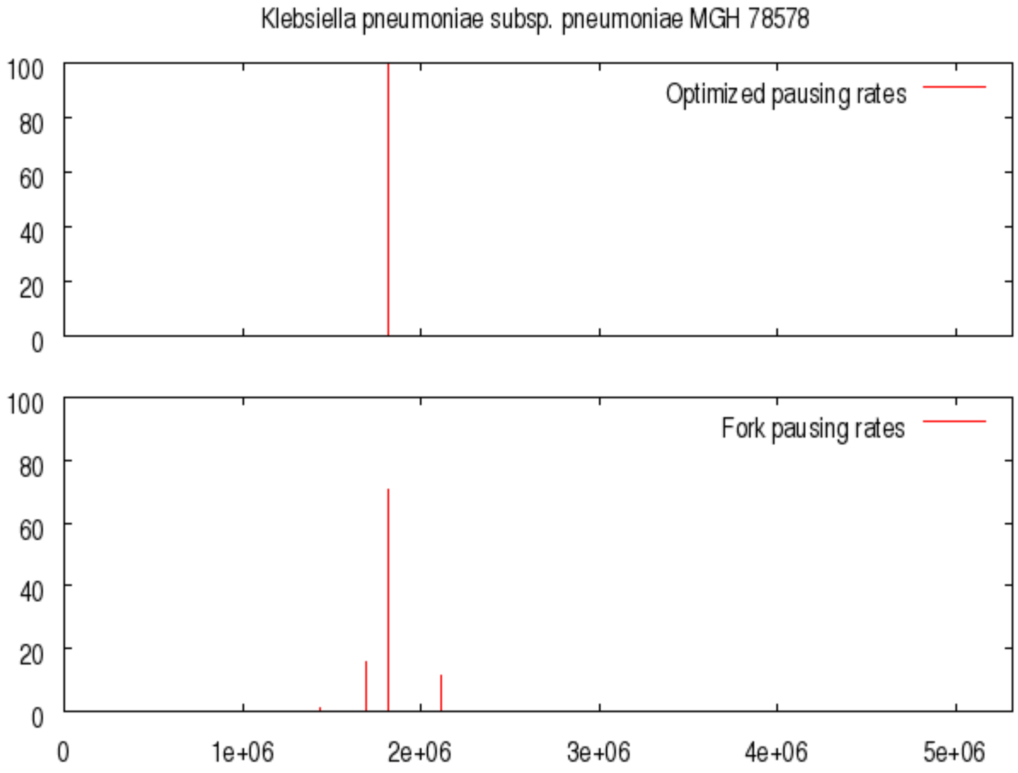
Klebsiella pneumoniae subsp. pneumoniae MGH 78578
Optimized pausing rates
Fork pausing rates
0
20
40
60
80
100
0
1e+06
2e+06
3e+06
4e+06
5e+06

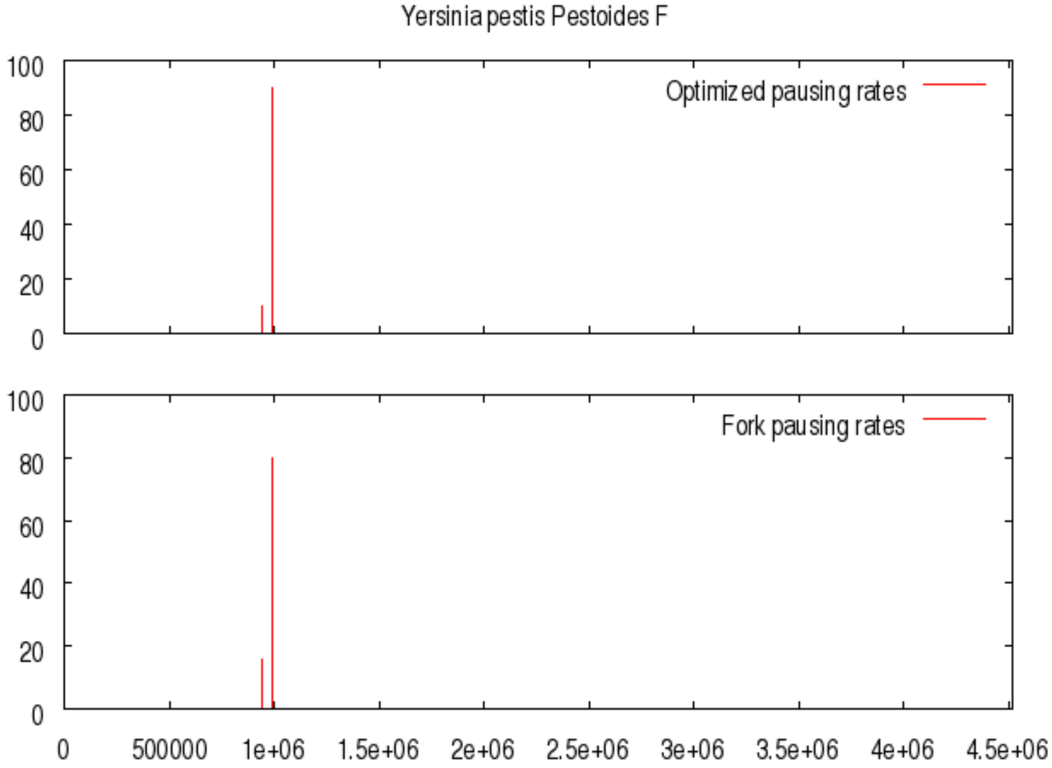
Yersinia pestis Pestoides F
Optimized pausing rates
Fork pausing rates
0
20
40
60
80
100
0
500000
1e+06
1.5e+06
2e+06
2.5e+06
3e+06
3.5e+06
4e+06
4.5e+06

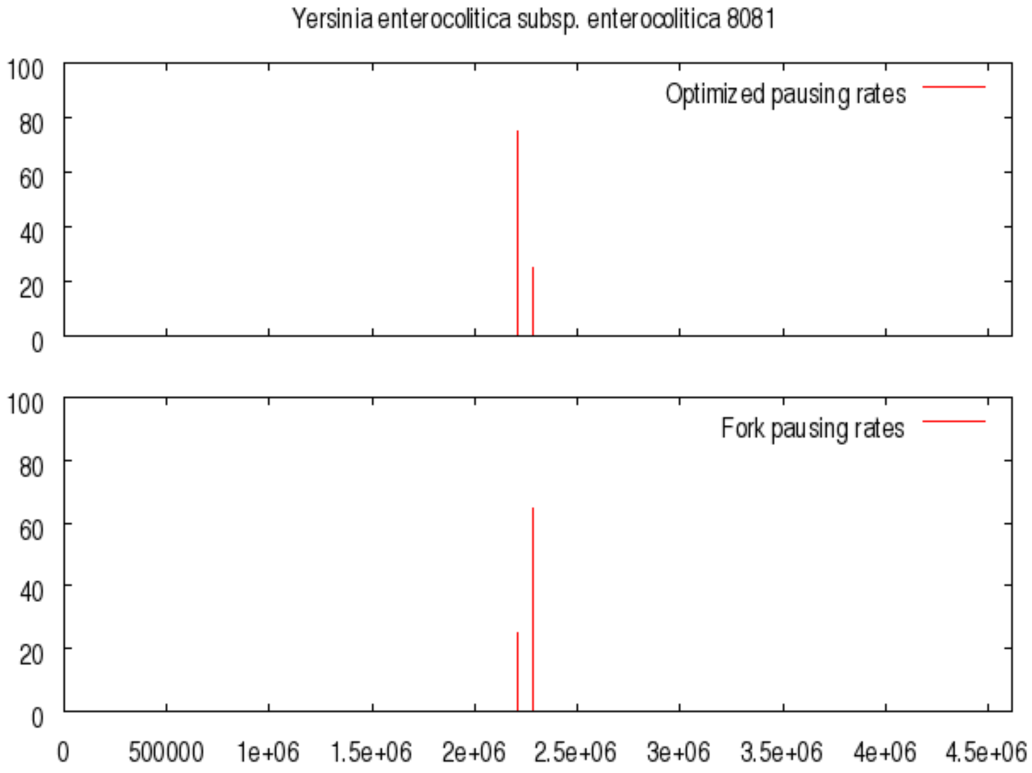
Yersinia enterocolitica subsp. enterocolitica 8081
Optimized pausing rates
Fork pausing rates
100
80
60
40
20
0
0
500000
1e+06
1.5e+06
2e+06
2.5e+06
3e+06
3.5e+06
4e+06
4.5e+06

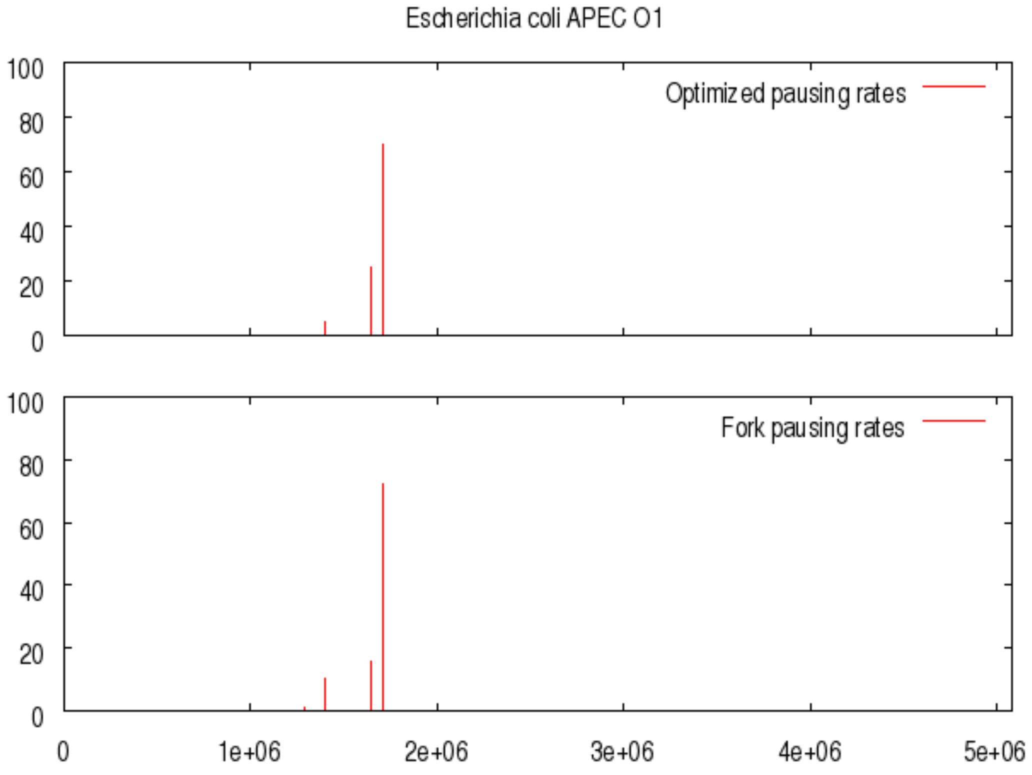
Escherichia coli APEC O1
Optimized pausing rates
Fork pausing rates
100
80
60
40
20
0
0
1e+06
2e+06
3e+06
4e+06
5e+06

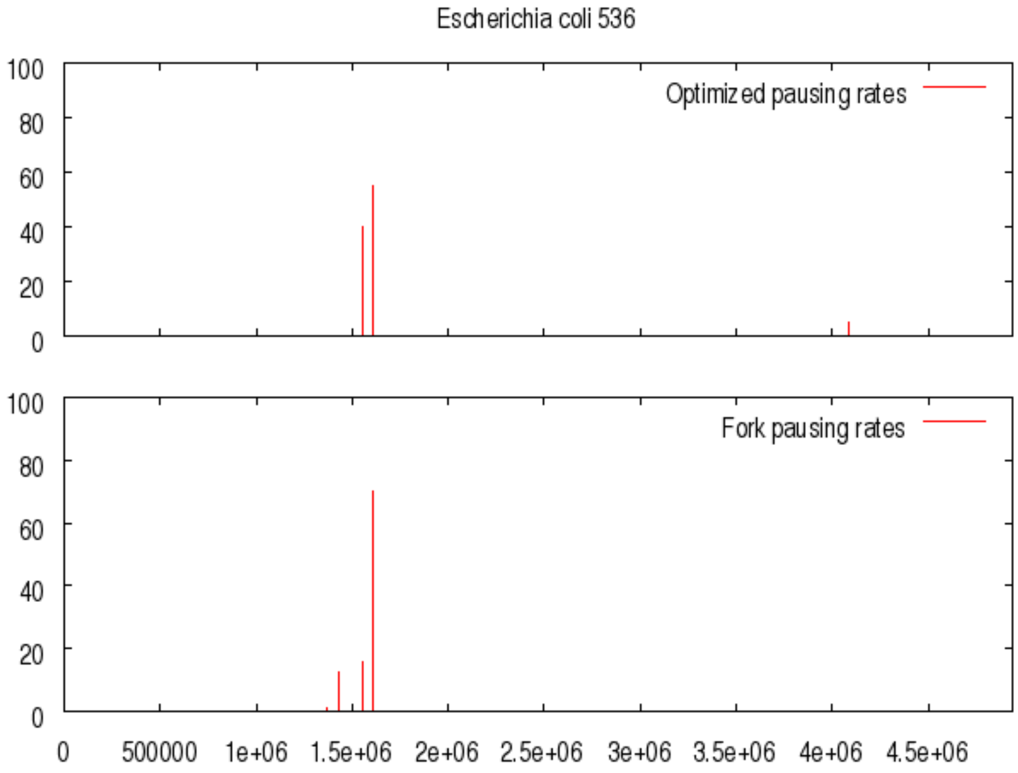
Escherichia coli 536
Optimized pausing rates
Fork pausing rates
0
20
40
60
80
100
0
500000
1e+06
1.5e+06
2e+06
2.5e+06
3e+06
3.5e+06
4e+06
4.5e+06

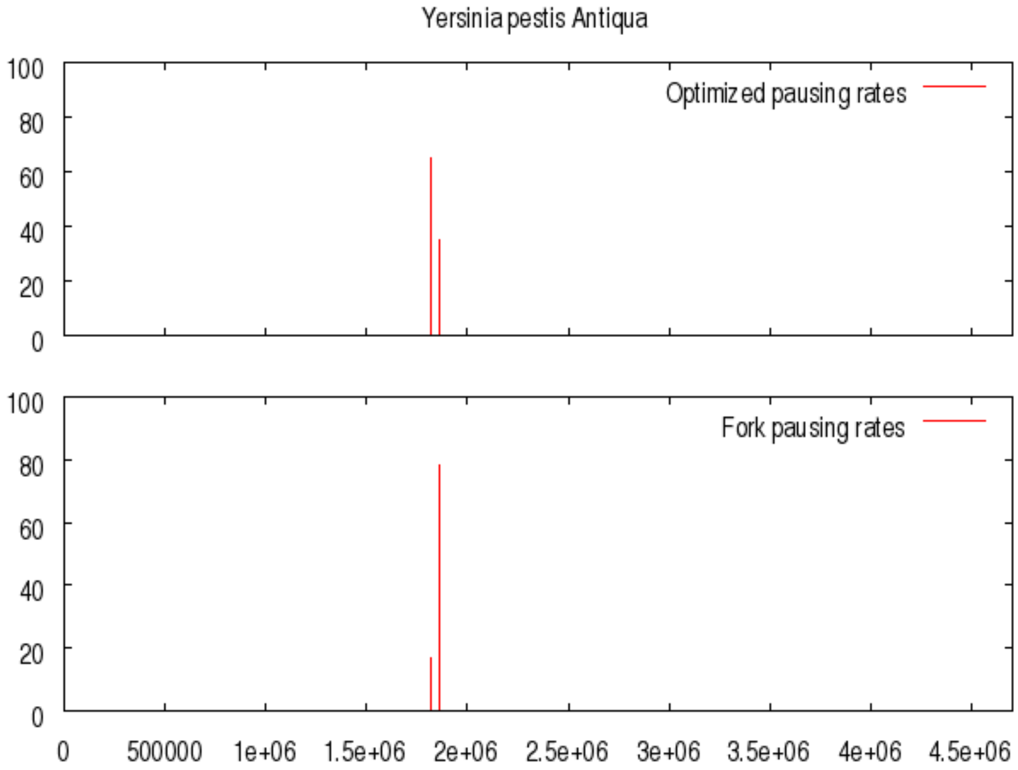
Yersinia pestis Antiqua
Optimized pausing rates
Fork pausing rates
0
20
40
60
80
100
0
500000
1e+06
1.5e+06
2e+06
2.5e+06
3e+06
3.5e+06
4e+06
4.5e+06

Yersinia pestis Nepal516

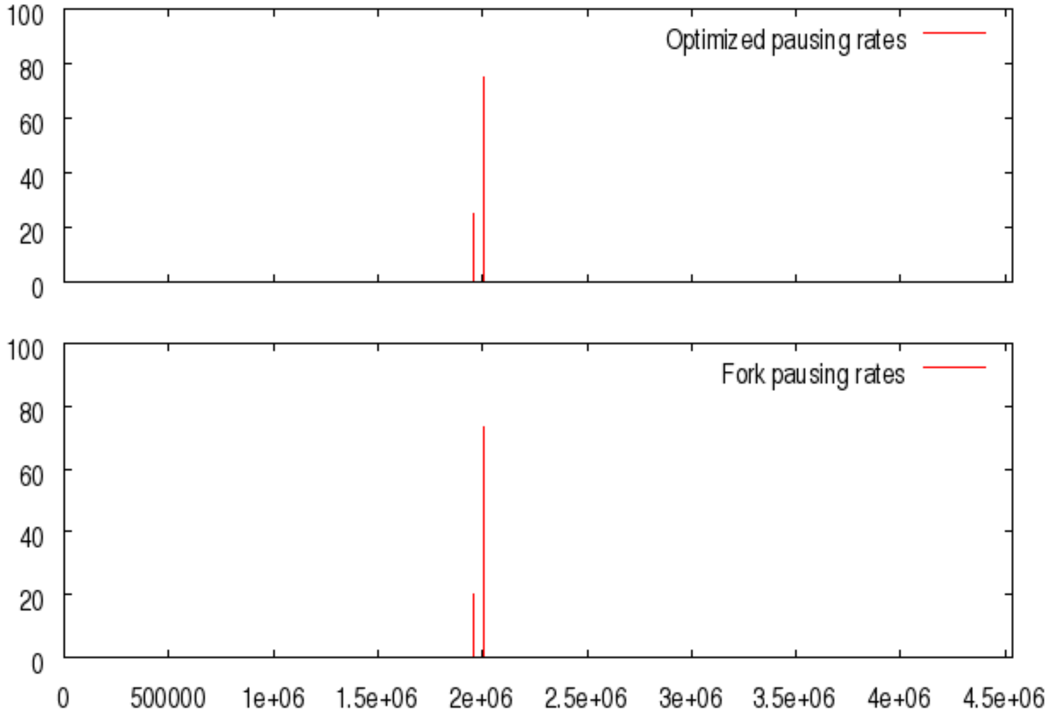

Escherichia coli UTI89

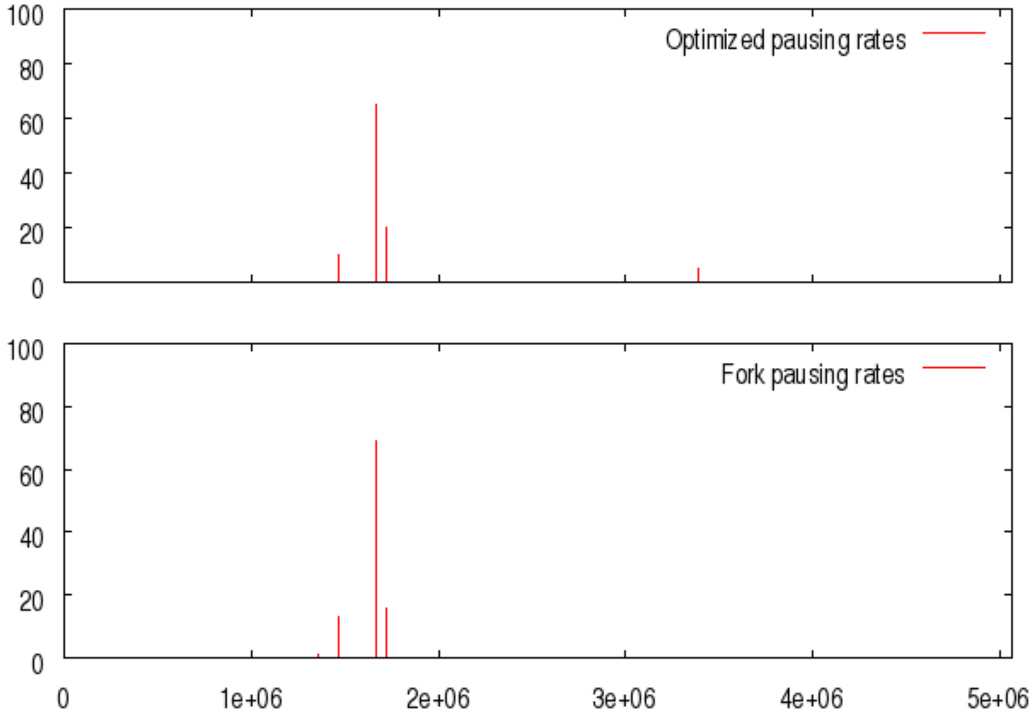

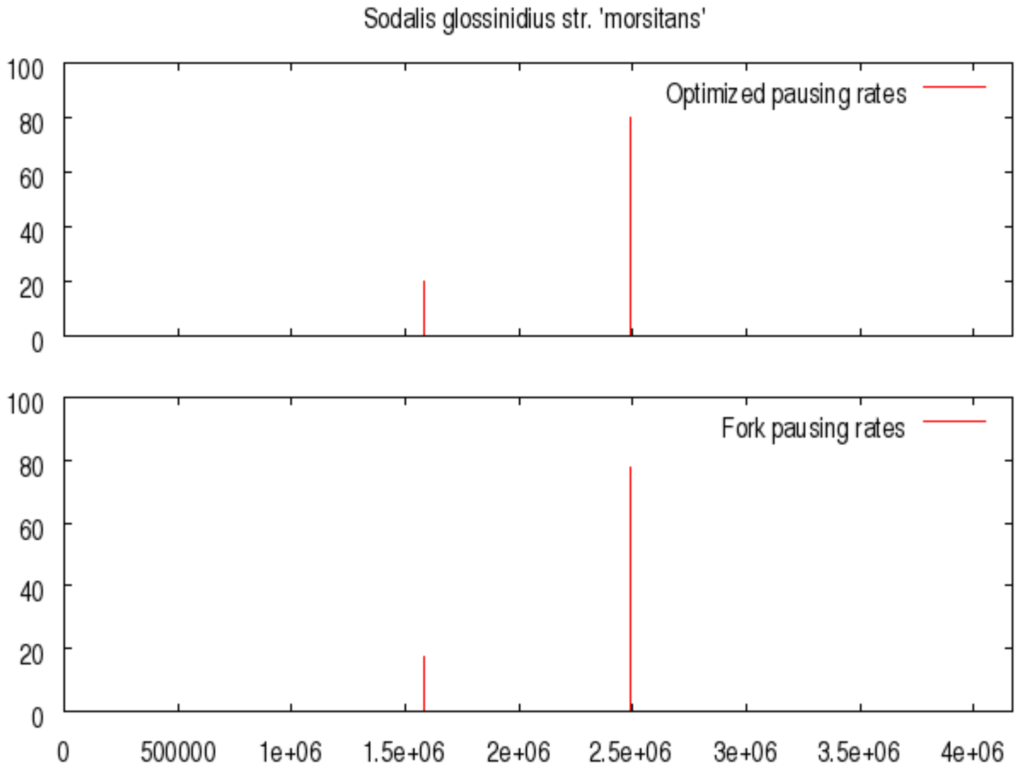
Sodalis glossinidius str. 'morsitans'
Optimized pausing rates
Fork pausing rates
0
20
40
60
80
100
0
500000
1e+06
1.5e+06
2e+06
2.5e+06
3e+06
3.5e+06
4e+06

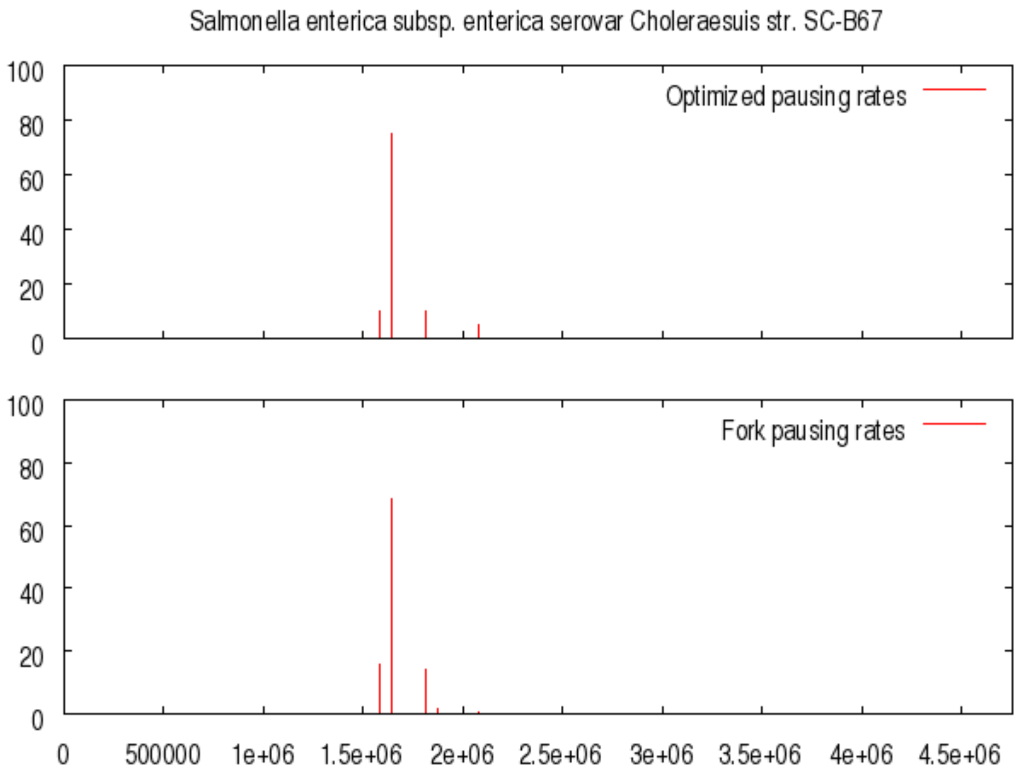
Salmonella enterica subsp. enterica serovar Choleraesuis str. SC-B67
Optimized pausing rates
Fork pausing rates
0
20
40
60
80
100
0
500000
1e+06
1.5e+06
2e+06
2.5e+06
3e+06
3.5e+06
4e+06
4.5e+06

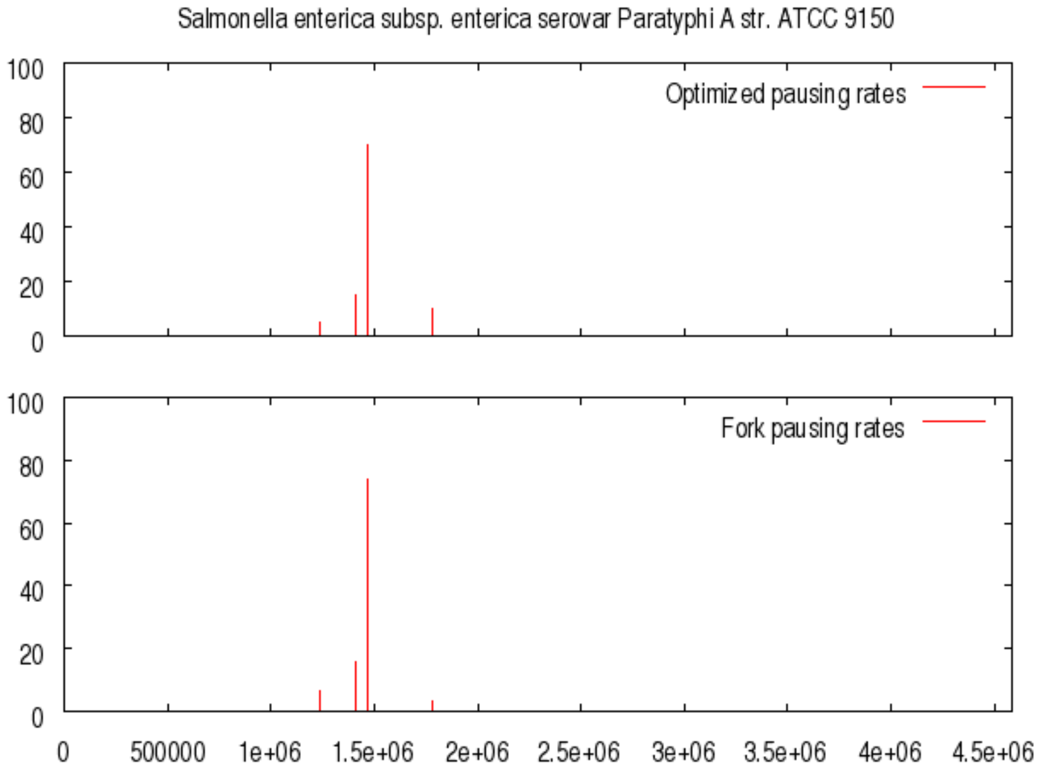
Salmonella enterica subsp. enterica serovar Paratyphi A str. ATCC 9150
Optimized pausing rates
Fork pausing rates
100
80
60
40
20
0
0
500000
1e+06
1.5e+06
2e+06
2.5e+06
3e+06
3.5e+06
4e+06
4.5e+06

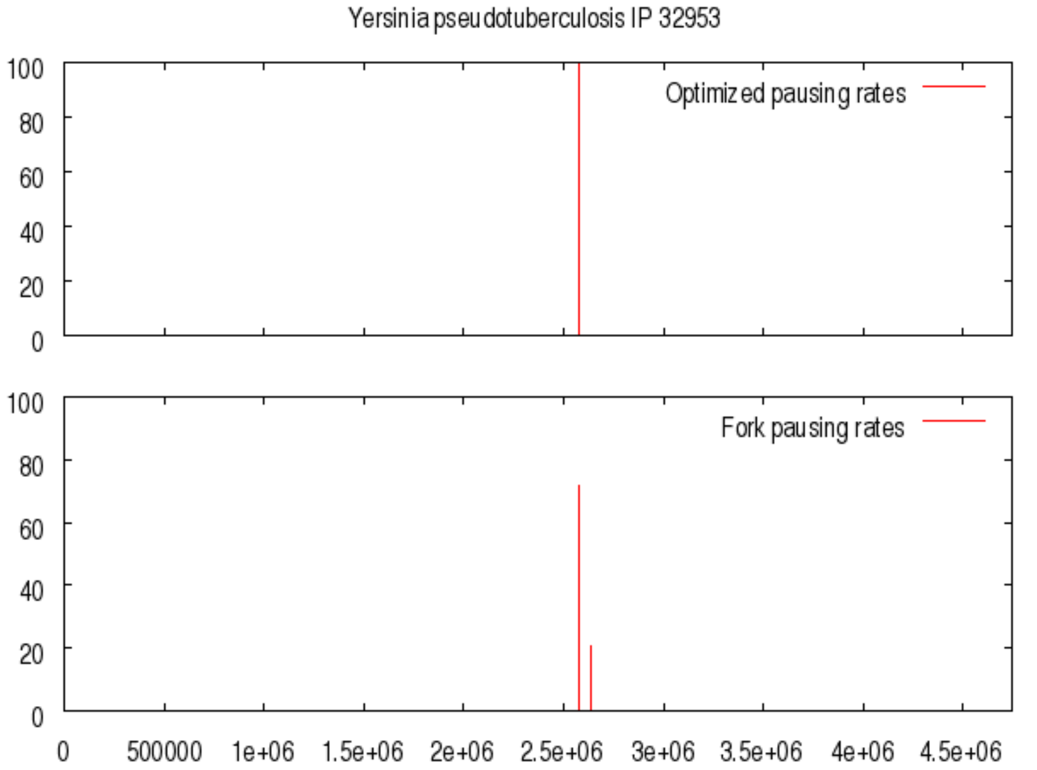
Yersinia pseudotuberculosis IP 32953
Optimized pausing rates
Fork pausing rates
100
80
60
40
20
0
0
500000
1e+06
1.5e+06
2e+06
2.5e+06
3e+06
3.5e+06
4e+06
4.5e+06

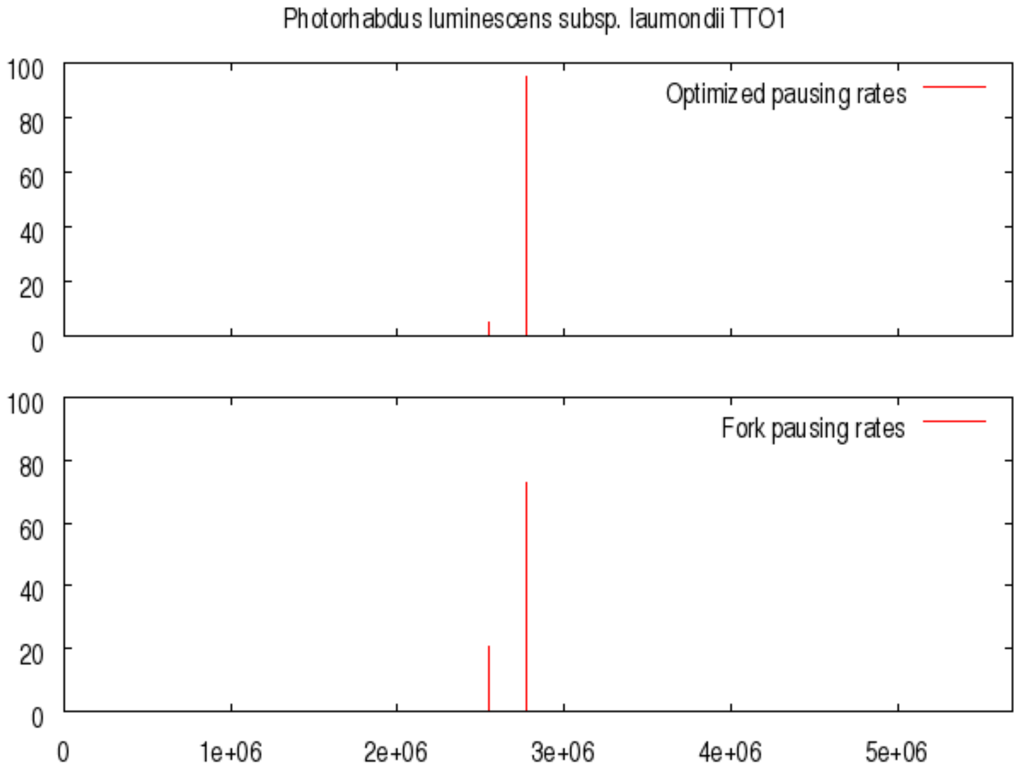
Photorhabdus luminescens subsp. laumondii TTO1
Optimized pausing rates
Fork pausing rates
100
80
60
40
20
0
0
1e+06
2e+06
3e+06
4e+06
5e+06

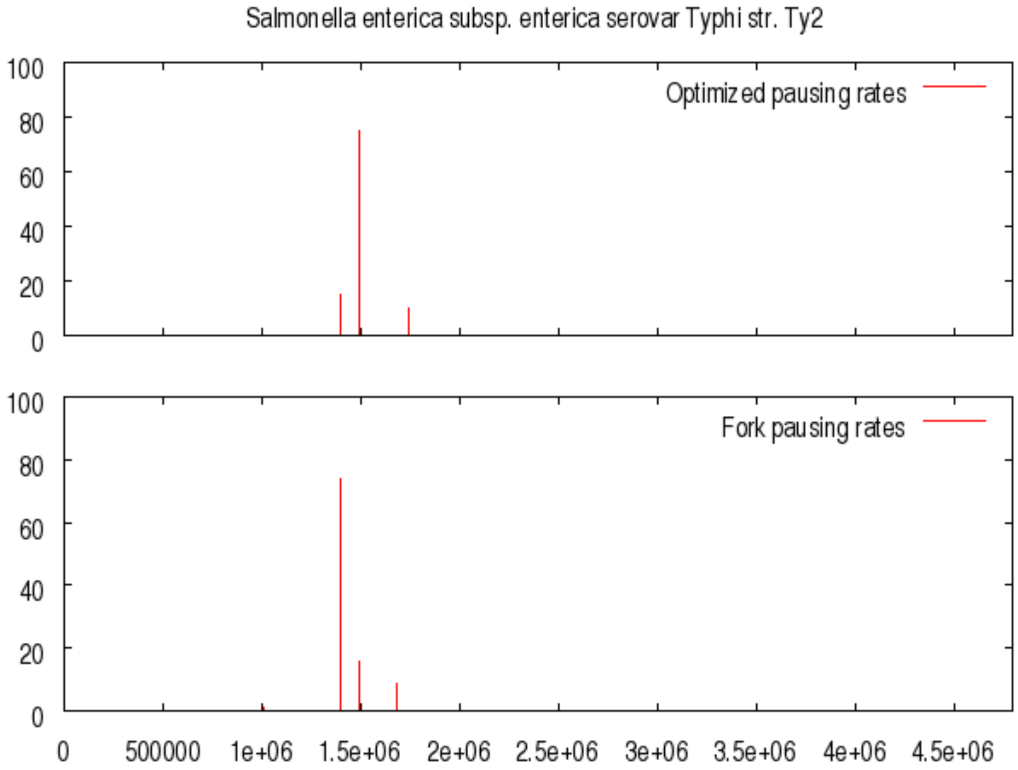
Salmonella enterica subsp. enterica serovar Typhi str. Ty2
Optimized pausing rates
Fork pausing rates
100
80
60
40
20
0
0
500000
1e+06
1.5e+06
2e+06
2.5e+06
3e+06
3.5e+06
4e+06
4.5e+06

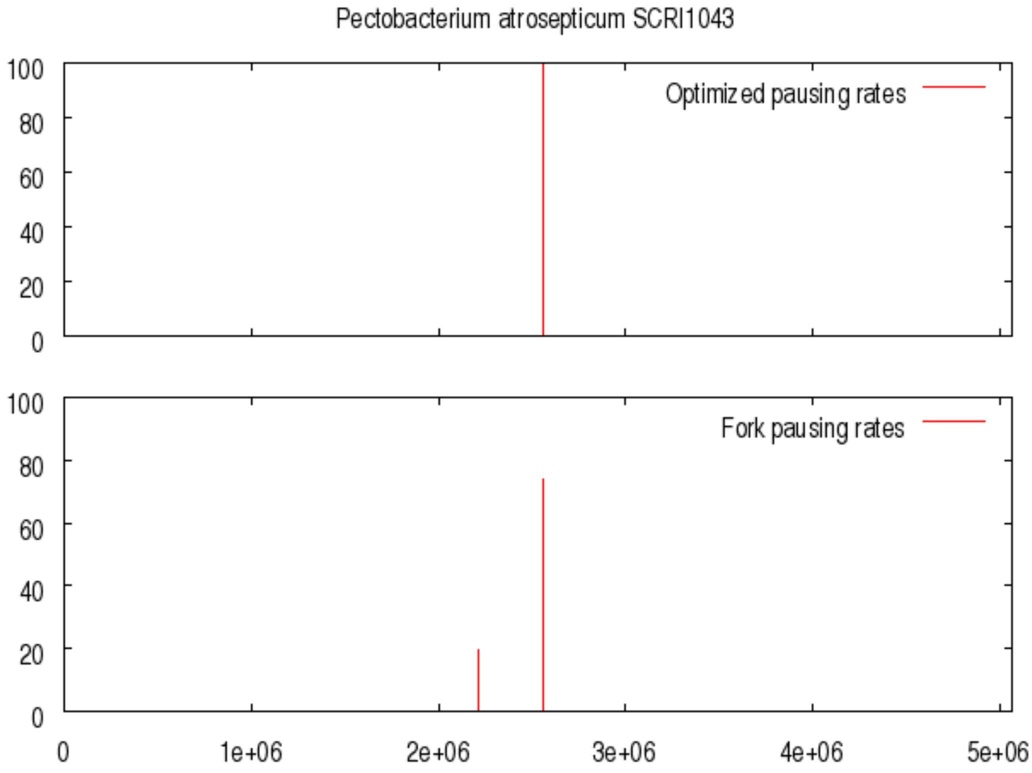
Pectobacterium atrosepticum SCRI1043
Optimized pausing rates
Fork pausing rates
0
20
40
60
80
100
0
1e+06
2e+06
3e+06
4e+06
5e+06

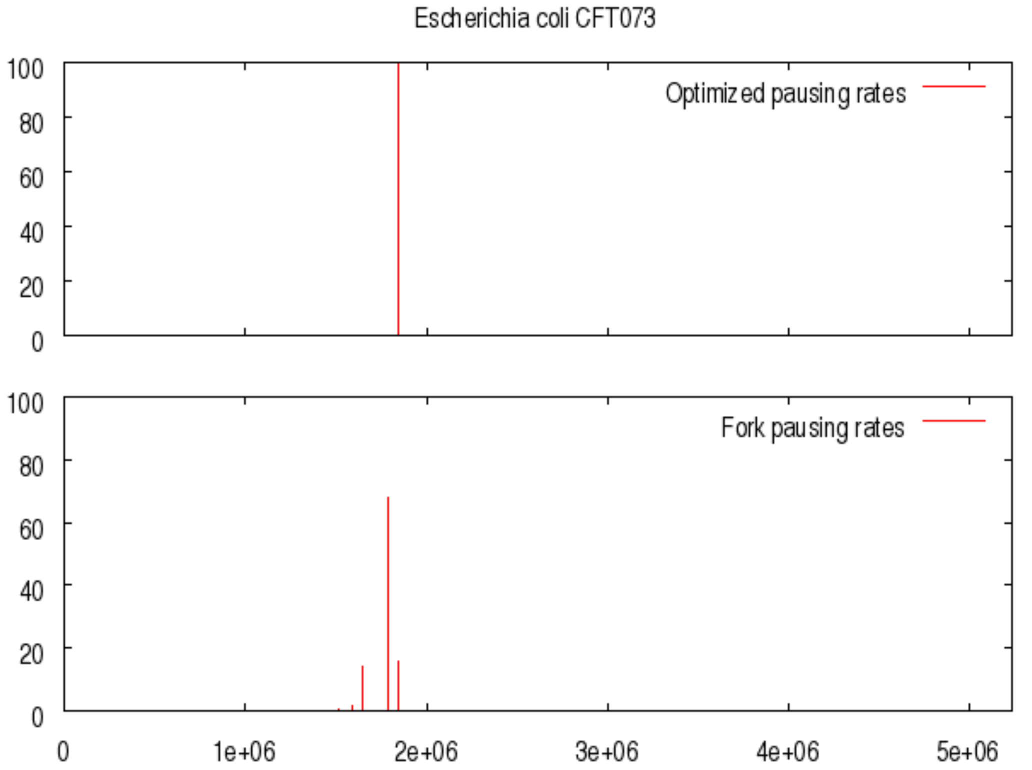
Escherichia coli CFT073
Optimized pausing rates
Fork pausing rates
0
20
40
60
80
100
0
1e+06
2e+06
3e+06
4e+06
5e+06

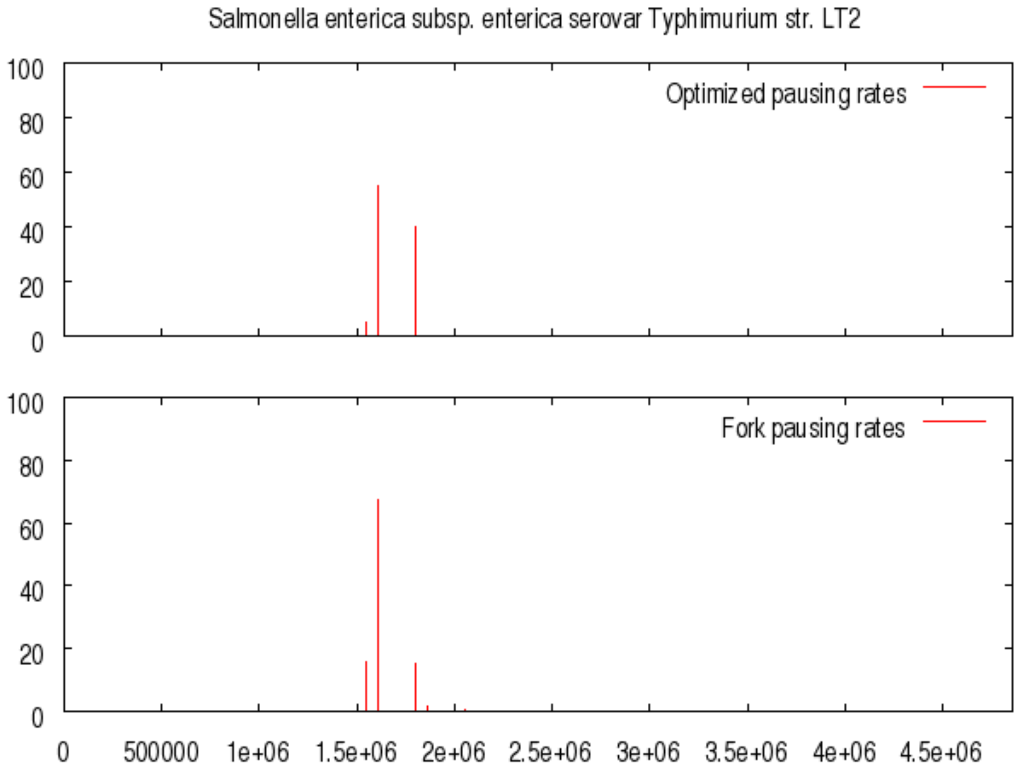
Salmonella enterica subsp. enterica serovar Typhimurium str. LT2
Optimized pausing rates
Fork pausing rates
0
20
40
60
80
100
0
500000
1e+06
1.5e+06
2e+06
2.5e+06
3e+06
3.5e+06
4e+06
4.5e+06

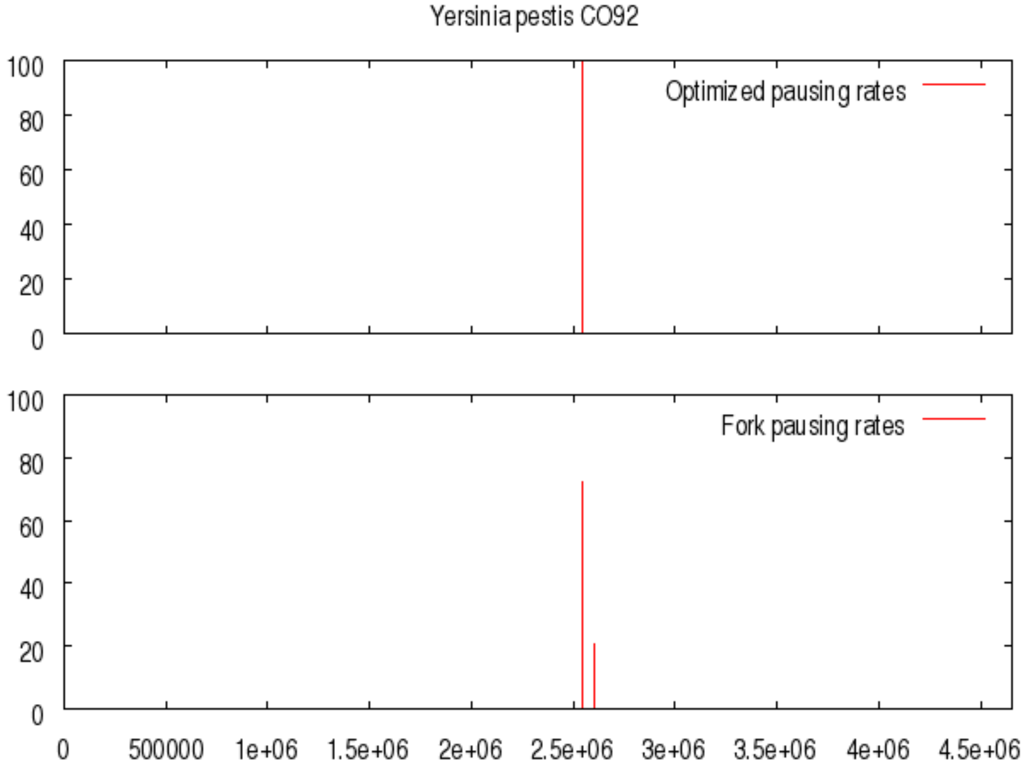
Yersinia pestis CO92
Optimized pausing rates
Fork pausing rates
0
20
40
60
80
100
0
500000
1e+06
1.5e+06
2e+06
2.5e+06
3e+06
3.5e+06
4e+06
4.5e+06

Escherichia coli O157:H7 str. Sakai

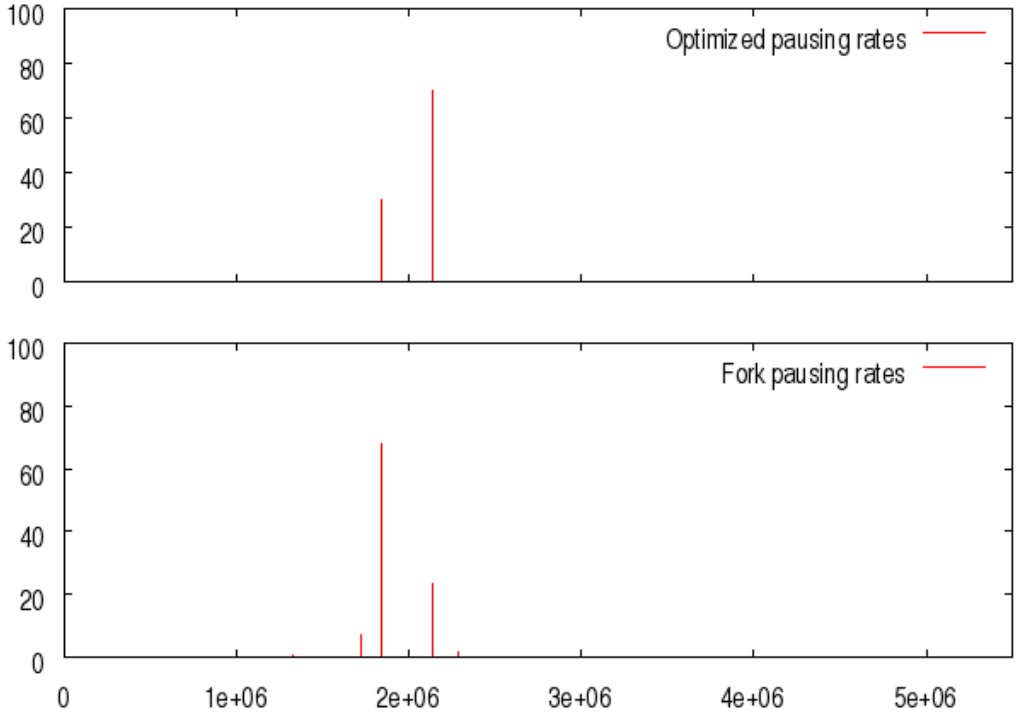

Escherichia coli O157:H7 str. EDL933

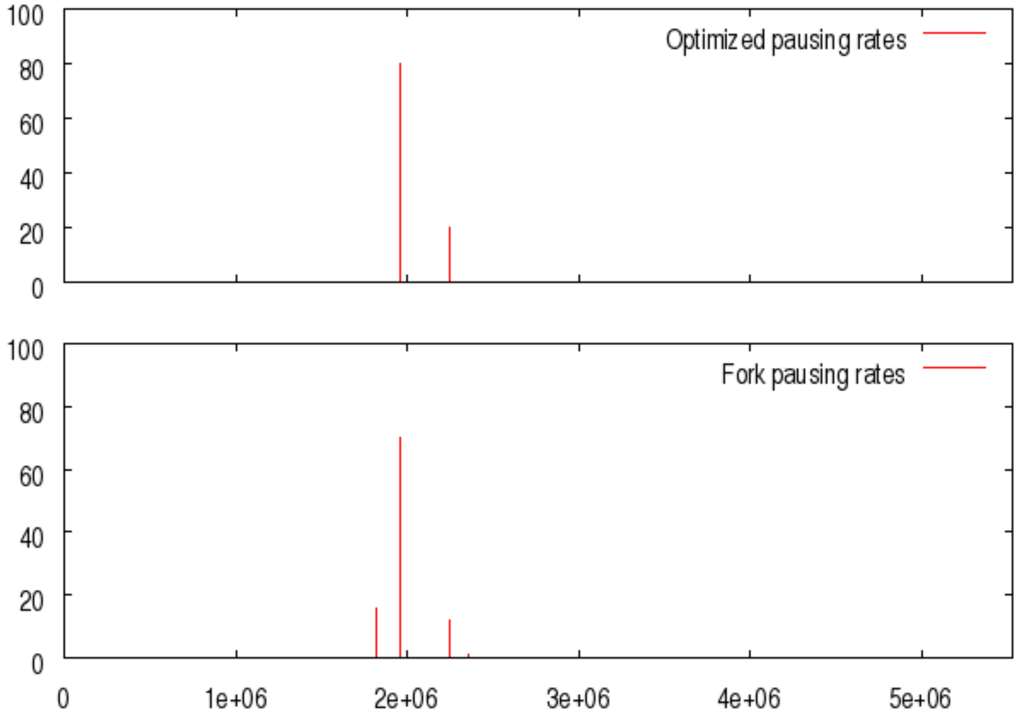

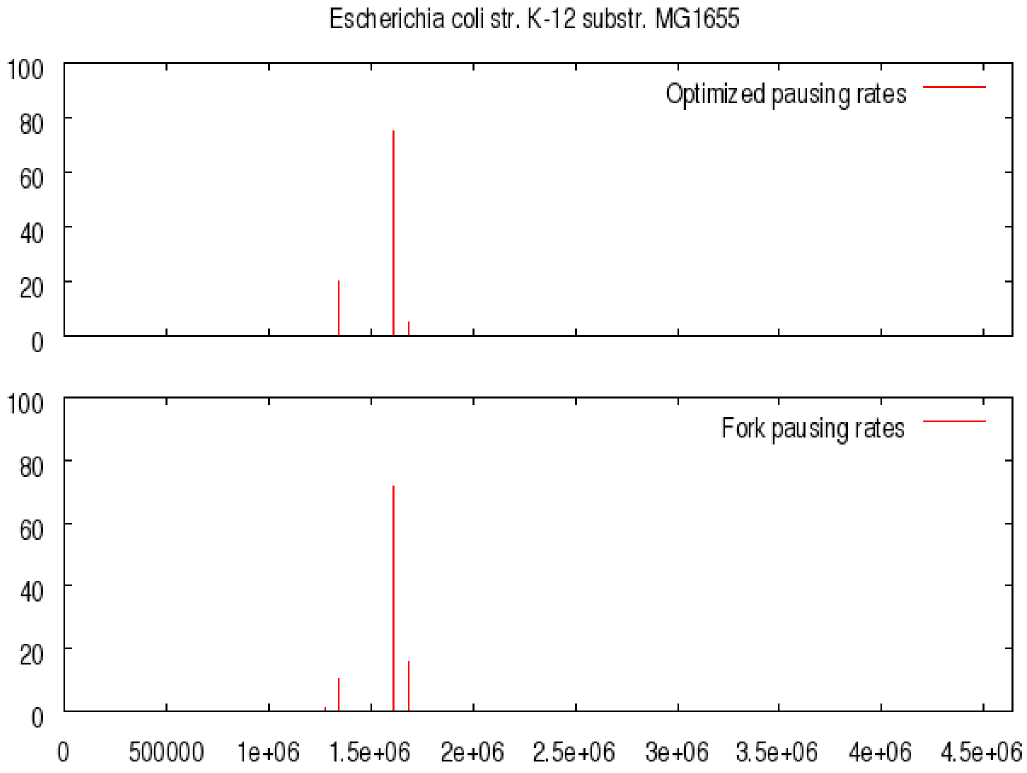

Escherichia coli str. K-12 substr. MG1655
Optimized pausing rates
Fork pausing rates
100
80
60
40
20
0
0
500000
1e+06
1.5e+06
2e+06
2.5e+06
3e+06
3.5e+06
4e+06
4.5e+06
